# The feline skin microbiota: The bacteria inhabiting the skin of healthy and allergic cats

**DOI:** 10.1371/journal.pone.0178555

**Published:** 2017-06-02

**Authors:** Caitlin E. Older, Alison Diesel, Adam P. Patterson, Courtney Meason-Smith, Timothy J. Johnson, Joanne Mansell, Jan S. Suchodolski, Aline Rodrigues Hoffmann

**Affiliations:** 1 Dermatopathology Specialty Service, Department of Veterinary Pathobiology, College of Veterinary Medicine and Biomedical Sciences, Texas A&M University, College Station, TX, United States of America; 2 Department of Small Animal Clinical Sciences, College of Veterinary Medicine and Biomedical Sciences, Texas A&M University, College Station, TX, United States of America; 3 Department of Veterinary and Biomedical Sciences, College of Veterinary Medicine, University of Minnesota, Saint Paul, MN, United States of America; Universite Paris-Sud, FRANCE

## Abstract

**Background:**

The skin is inhabited by a multitude of microorganisms. An imbalance of these microorganisms is associated with disease, however, the causal relationship between skin microbiota and disease remains unknown. To describe the cutaneous bacterial microbiota of cats and determine whether bacterial dysbiosis occurs on the skin of allergic cats, the skin surfaces on various regions of 11 healthy cats and 10 allergic cats were sampled.

**Methodology/Principal findings:**

Genomic DNA was extracted from skin swabs and sequenced using primers that target the V4 region of the bacterial 16S rRNA. The bacterial sequences from healthy cats revealed that there are differences in species diversity and richness between body sites and different epithelial surfaces. Bacterial communities preferred body site niches in the healthy cats, however, the bacterial communities on allergic cat skin tended to be more unique to the individual cat. Overall, the number of bacterial species was not significantly different between the two health status groups, however, the abundances of these bacterial species were different between healthy and allergic skin. *Staphylococcus*, in addition to other taxa, was more abundant on allergic skin.

**Conclusions/Significance:**

This study reveals that there are more bacterial species inhabiting the skin of cats than previously thought and provide some evidence of an association between dysbiosis and skin disease.

## Introduction

The body is colonized by a variety of microorganisms. These microorganisms can be beneficial, by educating the immune system and inhibiting the growth of pathogenic microorganisms, or they can be pathogenic, producing disease in affected tissues [1, 2]. The microbial populations present in and on the body vary with different anatomical locations [2, 3]. Likewise, they differ between individuals due to intrinsic and extrinsic factors, such as immune status and environment, respectively. Some studies have shown that molecular or cellular alterations in the skin [4, 5] and intestinal tract [6, 7] have an effect on the composition of the microbiota, however it is still unclear if changes in the cutaneous microbiota are triggered by disease or result in disease [2, 8]. Regardless, it appears that there is a relationship between alterations in the skin microbiota and disease [1, 9]. By studying the microbiota of skin in healthy individuals, a standard is set for what is “normal”, which can subsequently be used to understand how differences in these opportunistic microorganisms may be causing or contributing to infections or disease [2, 9].

Several studies have been performed to describe the microbiota of various body regions in humans, including the skin [2, 3, 10]. In veterinary medicine, fewer studies regarding the microbiota in animals have been performed, however this is a growing area of important research. There have been several studies describing the gastrointestinal [11–13] and oral microbiota of cats and dogs [14–18]. In the oral cavity of cats, Sturgeon et al. found a predominance of unidentified Pasteurellaceae, *Moraxella*, *Thermomonas*, and unclassified Comamonadaceae [16]. In dogs, they found *Porphyromonas* dominating the oral cavity [17]. A few studies have been conducted on the cutaneous microbiota of animals [9, 19–22]. In dogs, the cutaneous bacterial microbiota belongs to the phyla Proteobacteria, Actinobacteria and Firmicutes [9]. One study looked at how the microbiota in dogs change with atopic flares and treatments, and found that treatment results in normalization of the bacterial communities [19]. Dogs with allergic skin disease, such as atopic dermatitis (AD), are colonized by a different microbiota than healthy dogs [9, 19, 23, 24].

Studies have also looked at how the microbiota is shared between animals and their owners [14, 15, 18, 25]. Song et al. found that the skin microbiota of adults who own dogs are more similar to that of other dog owners than non-dog owning adults. Some results have indicated that living with cats and/or dogs, and thus sharing microbiotas, can help reduce the risk of developing allergies in people [26, 27]. However, the data for cat ownership is still conflicting [26, 28–30]. Current studies show that cat ownership could result in decreased [31] or increased [26, 29, 30] allergies in children, and some studies have failed to find any correlation between children allergies and cat ownership [32].

Although the human and canine skin microbiota has been described in multiple studies, the feline skin microbiota remains somewhat unknown. Most of the recent research on the skin microbiota of cats has focused on *Staphylococcus* due to its role in skin diseases [33, 34]. Previously, a culture based study was performed with the goal of describing the feline skin microbiota and found that *Micrococcus*, *Acinetobacter*, *Streptococci*, and *Staphylococci* dominated the sites sampled; however, bacteria could not be isolated from half of the samples, which the authors attributed to the normal grooming behavior of cats [22]. Recent studies have also examined the fungal mycobiota in healthy and allergic cats and dogs, which have found significant differences in the fungal communities colonizing the skin [20, 21].

The objectives of this study were to describe the bacterial microbiota present on different skin surfaces of healthy cats and identify differences between healthy cats and cats with allergic skin disease. We hypothesized that next-generation sequencing of feline skin swabs would reveal a preference of specific bacterial microbiota to different body sites. Furthermore, we expected to see differences in the diversity and specific bacterial taxa present on the skin of allergic cats compared to those without skin disease.

## Materials and methods

This study had been approved by the Texas A&M University (TAMU) Institutional Animal Care and Use Committee under 2012–139. Informed consent to enroll clinical cases into the study was obtained from each client.

### Participants

Collection of samples from all animals enrolled in this study followed a protocol approved by the Texas A&M University Institutional Animal Care and Use Committee.

#### Healthy cats

Eleven healthy cats participated in this study with their ages ranging from 2 to 17 years old (Table 1). Included were 6 spayed females (3 domestic shorthairs (DSH), 1 domestic medium hair (DMH), 2 domestic long hairs (DLH)) and 5 neutered males (3 DSH, 1 DMH, 1 DLH). All of these cats lived with other animals. Six of the cats were kept indoors, three spent time both inside and outside, and one was kept solely outdoors. All cats were evaluated by a board certified dermatologist, and none had skin lesions, history of pruritus or any history of skin disease in the past 6 months. These patients were not treated with antibiotics, antifungals, anti-inflammatory or immunosuppressive drugs for at least 6 months prior to sample collection. Fleas were not present on any of the cats at the time of sample collection.

**Table 1:** Signalment and medical histories of cats enrolled in this study.

Cat	Health status	Breed	Age	Sex	Fleas	Time Indoors	Indoor Environment	Outdoor Environment	Previous antibiotics usage
**F1**	Healthy	DLH	5	CM	Y	100	n/a	n/a	N
**F2**	Healthy	DSH	2	SF	N	100	TFB	n/a	N
**F3**	Healthy	DSH	13	CM	N	100	CTFB	n/a	N
**F4**	Healthy	DSH	7	CM	N	70	TFB	TGW	N
**F5**	Healthy	DMH	4.5	SF	N	99	CTFB	TGW	N
**F6**	Healthy	DSH	7	SF	N	100	TFB	n/a	N
**F7**	Healthy	DSH	9.5	SF	N	50	B	TGW	N
**F8**	Healthy	DLH	13	SF	N	100	CTFB	n/a	N
**F9**	Healthy	DLH	15	SF	Y	0	n/a	TGW	N
**F10**	Healthy	DMH	6	CM	N	100	CTFB	n/a	N
**F11**	Healthy	DSH	17	CM	N	100	CTF	n/a	N
**F12**	Allergic	DSH	9	CM	N	100	TFB	n/a	N
**F13**	Allergic	Sia	8	CM	N	100	TFB	n/a	N
**F14**	Allergic	DSH	11	CM	Y	95	CFB	TGW	N
**F15**	Allergic	Sia	9	SF	N	100	TFB	n/a	N
**F16**	Allergic	DSH	5	SF	N	60	CTFB	TGW	Y
**F17**	Allergic	DSH	9	SF	N	100	CTFB	n/a	N
**F18**	Allergic	Per	4	CM	Y	100	CTB	n/a	Y
**F19**	Allergic	DSH	11	SF	Y	95	CF	TG	Y
**F20**	Allergic	DSH	7	SF	N	100	CTFB	n/a	N
**F21**	Allergic	DSH	8	SF	Y	95	TFB	TGW	N

Abbreviations. Signalment: DLH-Domestic long hair, DMH-Domestic medium hair, DSH-Domestic short hair, Per-Persian, Sia-Siamese, CM-Castrated male, SF-Spayed female. Indoor environment: C-Carpet, T-Tile, F-Furniture, B-Bedding. Outdoor environment: T-Trees, G-Grass, W-Weeds. Y-Yes, N-No.

Modified from Meason-Smith C, Diesel A, Patterson AP, Older CE, Johnson TJ, Mansell JM, et al. Characterization of the cutaneous mycobiota in healthy and allergic cats using next generation sequencing. Vet Dermatol. 2016:1–11.

#### Allergic cats

Ten cats with allergic skin disease were enrolled in this study with ages ranging from 5 to 11 years old (Table 1). Included were 5 spayed females (4 DSH and 1 Siamese), 1 intact female (DSH), 3 castrated males (2 DSH and 1 Siamese), and 1 intact male (Persian). All but two of these cats lived with or had contact with other animals. Six of the cats were kept indoors and four spent time both inside and outside. All allergic cats were evaluated by a board certified veterinary dermatologist. Allergic cats in this study were defined as those with a history of pruritus/over-grooming and/or that showed common cutaneous reaction patterns (self-induced alopecia, miliary dermatitis, eosinophilic skin lesions, and/or cervicofacial dermatitis). All parasitic and infectious causes of pruritus had been ruled out by standard diagnostic and therapeutic methods. Allergic cats included animals with flea allergy dermatitis, cutaneous adverse food allergies, and feline non-flea non-food hypersensitivity reactions and were allowed to be maintained clinically on various medications according to their specific disease (e.g. adulticidal flea prevention, hypoallergenic diet, anti-inflammatory medications, allergen-specific immunotherapy). None of the cats presented with flea or ear problems. Three cats (F16, F18, F19) were treated with antibiotics prior to the study, however not within at least 1 month of sample collection.

### Sample collection

Skin swabs were collected from 12 sites from the healthy cats: axilla, chin, conjunctiva, dorsal nose, ear canal, groin, interdigital skin, lumbar region, nostril, oral cavity, pinna, and reproductive tract (prepuce or vulva). Skin swabs were collected from 6 sites from the allergic cats: axilla, ear canal, groin, interdigital skin, lumbar region, and nostril. Three Isohelix buccal swabs (Cell Projects Ltd., Kent, UK) were used per skin site. Each side of the swab was rubbed on the skin 10 times. Two swabs were collected in a MoBio PowerBead tube with 750 μl buffer containing guanidine thiocyanate as well as additional proprietary reagents (MoBio Laboratories, Carlsbad, CA). Buffers containing guanidine thiocyanate have been previously described to preserve nuclei acids in field samples for up to 41 days without refrigeration [35]. Another swab was added to a 2 ml collection tube without any reagents. All samples were immediately stored for no longer than 30 days at 4°C until extractions were performed.

### DNA extraction and sequencing

Genomic DNA was extracted from skin swabs using the MoBio PowerSoil DNA Isolation Kit according to the manufacturer’s protocol. Extractions were also performed on unused Isohelix buccal swabs and empty PowerBead tubes to serve as negative controls. Extracted DNA and control samples were sequenced on an Illumina MiSeq instrument at the University of Minnesota Genomics Center using forward primer 515F: GTGYCAGCMGCCGCGGTAA and reverse primer 806R: GGACTACNVGGGTWTCTAAT that target the 16S rRNA gene [36].

### Data analysis

The raw sequences were processed using QIIME v1.9.1 [37] to perform quality filtering, definition of operational taxonomic units (OTUs) (sequences with 97% similarity) [38], and taxonomic classification with the Greengenes database [39–42]. Suspected contaminants found in the controls were removed by filtering them from the OTU table as previously described [14]. For the purpose of comparing different physiologies of the sites sampled, the chin was considered “sebaceous”; the axilla, dorsal nose, ear canal, groin, interdigital, lumbar, and pre-aural space were considered “haired skin”; the conjunctiva, nostril, and reproductive were considered “mucosal” or “mucocutaneous junctions”; and the oral cavity was considered unique and referred as “oral”. Alpha diversity (number of bacterial species) was calculated using Chao1, Shannon, and observed OTUs metrics to determine species richness and evenness of each sample. Beta diversity (bacterial community composition) was calculated using weighted UniFrac, unweighted UniFrac, and Bray-Curtis metrics to measure similarity between samples; resulting plots were viewed using EMPeror [43].

The analysis of similarities (ANOSIM) function of PRIMER 7 (PRIMER-E Ltd., Luton, UK) was performed on the distance matrices from beta diversity calculations to assess differences in bacterial community composition. A Kruskal-Wallis test was performed using JMP Pro 11 (SAS Institute Inc., Cary, NC) to assess differences in the alpha diversity and relative abundance of bacterial taxa. P-values were corrected for false discovery rate, as previously described [44]. The relative abundance tables were filtered and linear discriminant analysis (LDA) effect size (LEfSe) performed with an alpha value of 0.01 to analyze the difference in taxa abundance with the different variables (cat number, body site, physiology, health condition) [45].

## Results

From the 192 samples from healthy and allergic cats, 5 samples (2 from healthy cats, 3 from allergic cats) were excluded from the analysis due to low sequence counts. Seven taxa (Xanthomonadaceae, 0319-6G20, Comamonadaceae, Beijerinckiaceae, Thermoactinomycetaceae, Weeksellaceae, and unassigned Rhizobiales) were very abundant in the negative control samples. These taxa and Cyanobacteria were filtered out of the OTU tables prior to downstream analysis [14].

### Healthy cats

The OTU table was rarefied to 3100 sequences. The average number of sequences per sample was 34378. A total of 8137 OTUs were identified with 31 resulting phyla (S4 Table).

#### Species richness and diversity

**Fig 1:**
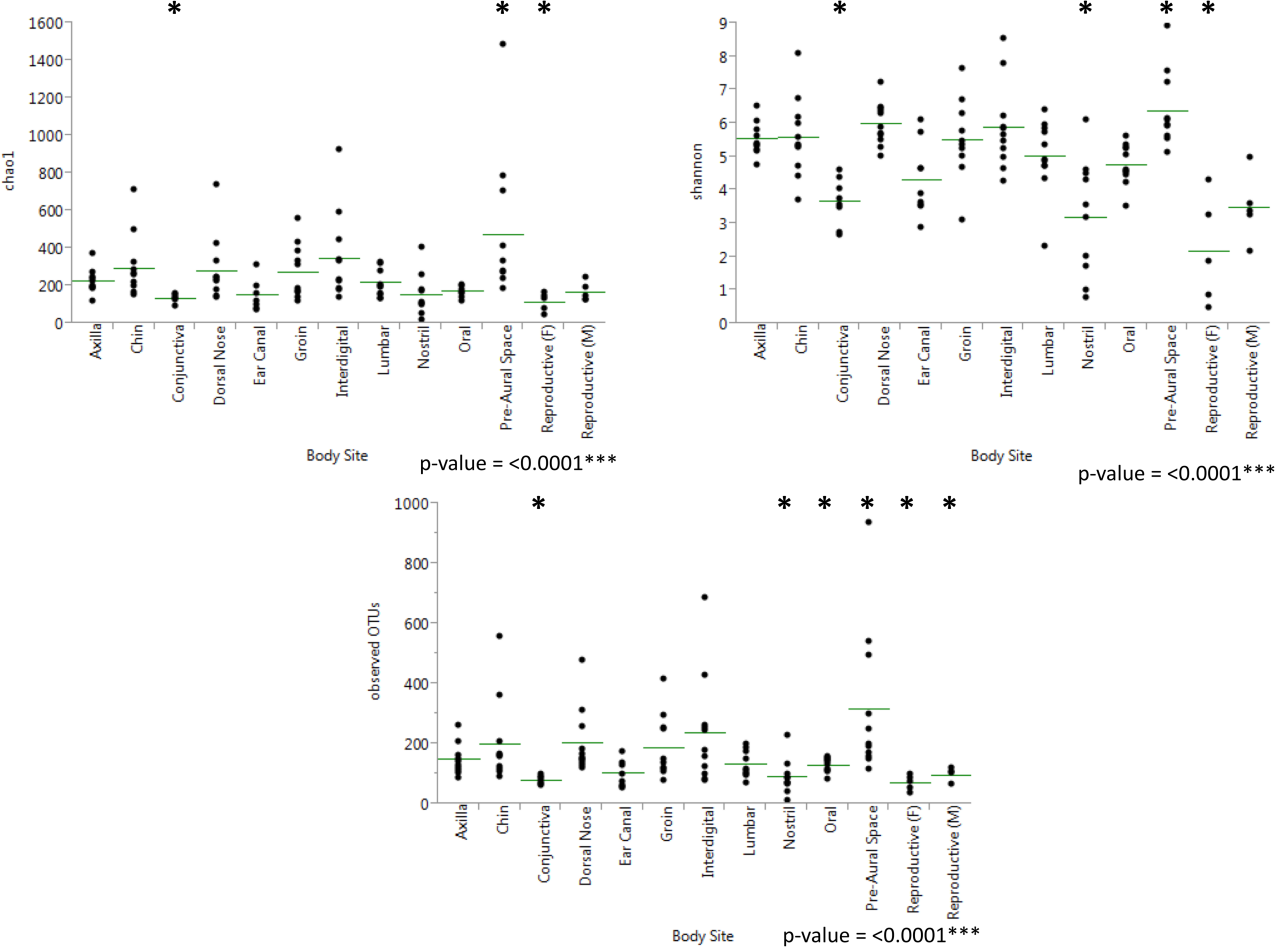
Results from Kruskal-Wallis tests comparing alpha diversity results by body site in healthy cats. Results from pairwise Kruskal-Wallis tests on body sites in healthy cats at a rarefaction of 3100 sequences per sample. Asterisks indicate sites that were found to be significantly different from at least 8 other body sites.

The samples from the healthy cats were compared based on cat number, body site, and physiology of the different sites. The three alpha diversity metrics used included Shannon, Chao1 and observed OTUs (Table 2). Observed OTUs gives insight into the richness of the microbial communities present, Chao1 estimates richness at full sequencing coverage, while Shannon takes into account richness and evenness. The pre-aural space was the site with the greatest richness and even distribution of these species, while the mucosal sites (reproductive, nostril, and conjunctiva) and ear canal had the lowest alpha diversity (Fig 1). Out of the skin physiologies compared (haired, mucosal, oral, and sebaceous), haired skin had the highest richness and evenness, while mucosal surfaces had the lowest (S1 Fig). Kruskal-Wallis tests revealed that all three alpha diversity metrics resulted in significant differences in the number of observed OTUs and the richness of the populations present when comparing individual cats, different body sites, and different physiologies (S1 Table).

**Table 2:** Alpha diversity averages measures at 3100 sequences per sample for healthy cats.

	**Chao1**	**Observed OTUs**	**Shannon**
**Body Site**			
Axilla	226 (59)	149 (48)	5.53 (0.46)
Chin	296 (163)	201 (132)	5.59 (1.13)
Conjunctiva	133 (19)	78 (12)	3.66 (0.65)
Dorsal Nose	282 (164)	203 (104)	6.00 (0.60)
Ear Canal	155 (71)	103 (40)	4.30 (1.02)
Groin	272 (137)	187 (99)	5.52 (1.10)
Interdigital	348 (222)	237 (174)	5.87 (1.22)
Lumbar	221 (74)	134 (40)	5.02 (1.05)
Nostril	152 (106)	90 (56)	3.18 (1.66)
Oral	175 (26)	129 (22)	4.78 (0.59)
Pre-aural space	471 (372)	319 (237)	6.39 (1.05)
Reproductive (F)	115 (44)	72 (23)	2.16 (1.43)
Reproductive (M)	169 (48)	94 (22)	3.48 (0.89)
**Cat**			
C1	185 (78)	132 (56)	4.69 (1.48)
C2	235 (96)	162 (76)	4.94 (1.60)
C3	180 (39)	122 (33)	4.77 (1.07)
C4	185 (64)	112 (51)	4.33 (1.19)
C5	294 (157)	204 (109)	5.42 (1.05)
C6	450 (393)	298 (261)	5.93 (1.98)
C7	179 (68)	129 (50)	4.94 (0.63)
C8	204 (80)	115 (41)	4.29 (1.45)
C9	452 (269)	306 (196)	6.05 (2.03)
C10	162 (38)	109 (34)	4.63 (0.78)
C11	220 (132)	143 (96)	4.79 (1.74)
**Skin Physiology**			
Haired	303 (219)	205 (149)	5.72 (1.05)
Mucosal	146 (72)	89 (39)	3.46 (1.38)
Oral	175 (26)	129 (22)	4.78 (0.59)
Sebaceous	296 (163)	201 (132)	5.59 (1.13)

Values represent averages with standard deviations in parenthesis.

#### Microbial community structure

**Fig 2:**
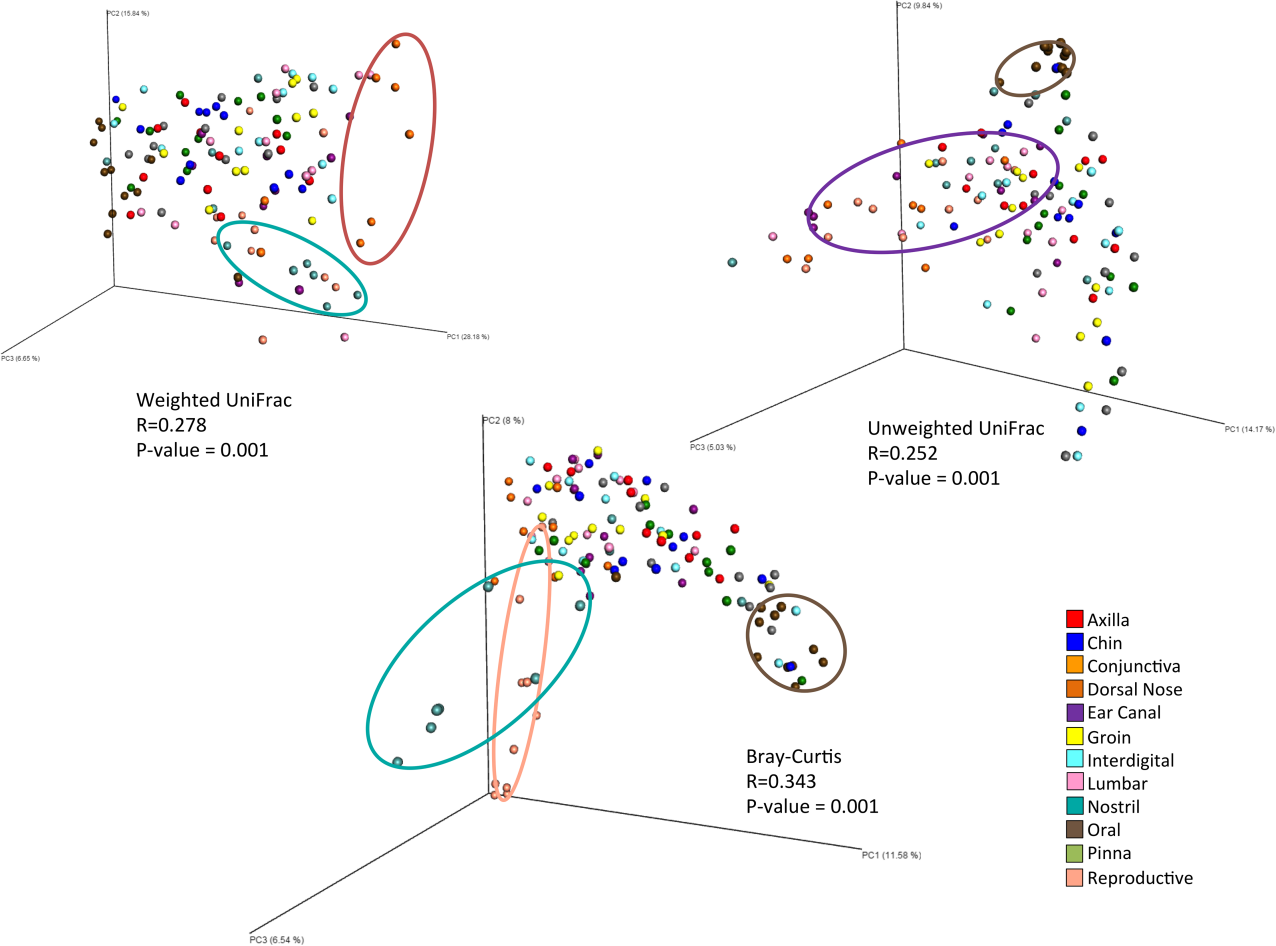
Principal coordinate analysis plots comparing body sites sampled in healthy cats. Principal coordinate analysis plots of weighted UniFrac, unweighted UniFrac, and Bray-Curtis distance matrices. Significant clustering was seen with all three measures of beta diversity.

**Fig 3:**
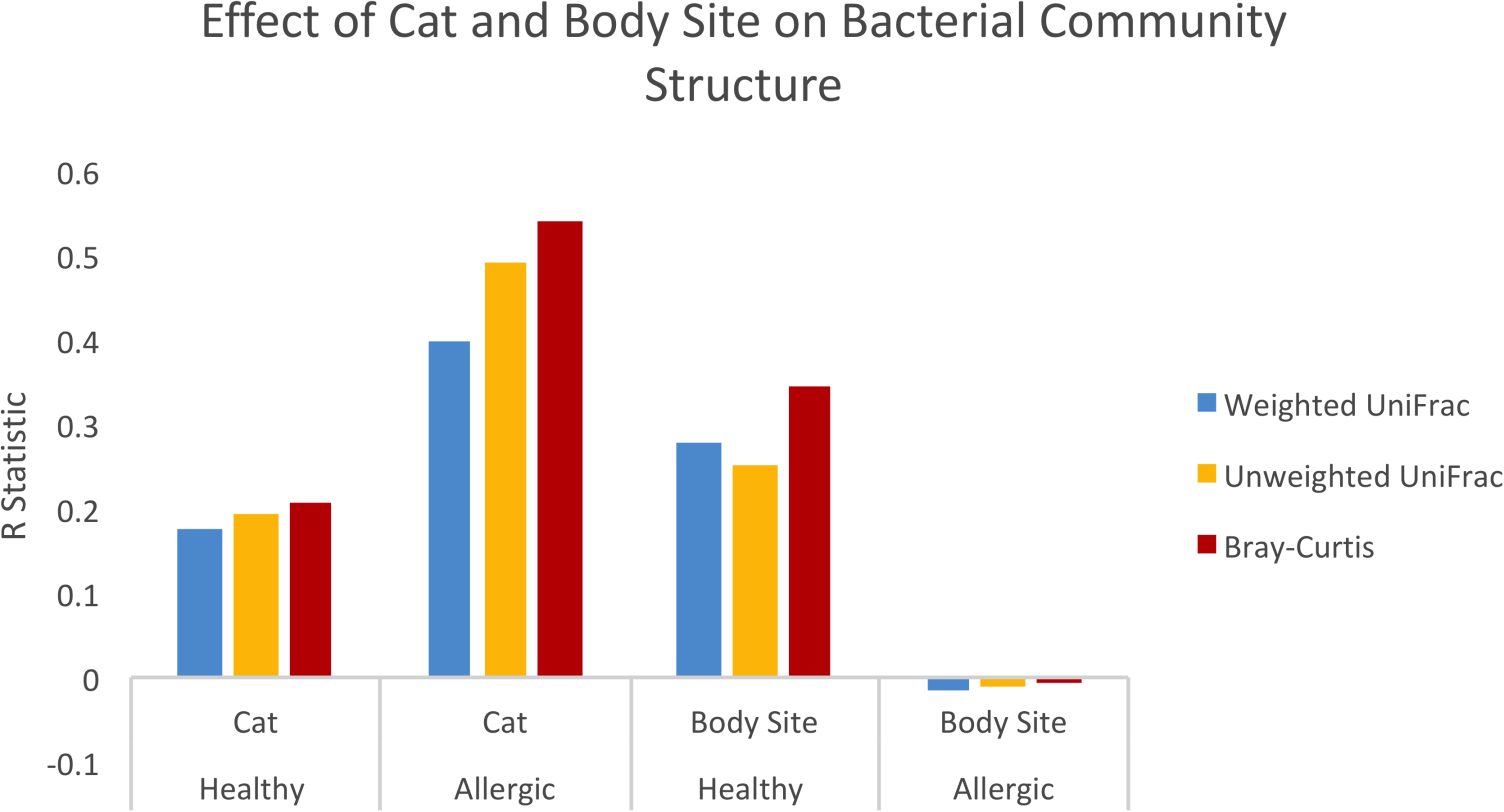
Effect of cat number and body site on community structure. ANOSIM of beta diversity distance matrices resulted in the shown R values. R values range from -1 to 1, with values closer to 0 indicating compared groups are very similar. Allergic cats clustered most by individual cat while healthy cats clustered most by body site.

Beta diversity measures (weighted UniFrac, unweighted UniFrac, and Bray-Curtis) indicated that body site had an impact on difference in community structure between samples (S2 Table). All three beta diversity metrics showed significance in dissimilarity when comparing the different sites sampled overall (R = 0.278, R = 0.252, R = 0.343 respectively, all with p = 0.001) (Fig 2) and with pairwise comparisons (S3 Table). Some clustering was observed when comparing individual cats, however less so than with body site (R = 0.175, R = 0.195, R = 0.209 respectively, with p = 0.001 for all) (Fig 3). Comparing the samples based on physiology of the sampled site resulted in significant differences in community structure based on weighted UniFrac (R = 0.320, p = 0.001) and Bray-Curtis (R = 0.297, p = 0.001) beta diversity measures. However, the chin, which was analyzed as the sebaceous site, did not cluster differently from the haired sites (S2 Fig).

#### Microbial community composition

**Fig 4:**
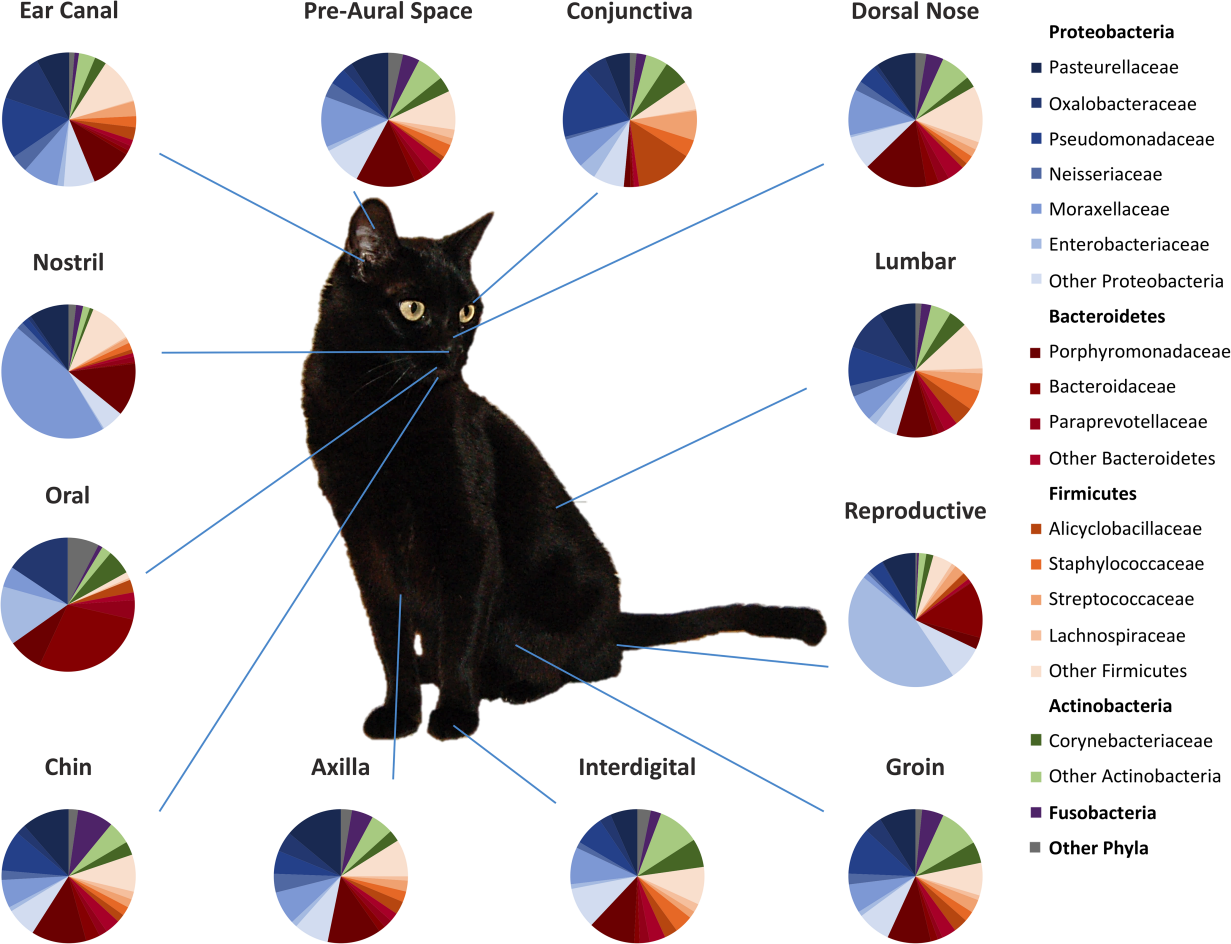
Relative taxa abundance at each body site sampled in healthy cats. Average relative taxa abundances at each body site (axilla, chin, conjunctiva, dorsal nose, ear canal, groin, interdigital, lumbar, nostril, oral, pre-aural space, and reproductive organs) sampled in healthy cats. Four phyla (Proteobacteria, Bacteroidetes, Firmicutes, and Actinobacteria) accounted for 97.5% of the bacteria found in the samples.

Overall, the most common phyla in cats were found to be Proteobacteria (46.4%), followed by Bacteroidetes (20.7%), Firmicutes (17.7%), Actinobacteria (8.6%), and Fusobacteria (4.1%) (Fig 4). The most common families sequenced were Porphyromonadaceae, Moraxellaceae, Pasteurellaceae, and Pseudomonadaceae. Using a pairwise Kruskal-Wallis test, we found that many taxa were significantly different in abundance based on physiology, body site, and/or individual cat. Differential taxa abundance was also seen between female and male cats at the reproductive site (S3 Fig). LEfSe did not reveal any significant differences in taxa abundances between cats, body sites, or physiologies.

### Allergic cats

The OTU table was rarefied to 2900 sequences. The average number of sequences per sample was 36490. A total of 4374 OTUs were identified with 19 resulting phyla.

#### Species richness and diversity

All alpha diversity metrics (Chao1, Shannon, observed OTUs) indicated significant differences between individual cats (p = 0.0354, p = 0.0231, and p = 0.0051 respectively) (Table 3). When comparing the two physiologies of the sites sampled in the allergic cats (haired skin and mucosal), the Shannon index showed significant differences in evenness with p = 0.0103. No significance in alpha diversity was seen when comparing the body sites sampled overall, however some significance was found in pairwise comparison tests.

**Table 3:** Alpha diversity averages at 2900 sequences per sample for allergic cats.

	**Chao1**	**Observed OTUs**	**Shannon**
**Body Site**			
Axilla	204 (72)	138 (53)	4.81 (1.62)
Ear Canal	80 (69)	119 (52)	4.08 (1.85)
Groin	216 (35)	137 (32)	5.04 (0.48)
Interdigital	264 (131)	173 (96)	4.90 (1.79)
Lumbar	215 (77)	139 (46)	5.09 (0.62)
Nostril	170 (63)	104 (38)	3.48 (1.57)
**Cat**			
C12	204 (58)	105 (29)	4.75 (0.52)
C13	236 (61)	161 (37)	4.87 (1.02)
C14	295 (131)	208 (100)	5.35 (1.77)
C15	208 (62)	131 (47)	5.14 (0.50)
C16	225 (44)	127 (27)	4.75 (0.57)
C17	239 (66)	157 (44)	5.22 (1.13)
C18	183 (42)	139 (32)	4.06 (1.25)
C19	60 (36)	44 (23)	1.36 (1.59)
C20	195 (11)	132 (14)	5.13 (0.21)
C21	218 (46)	134 (28)	4.90 (1.30)
**Skin Physiology**			
Haired	225 (90)	147 (64)	4.96 (1.28)
Mucosal	175 (66)	111 (46)	3.78 (1.74)

Values represent averages with standard deviations in parenthesis.

#### Microbial community structure

**Fig 5:**
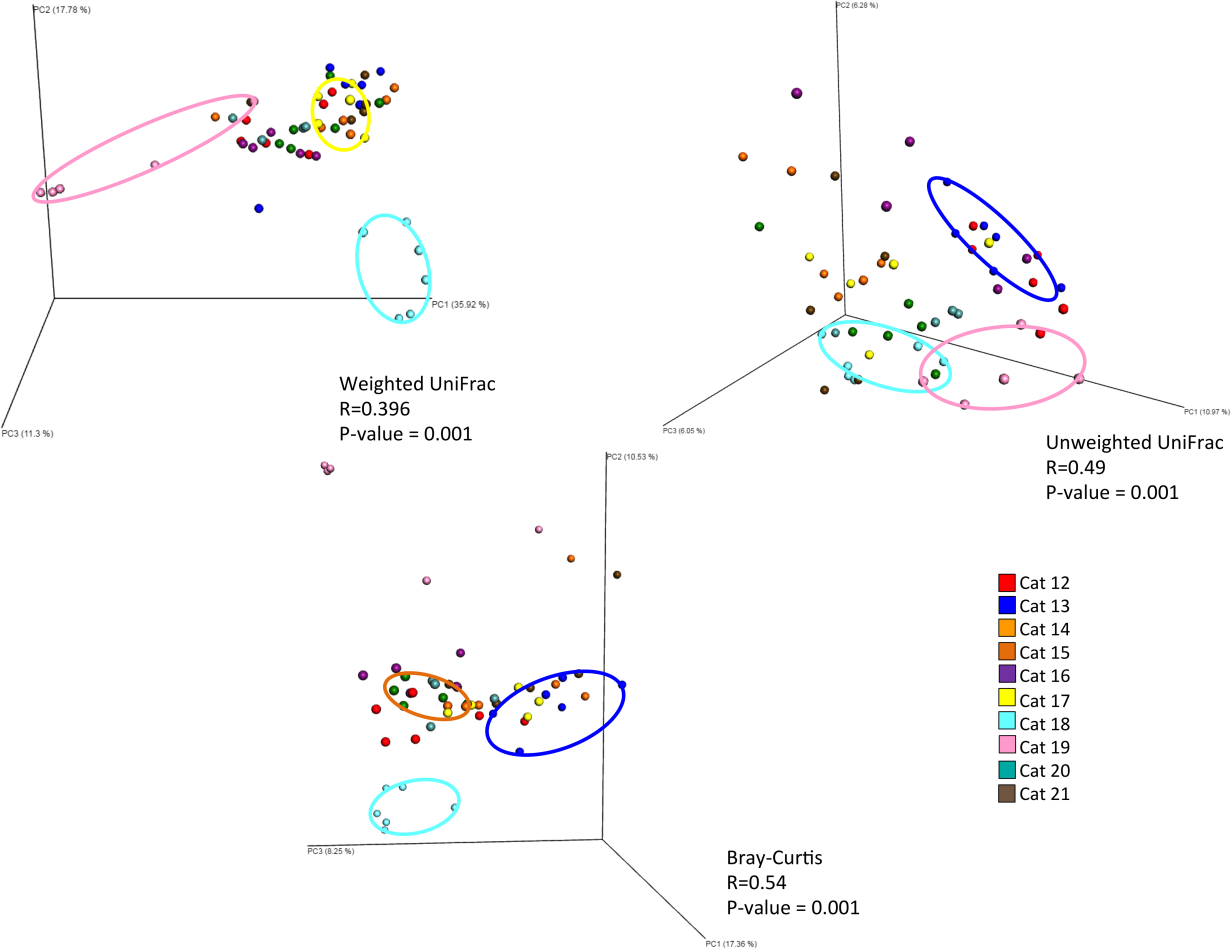
Principal coordinate analysis plots for allergic cat samples by individual. Principal coordinate analysis plots of weighted UniFrac, unweighted UniFrac, and Bray-Curtis distance matrices. Samples clustered strongly by individual cat with all 3 beta diversity metrics.

Community structure differed significantly between cats, according to the beta diversity metrics used (weighted UniFrac = 0.396, unweighted = 0.49, Bray-Curtis = 0.54, all with p = 0.001) (Fig 5). Community structure also differed by physiology of the sampled site (haired or mucosal) according to weighted UniFrac and Bray-Curtis (R = 0.230, R = 0.280 respectively, with p = 0.001). Unlike the samples from healthy cats, samples from allergic cats did not cluster by body site.

#### Microbial community composition

The most common phyla in allergic cats were found to be Proteobacteria (49.0%) followed by Firmicutes (21.5%), Actinobacteria (13.7%), Bacteroidetes (11.2%), and Fusobacteria (3.0%). The most abundant families were Pseudomonadaceae, Moraxellaceae, Pasteurellaceae, and Neisseriaceae. The haired sites sampled (axilla, ear canal, groin, interdigital, lumbar) had more Firmicutes, specifically Clostridiales. LEfSe revealed that the mucosal site sampled (nostril) had more *Corynebacterium* and that 5 of the cats had taxa communities that were very different than all of the other cats (S4 Fig).

### Skin microbiota of healthy versus allergic cats

The samples used in the analysis to compare healthy and allergic cats included swabs from the axilla, ear canal, groin, interdigital, lumbar, and nostril. The OTU table was rarefied to 2900. The average number of sequences per sample was 35850. A total of 7100 OTUs were identified resulting in 23 phyla.

#### Species richness and diversity

The results for alpha diversity are shown in Table 4. No significant differences were found in species richness and diversity between healthy and allergic cats overall. A significant difference was found between the allergic and healthy nostril with Chao1 (p = 0.02).

**Table 4:** Alpha diversity averages at 2900 sequences per sample for health status.

	**Chao1**	**Observed OTUs**	**Shannon**
Healthy	224 (137)	148 (101)	4.95 (1.45)
Allergic	217 (97)	137 (60)	4.60 (1.53)

Values represent averages with standard deviations in parenthesis.

#### Microbial community structure

A significant difference in overall community composition was not found between healthy and allergic cats. However, with the Bray-Curtis metric, the ear canal did show unique clustering between healthy and allergic skin (R = 0.223, p = 0.014) (S5 Fig).

#### Microbial community composition

**Fig 6:**
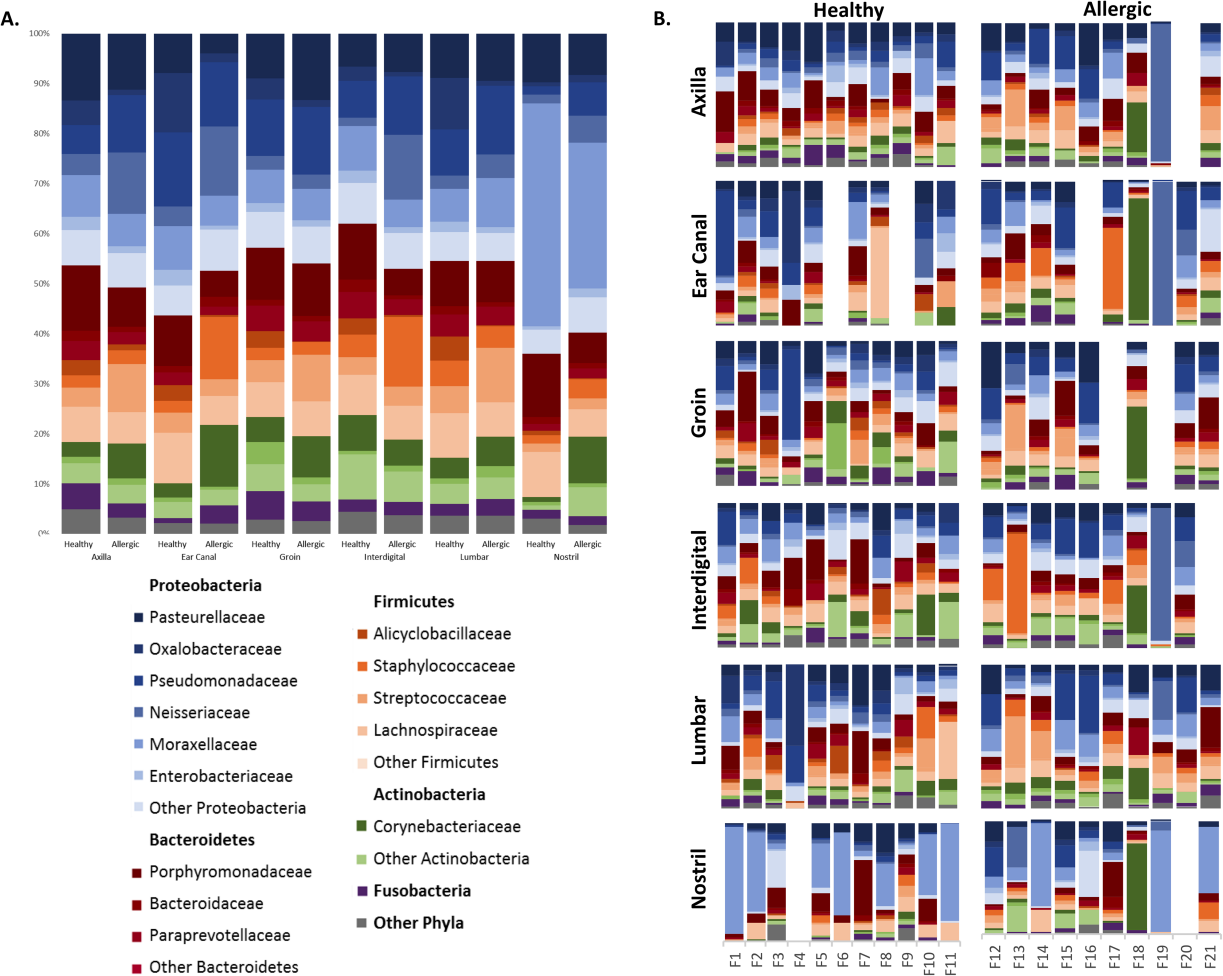
Relative taxa abundance at each body site sampled in healthy and allergic cats. A. Average relative taxa abundances at each body site (axilla, ear canal, groin, interdigital, lumbar, and nostril) sampled in healthy and allergic cats. B. Individual relative taxa abundances in every site sampled in healthy and allergic cats.

Fig 6. shows the relative abundances of the bacteria on the sites sampled from both healthy and allergic skin. Using Kruskal-Wallis tests, we discovered that many bacteria were differentially abundant on healthy and allergic skin. At the family level, healthy cats had more Oxalobacteraceae (p<0.0001), Alicyclobacillaceae (p<0.0001), Sphingobacteriaceae (p = 0.0023), and Chitinophagaceae (p = 0.0324), while allergic cats had more Bradyrhizobiaceae (p = 0.0080), Prevotellaceae (p = 0.0205), Vibrionaceae (p = 0.0310), and Halomonadaceae (p = 0.0023) (S4 Table).

**Fig 7:**
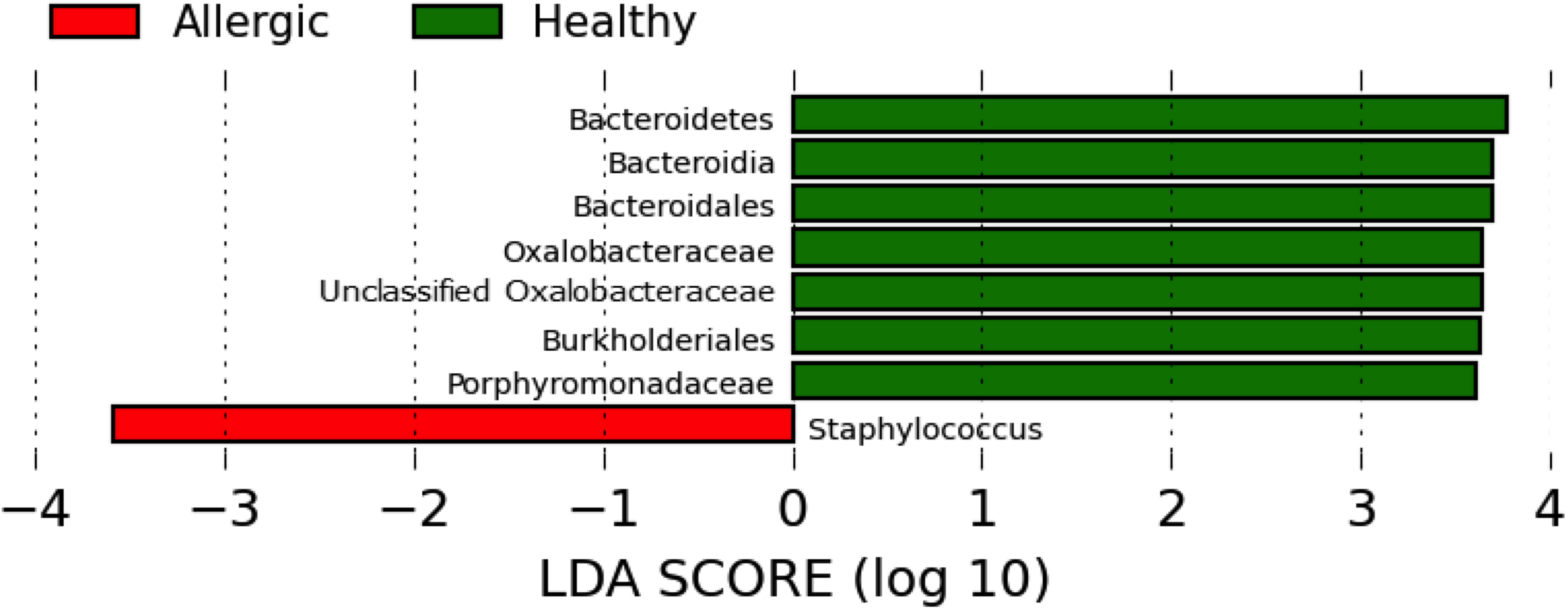
LEfSe plot of healthy vs allergic cat samples. Linear discriminant analysis effect size (LEfSe) plot of healthy vs allergic cat samples. Allergic cat samples had more *Staphylococcus* while samples from healthy cats had more Oxalobacteriaceae and Porphyromonadaceae.

LEfSe demonstrated that overall, healthy cats had more Oxalobacteraceae and Porphyromonadaceae, and allergic cats had more *Staphylococcus* (Fig 7). When looking at the different body sites separately, LEfSe also revealed differences in relative taxa abundance between healthy and allergic cats, including increased Oxalobacteraceae in healthy cats at multiple sites (S6 Fig).

## Discussion

In this study we demonstrated that the bacterial microbiota on the skin of healthy cats tends to prefer specific body site niches and skin physiologies (haired, mucosal, sebaceous, and oral). The bacteria colonizing the skin also varies between individual cats, however, less so than between different sites and types of regions sampled. In dogs, high variability was seen between individuals, but a similar microbiota was seen with respect to some of the body sites sampled [9].

We found the same phyla that were most abundant on the skin of dogs [9] were also the most abundant on feline skin, however in a different order (Proteobacteria, followed by Bacteroidetes, Firmicutes, and Actinobacteria). Bacteria of the phyla Bacteroidetes were highly abundant in all sites of the sampled cats, compared to what has been previously found in dogs [9]. This difference in Bacteroidetes abundance, a phyla typically associated with the oral cavity, may be due to the grooming behavior of cats.

When comparing specific body sites, ear canal, conjunctiva, nostril, and reproductive tracts had the least amount of observed OTUs and relatively low evenness. When considering the different physiologies of the sites sampled, haired and sebaceous sites were the most rich and diverse, followed by oral and mucosal. This is similar to what was found in dogs, where the haired sites had a higher species richness than mucosal surfaces and mucocutaneous junctions [9]. The diversity and richness was in the median range for canine ear canal, however in cats, the ear canal was one of the sites with relatively low diversity and richness.

Significant differences in the relative abundance of specific bacteria between healthy and allergic feline skin were identified (Fig 7). In the allergic cats, there were higher proportions of *Staphylococcus* which has been associated with atopic dermatitis in humans and dogs [19, 23, 46]. Although previous studies have indicated that *Staphylococcus* skin infections are less common in cats [47–50], possibly due to decreased adherence to corneocytes [33, 34], our finding indicates there may be a relationship between allergic skin disease and the increased abundance of *Staphylococcus* in cats.

Healthy and allergic cats also showed differences in community structure; samples from healthy cats clustered most by body site, whereas samples from allergic cats clustered most by individual cat. Additionally, the ear canal, a site often affected in hypersensitivity reactions [51], showed significant clustering between healthy and allergic cats. This shift may support the idea that there is a “normal” bacterial microbiota that is disrupted when an animal develops allergic skin disease. Instead of having a community unique to each site, with skin allergy flare ups, cats may become colonized all over the body by a single altered community. This change in the bacterial communities of all body sites, instead of just the affected area, may be reflective of the grooming behavior of cats. Every clinical presentation of a disease can be slightly different (e.g., different cutaneous reaction patterns), and similarly there does not seem to be one standard “unhealthy” microbiota.

Although this study may support the relationship between the bacterial microbiota and disease, the bacterial communities may not be the only ones affected by or contributing to disease. Studies on the fungal mycobiota have already looked for differences between animals with and without skin diseases. Meason-Smith et al. found that dogs most clustered by individual, regardless of health condition [21]. This same clustering by individual, regardless of health, was also seen in a similar study done on cats [20]. In both studies, differences in relative taxa abundance between healthy and allergic animals were identified. Overall, it seems that fungi on the skin of cats [20] and dogs [21] clusters mostly by individual animal, whereas bacteria seem to cluster mostly by body site [9].

One of the limitations of this study was the primer set used. It has previously been found that the primers we used to sequence the V4 region of the 16S rRNA are unable to amplify the genus *Propionibacterium* [52] due to a single mistmatch [53]. Although the primer set used in the presented study is not able to successfully amplify *Propionibacterium* spp., the primer set is able to amplify other bacteria, which cannot be amplified by other primer sets that have some relevance to the human skin microbiota [53]. However, studies comparing the effectiveness of different 16s rRNA gene primer sets in amplifying bacteria relevant to the feline skin microbiota have not yet been performed. This current study only identified very low abundances of the genus *Propionibacterium*, but future studies utilizing different primers may show the genus *Propionibacterium* to be more abundant than described in the current study and may reveal different relative abundances for other taxa.

Another limitation of this study was the small sample size used in the two groups and the heterogeneity of the feline population included in the study. We aimed to obtain as much homogeneity for certain factors in our sample cohort as possible by including approximately equal numbers of spayed females and neutered males. Most cats included in this study were domestic short hairs, the main breed in the general feline population. Obtaining homogeneous animal populations can be difficult, and doing so would limit the sample size included in this study even more. One way of overcoming heterogeneity of the sample cohort would be by including a homogenous feline research colony. Animals in research colonies are not exposed to typical household environments and often belong to a single breed through, so would not be representative of the general feline population, which was the goal of this study.

Currently, there is very little known about the feline skin bacterial microbiota [22]. Our study revealed that there are many more bacterial species found on feline skin than previously thought. It is important that at this point, results obtained with this first study be interpreted as descriptive and that additional follow up studies including larger sample sizes be repeated to support these findings. Furthermore, confounding factors, such as breed, sex and environment, that could influence the composition of the skin microbiota should be evaluated separately. Using other molecular techniques will allow for validation of these results and may reveal more about the microbiota than can be demonstrated with either next-generation sequencing or culture based methods. Evaluation of the changes in the microbiota with flares of disease and treatment may also help elucidate more about the relationship between cats and their microbiota.

## Supporting information

S1 Table Alpha diversity averages at 3100 sequences per sample for healthy cats. Values represent averages with standard deviations in parenthesis. Superscript letters represent sites that were significantly different according to Kruskal-Wallis tests with p<0.05.
(DOCX)

S2 Table Global R statistics for beta diversity analysis. (DOCX)

S3 Table R statistics for significant (R≥ 0.200, p≤0.05) pairwise comparisons. Shown R values are averages and p-values are ranges from pairwise comparisons. No significant pairwise comparisons in allergic samples with body site.
(DOCX)

S4 Table Filtered relative taxa abundances and results from Kruskal-Wallis tests for differential taxa abundance. P-values ≤ 0.05 are in bold. Median (Min-Max).
(DOCX)

S5 Table Hypersensitivity information (type, age of onset, seasonality, clinical signs, lesion distribution, ear problems, and treatments for ten allergic cats. Allergy treatments were concurrent. All cats with a Y in the steroids column had previously received steroids, except for F14 and F15 that were receiving steroids at the time of sampling. F18 was diagnosed with a ringworm infection, treated with limed dips, and lesions resolved three months prior to sample collection.
DSH-Domestic short hair, Per-Persian, Sia-Siamese, FBH-Flea bite hypersensitivity, FIHD-Food induced hypersensitivity, NFNFIHD- Non-flea non-food induced hypersensitivity, NFBH- non-flea bite hypersensitivity, G-gradual, Y-Yes, N-No. Modified from Meason-Smith C, Diesel A, Patterson AP, Older CE, Johnson TJ, Mansell JM, et al. Characterization of the cutaneous mycobiota in healthy and allergic cats using next generation sequencing. Vet Dermatol. 2016:1–11.
(DOCX)

S1 Fig Alpha diversity plots of the physiologies sampled in healthy cats. P-values are from Kruskal-Wallis tests. Lines indicate pairwise comparisons that resulted in significant p-values.
(TIF)

S2 Fig Principal coordinate analysis plots of Weighted UniFrac distance matrices of healthy cat samples by A. individual cat and B. physiology.
(TIF)

S3 Fig Average relative taxa abundances in the reproductive samples from healthy cats. (TIF)

S4 Fig LEfSe plots and cladograms from allergic cat samples. (TIF)

S5 Fig Principal coordinate analysis plot of healthy vs. allergic ear canal. Principal coordinate analysis plot based on Bray-Curtis distance matrix comparing healthy and allergic ear canal samples. Healthy and allergic ear canal samples showed distinct clustering.
(TIF)

S6 Fig LEfSe plots and cladograms comparing healthy and allergic samples at each body site. (TIF)

## References

[1] Grice EA, Kong HH, Renaud G, Young AC, Program NCS, Bouffard GG, et al A diversity profile of the human skin microbiota. Genome Res. 2008;18(7):1043–50. PubMed Central PMCID: PMC2493393. doi: 10.1101/gr.075549.107 18502944 10.1101/gr.075549.107 PMC2493393

[2] Grice EA, Segre JA. The skin microbiome. Nat Rev Microbiol. 2011;9(4):244–53. PubMed Central PMCID: PMC3535073. doi: 10.1038/nrmicro2537 21407241 10.1038/nrmicro2537 PMC3535073

[3] Li K, Bihan M, Yooseph S, Methe BA. Analyses of the microbial diversity across the human microbiome. PLoS One. 2012;7(6):e32118 PubMed Central PMCID: PMC3374608. doi: 10.1371/journal.pone.0032118 22719823 10.1371/journal.pone.0032118 PMC3374608

[4] Blekhman R, Goodrich JK, Huang K, Sun Q, Bukowski R, Bell JT, et al Host genetic variation impacts microbiome composition across human body sites. Genome Biol. 2015;16:191 PubMed Central PMCID: PMCPMC4570153. doi: 10.1186/s13059-015-0759-1 26374288 10.1186/s13059-015-0759-1 PMC4570153

[5] Zeeuwen PL, Ederveen TH, van der Krieken DA, Niehues H, Boekhorst J, Kezic S, et al Gram-positive anaerobe cocci are underrepresented in the microbiome of filaggrin-deficient human skin. J Allergy Clin Immunol. 2017;139(4):1368–71. doi: 10.1016/j.jaci.2016.09.017 27725187 10.1016/j.jaci.2016.09.017

[6] Lupp C, Robertson ML, Wickham ME, Sekirov I, Champion OL, Gaynor EC, et al Host-mediated inflammation disrupts the intestinal microbiota and promotes the Overgrowth of Enterobacteriaceae. Cell Host Microbe. 2007;2(2):119–29. doi: 10.1016/j.chom.2007.06.010 18005726 10.1016/j.chom.2007.06.010

[7] Salzman NH, Hung K, Haribhai D, Chu H, Karlsson-Sjoberg J, Amir E, et al Enteric defensins are essential regulators of intestinal microbial ecology. Nat Immunol. 2010;11(1):76–83. PubMed Central PMCID: PMCPMC2795796. doi: 10.1038/ni.1825 19855381 10.1038/ni.1825 PMC2795796

[8] Sanford JA, Gallo RL. Functions of the skin microbiota in health and disease. Semin Immunol. 2013;25(5):370–7. doi: 10.1016/j.smim.2013.09.005 24268438 10.1016/j.smim.2013.09.005 PMC4219649

[9] Rodrigues Hoffmann A, Patterson AP, Diesel A, Lawhon SD, Ly HJ, Elkins Stephenson C, et al The skin microbiome in healthy and allergic dogs. PLoS One. 2014;9(1):e83197 PubMed Central PMCID: PMC3885435. doi: 10.1371/journal.pone.0083197 24421875 10.1371/journal.pone.0083197 PMC3885435

[10] Rodrigues Hoffmann A, Proctor LM, Surette MG, Suchodolski JS. The Microbiome: The Trillions of Microorganisms That Maintain Health and Cause Disease in Humans and Companion Animals. Vet Pathol. 2016;53(1):10–21. doi: 10.1177/0300985815595517 26220947 10.1177/0300985815595517

[11] Hooda S, Minamoto Y, Suchodolski JS, Swanson KS. Current state of knowledge: the canine gastrointestinal microbiome. Anim Health Res Rev. 2012;13(1):78–88. doi: 10.1017/S1466252312000059 22647637 10.1017/S1466252312000059

[12] Minamoto Y, Hooda S, Swanson KS, Suchodolski JS. Feline gastrointestinal microbiota. Anim Health Res Rev. 2012;13(1):64–77. doi: 10.1017/S1466252312000060 22853923 10.1017/S1466252312000060

[13] Suchodolski JS. Companion animals symposium: microbes and gastrointestinal health of dogs and cats. J Anim Sci. 2011;89(5):1520–30. doi: 10.2527/jas.2010-3377 21075970 10.2527/jas.2010-3377 PMC7199667

[14] Misic AM, Davis MF, Tyldsley AS, Hodkinson BP, Tolomeo P, Hu B, et al The shared microbiota of humans and companion animals as evaluated from Staphylococcus carriage sites. Microbiome. 2015;3:2 PubMed Central PMCID: PMC4335418. doi: 10.1186/s40168-014-0052-7 25705378 10.1186/s40168-014-0052-7 PMC4335418

[15] Oh C, Lee K, Cheong Y, Lee SW, Park SY, Song CS, et al Comparison of the Oral Microbiomes of Canines and Their Owners Using Next-Generation Sequencing. PLoS One. 2015;10(7):e0131468 PubMed Central PMCID: PMC4489859. doi: 10.1371/journal.pone.0131468 26134411 10.1371/journal.pone.0131468 PMC4489859

[16] Sturgeon A, Pinder SL, Costa MC, Weese JS. Characterization of the oral microbiota of healthy cats using next-generation sequencing. Vet J. 2014;201(2):223–9. doi: 10.1016/j.tvjl.2014.01.024 24680670 10.1016/j.tvjl.2014.01.024

[17] Sturgeon A, Stull JW, Costa MC, Weese JS. Metagenomic analysis of the canine oral cavity as revealed by high-throughput pyrosequencing of the 16S rRNA gene. Vet Microbiol. 2013;162(2–4):891–8. doi: 10.1016/j.vetmic.2012.11.018 23228621 10.1016/j.vetmic.2012.11.018

[18] Yamasaki Y, Nomura R, Nakano K, Naka S, Matsumoto-Nakano M, Asai F, et al Distribution of periodontopathic bacterial species in dogs and their owners. Arch Oral Biol. 2012;57(9):1183–8. doi: 10.1016/j.archoralbio.2012.02.015 22417880 10.1016/j.archoralbio.2012.02.015

[19] Bradley CW, Morris DO, Rankin SC, Cain CL, Misic AM, Houser T, et al Longitudinal Evaluation of the Skin Microbiome and Association with Microenvironment and Treatment in Canine Atopic Dermatitis. J Invest Dermatol. 2016. 10.1016/j.jid.2016.01.023 PMC4877200 26854488

[20] Meason-Smith C, Diesel A, Patterson AP, Older CE, Johnson TJ, Mansell JM, et al Characterization of the cutaneous mycobiota in healthy and allergic cats using next generation sequencing. Vet Dermatol. 2017;28(1):71–e17. doi: 10.1111/vde.12373 27553477 10.1111/vde.12373

[21] Meason-Smith C, Diesel A, Patterson AP, Older CE, Mansell JM, Suchodolski JS, et al What is living on your dog's skin? Characterization of the canine cutaneous mycobiota and fungal dysbiosis in canine allergic dermatitis. FEMS Microbiol Ecol. 2015;91(12). PubMed Central PMCID: PMCPMC4657189. 10.1093/femsec/fiv139 PMC4657189 26542075

[22] Krogh HV KS. A study of skin diseases in dogs and cats. II. Microflora of the normal skin of dogs and cats. Nord Vet Med. 1976;28(9):459–63. 980697

[23] Fazakerley J, Nuttall T, Sales D, Schmidt V, Carter SD, Hart CA, et al Staphylococcal colonization of mucosal and lesional skin sites in atopic and healthy dogs. Veterinary Dermatology. 2009;20(3):179–84. doi: 10.1111/j.1365-3164.2009.00745.x 19392768 10.1111/j.1365-3164.2009.00745.x

[24] Furiani N, Scarampella F, Martino PA, Panzini I, Fabbri E, Ordeix L. Evaluation of the bacterial microflora of the conjunctival sac of healthy dogs and dogs with atopic dermatitis. Veterinary Dermatology. 2011;22(6):490–6. doi: 10.1111/j.1365-3164.2011.00979.x 21535255 10.1111/j.1365-3164.2011.00979.x

[25] Song SJ, Lauber C, Costello EK, Lozupone CA, Humphrey G, Berg-Lyons D, et al Cohabiting family members share microbiota with one another and with their dogs. Elife. 2013;2. 10.7554/eLife.00458 PMC3628085 23599893

[26] Havstad S, Wegienka G, Zoratti EM, Lynch SV, Boushey HA, Nicholas C, et al Effect of prenatal indoor pet exposure on the trajectory of total IgE levels in early childhood. J Allergy Clin Immunol. 2011;128(4):880–5 e4. PubMed Central PMCID: PMC3185205. doi: 10.1016/j.jaci.2011.06.039 21820714 10.1016/j.jaci.2011.06.039 PMC3185205

[27] Mandhane PJ, Sears MR, Poulton R, Greene JM, Lou WY, Taylor DR, et al Cats and dogs and the risk of atopy in childhood and adulthood. J Allergy Clin Immunol. 2009;124(4):745–50 e4. doi: 10.1016/j.jaci.2009.06.038 19703709 10.1016/j.jaci.2009.06.038

[28] Ownby DR, Johnson CC, Peterson EL. Exposure to dogs and cats in the first year of life and risk of allergic sensitization at 6 to 7 years of age. Jama-J Am Med Assoc. 2002;288(8):963–72. 10.1001/jama.288.8.963 12190366

[29] Bisgaard H, Simpson A, Palmer CNA, Bonnelykke K, Mclean I, Mukhopadhyay S, et al Gene-environment interaction in the onset of eczema in infancy: Filaggrin loss-of-function mutations enhanced by neonatal cat exposure. Plos Med. 2008;5(6). 10.1371/journal.pmed.0050131 PMC2504043 18578563

[30] Epstein TG, Bernstein DI, Levin L, Hershey GKK, Ryan PH, Reponen T, et al Opposing Effects of Cat and Dog Ownership and Allergic Sensitization on Eczema in an Atopic Birth Cohort. J Pediatr-Us. 2011;158(2):265–U128. 10.1016/j.jpeds.2010.07.026 PMC4910508 20884006

[31] Ownby DR, Johnson CC. Does Exposure to Cats or Dogs in Early Life Alter a Child's Risk of Atopic Dermatitis? J Pediatr-Us. 2011;158(2):184–6. 10.1016/j.jpeds.2010.09.055 PMC3052969 21074178

[32] Erwin EA, Wickens K, Custis NJ, Siebers R, Woodfolk J, Barry D, et al Cat and dust mite sensitivity and tolerance in relation to wheezing among children raised with high exposure to both allergens. J Allergy Clin Immun. 2005;115(1):74–9. doi: 10.1016/j.jaci.2004.10.030 15637550 10.1016/j.jaci.2004.10.030

[33] Lu YF, McEwan NA. Staphylococcal and micrococcal adherence to canine and feline corneocytes: quantification using a simple adhesion assay. Veterinary Dermatology. 2007;18(1):29–35. doi: 10.1111/j.1365-3164.2007.00567.x 17222237 10.1111/j.1365-3164.2007.00567.x

[34] Woolley KL, Kelly RF, Fazakerley J, Williams NJ, Nuttall TJ, McEwan NA. Reduced in vitro adherence of Staphylococcus species to feline corneocytes compared to canine and human corneocytes. Vet Dermatol. 2008;19(1):1–6. doi: 10.1111/j.1365-3164.2007.00649.x 18177284 10.1111/j.1365-3164.2007.00649.x

[35] Laulier M, Pradier E, Bigot Y, Periquet G. An Easy Method for Preserving Nucleic-Acids in-Field Samples for Later Molecular and Genetic-Studies without Refrigerating. J Evolution Biol. 1995;8(5):657–63.

[36] Caporaso JG, Lauber CL, Walters WA, Berg-Lyons D, Lozupone CA, Turnbaugh PJ, et al Global patterns of 16S rRNA diversity at a depth of millions of sequences per sample. Proc Natl Acad Sci U S A. 2011;108 Suppl 1:4516–22. PubMed Central PMCID: PMC3063599. 20534432 10.1073/pnas.1000080107 PMC3063599

[37] Caporaso JG, Kuczynski J, Stombaugh J, Bittinger K, Bushman FD, Costello EK, et al QIIME allows analysis of high-throughput community sequencing data. Nat Methods. 2010;7(5):335–6. PubMed Central PMCID: PMC3156573. doi: 10.1038/nmeth.f.303 20383131 10.1038/nmeth.f.303 PMC3156573

[38] Edgar RC. Search and clustering orders of magnitude faster than BLAST. Bioinformatics. 2010;26(19):2460–1. doi: 10.1093/bioinformatics/btq461 20709691 10.1093/bioinformatics/btq461

[39] DeSantis TZ, Hugenholtz P, Larsen N, Rojas M, Brodie EL, Keller K, et al Greengenes, a chimera-checked 16S rRNA gene database and workbench compatible with ARB. Appl Environ Microbiol. 2006;72(7):5069–72. PubMed Central PMCID: PMC1489311. doi: 10.1128/AEM.03006-05 16820507 10.1128/AEM.03006-05 PMC1489311

[40] Wang Q, Garrity GM, Tiedje JM, Cole JR. Naive Bayesian classifier for rapid assignment of rRNA sequences into the new bacterial taxonomy. Appl Environ Microbiol. 2007;73(16):5261–7. PubMed Central PMCID: PMC1950982. doi: 10.1128/AEM.00062-07 17586664 10.1128/AEM.00062-07 PMC1950982

[41] McDonald D, Price MN, Goodrich J, Nawrocki EP, DeSantis TZ, Probst A, et al An improved Greengenes taxonomy with explicit ranks for ecological and evolutionary analyses of bacteria and archaea. ISME J. 2012;6(3):610–8. PubMed Central PMCID: PMCPMC3280142. doi: 10.1038/ismej.2011.139 22134646 10.1038/ismej.2011.139 PMC3280142

[42] Caporaso JG, Bittinger K, Bushman FD, DeSantis TZ, Andersen GL, Knight R. PyNAST: a flexible tool for aligning sequences to a template alignment. Bioinformatics. 2010;26(2):266–7. PubMed Central PMCID: PMC2804299. doi: 10.1093/bioinformatics/btp636 19914921 10.1093/bioinformatics/btp636 PMC2804299

[43] Vazquez-Baeza Y, Pirrung M, Gonzalez A, Knight R. EMPeror: a tool for visualizing high-throughput microbial community data. Gigascience. 2013;2(1):16 PubMed Central PMCID: PMC4076506. doi: 10.1186/2047-217X-2-16 24280061 10.1186/2047-217X-2-16 PMC4076506

[44] Benjamini Y, Drai D, Elmer G, Kafkafi N, Golani I. Controlling the false discovery rate in behavior genetics research. Behav Brain Res. 2001;125(1–2):279–84. 11682119 10.1016/s0166-4328(01)00297-2

[45] Segata N, Izard J, Waldron L, Gevers D, Miropolsky L, Garrett WS, et al Metagenomic biomarker discovery and explanation. Genome Biol. 2011;12(6):R60 PubMed Central PMCID: PMC3218848. doi: 10.1186/gb-2011-12-6-r60 21702898 10.1186/gb-2011-12-6-r60 PMC3218848

[46] Leyden JJ, Marples RR, Kligman AM. Staphylococcus aureus in the lesions of atopic dermatitis. Br J Dermatol. 1974;90(5):525–30. 4601016 10.1111/j.1365-2133.1974.tb06447.x

[47] Loeffler A, Boag AK, Sung J, Lindsay JA, Guardabassi L, Dalsgaard A, et al Prevalence of methicillin-resistant Staphylococcus aureus among staff and pets in a small animal referral hospital in the UK. J Antimicrob Chemoth. 2005;56(4):692–7. 10.1093/jac/dki312 16141276

[48] Middleton JR, Fales WH, Luby CD, Oaks JL, Sanchez S, Kinyon JM, et al Surveillance of Staphylococcus aureus in veterinary teaching hospitals. J Clin Microbiol. 2005;43(6):2916–9. PubMed Central PMCID: PMCPMC1151956. doi: 10.1128/JCM.43.6.2916-2919.2005 15956418 10.1128/JCM.43.6.2916-2919.2005 PMC1151956

[49] O'Mahony R, Abbott Y, Leonard FC, Markey BK, Quinn PJ, Pollock PJ, et al Methicillin-resistant Staphylococcus aureus (MRSA) isolated from animals and veterinary personnel in Ireland. Vet Microbiol. 2005;109(3–4):285–96. doi: 10.1016/j.vetmic.2005.06.003 16026939 10.1016/j.vetmic.2005.06.003

[50] Rich M. Staphylococci in animals: prevalence, identification and antimicrobial susceptibility, with an emphasis on methicillin-resistant Staphylococcus aureus. Br J Biomed Sci. 2005;62(2):98–105. 15997888 10.1080/09674845.2005.11732694

[51] Miller WH, Griffin CE, Campbell KL. Muller & Kirk's Small Animal Dermatology. 7th ed. St. Louis: Elsevier; 2013. 390 p.

[52] Meisel JS, Hannigan GD, Tyldsley AS, SanMiguel AJ, Hodkinson BP, Zheng Q, et al Skin Microbiome Surveys Are Strongly Influenced by Experimental Design. J Invest Dermatol. 2016;136(5):947–56. PubMed Central PMCID: PMCPMC4842136. doi: 10.1016/j.jid.2016.01.016 26829039 10.1016/j.jid.2016.01.016 PMC4842136

[53] Zeeuwen PL, Boekhorst J, Ederveen TH, Kleerebezem M, Schalkwijk J, van Hijum SA, et al Reply to Meisel et al. J Invest Dermatol. 2016. 10.1016/j.jid.2016.11.013 27887953

