# Supplementary material for: The feline skin microbiota: The bacteria inhabiting the skin of healthy and allergic cats
Source: PLoS One. 2017 Jun 2;12(6):e0178555. doi: 10.1371/journal.pone.0178555 (PMC5456077; doi:10.1371/journal.pone.0178555)
Supplement: S1 Table — Values represent averages with standard deviations in parenthesis. Superscript letters represent sites that were significantly different according to Kruskal-Wallis tests with p<0.05. (DOCX) [file pone.0178555.s001.docx]

Table S1. Alpha diversity averages at 3100 sequences per sample for healthy cats

|  | Chao1 | Observed OTUs | Shannon |
| --- | --- | --- | --- |
| Body Site |  |  |  |
| Axilla | 226 (59) | 149 (48) | 5.53 (0.46) |
| Chin | 296 (163) | 201 (132) | 5.59 (1.13) |
| Conjunctiva | 133 (19)^C,A^ | 78 (12)^C,A^ | 3.66 (0.65)^A,C^ |
| Dorsal Nose | 282 (164)^CJ^ | 203 (104)^CJ^ | 6.00 (0.60)^CJ^ |
| Ear Canal | 155 (71)^C,A,DN^ | 103 (40)^DN,C^ | 4.30 (1.02)^DN,A,C^ |
| Groin | 272 (137)^CJ,EC^ | 187 (99)^CJ^ | 5.52 (1.10)^CJ,EC^ |
| Interdigital | 348 (222)^CJ,EC^ | 237 (174)^CJ,EC^ | 5.87 (1.22)^CJ,EC^ |
| Lumbar | 221 (74)^CJ^ | 134 (40)^CJ^ | 5.02 (1.05)^CJ,DN^ |
| Nostril | 152 (106)^ID,C,DN,A,G,L^ | 90 (56)^DN,C,G,I,A,L^ | 3.18 (1.66)^DN,A,I,C,G,L^ |
| Oral | 175 (26)^ID,CJ,A,DN,C^ | 129 (22)^CJ,N,DN^ | 4.78 (0.59)^DN,CJ,A,I,N,G^ |
| Pre-aural space | 471 (372)^O,CJ,EC,N,A,L^ | 319 (237)^CJ,N,O,EC,L,A^ | 6.39 (1.05)^CJ,O,N,EC,L,A^ |
| Reproductive (F) | 115 (44)_PAS,ID,A,C,DN,G,L,O_ | 72 (23)^DN,PAS,C,A,G,O,L,ID^ | 2.16 (1.43)^A,DN,PAS,C,I,G,L,O,EC^ |
| Reproductive (M) | 169 (48)^PAS,C^ | 94 (22)^DN,PAS,C,G,O,A,ID^ | 3.48 (0.89)^DN,PAS,A,I,C,G,O,L^ |

Values represent averages with standard deviations in parenthesis. Superscript letters represent sites that were significantly different according to Kruskal-Wallis tests with p<0.05.
