# Supplementary material for: The feline skin microbiota: The bacteria inhabiting the skin of healthy and allergic cats
Source: PLoS One. 2017 Jun 2;12(6):e0178555. doi: 10.1371/journal.pone.0178555 (PMC5456077; doi:10.1371/journal.pone.0178555)
Supplement: S2 Table — (DOCX) [file pone.0178555.s002.docx]

Table S2. Global R statistics for beta diversity analysis

|  |  | Weighted UniFrac | | Unweighted UniFrac | | Bray-Curtis | |
| --- | --- | --- | --- | --- | --- | --- | --- |
|  | **Factor** | **R** | **P-value** | **R** | **P-value** | **R** | **P-value** |
| Healthy | Cat | 0.175 | 0.001 | 0.195 | 0.001 | 0.209 | 0.001 |
|  | Body Site | 0.278 | 0.001 | 0.252 | 0.001 | 0.343 | 0.001 |
|  | Skin Physiology | 0.320 | 0.001 | 0.122 | 0.016 | 0.297 | 0.001 |
| Allergic | Cat | 0.396 | 0.001 | 0.490 | 0.001 | 0.540 | 0.001 |
|  | Body Site | -0.015 | 0.686 | -0.013 | 0.634 | -0.002 | 0.502 |
|  | Skin Physiology | 0.230 | 0.029 | 0.098 | 0.190 | 0.280 | 0.025 |
