# Supplementary material for: The feline skin microbiota: The bacteria inhabiting the skin of healthy and allergic cats
Source: PLoS One. 2017 Jun 2;12(6):e0178555. doi: 10.1371/journal.pone.0178555 (PMC5456077; doi:10.1371/journal.pone.0178555)
Supplement: S3 Table — Shown R values are averages and p-values are ranges from pairwise comparisons. No significant pairwise comparisons in allergic samples with body site. (DOCX) [file pone.0178555.s003.docx]

Table S3. R statistics for significant (R> 0.200, p<0.05) pairwise comparisons

|  |  | Weighted UniFrac | | | Unweighted UniFrac | | | Bray-Curtis | | | |
| --- | --- | --- | --- | --- | --- | --- | --- | --- | --- | --- | --- |
|  | **Factor** | **N** | **R** | **P-value** | **N** | **R** | **P-value** | **N** | **R** | **P-value** |  |
| Healthy | Cat | 8 | 0.263 | 0.010-0.030 | 10 | 0.261 | 0.009-0.017 | 8 | 0.264 | 0.004-0.006 |  |
|  | Skin Physiology | 4 | 0.387 | 0.006 | 3 | 0.316 | 0.004-0.006 | 2 | 0.422 | 0.006 |  |
|  | Body Site | 19 | 0.392 | 0.006-0.033 | 9 | 0.449 | 0.006-0.031 | 18 | 0.547 | 0.004-0.038 |  |
| Allergic | Cat | 14 | 0.560 | 0.016-0.047 | 14 | 0.496 | 0.009-0.034 | 20 | 0.479 | 0.011-0.005 |  |

Shown R values are averages and p-values are ranges from pairwise comparisons.

No significant pairwise comparisons in allergic samples with body site.
