# Supplementary material for: The feline skin microbiota: The bacteria inhabiting the skin of healthy and allergic cats
Source: PLoS One. 2017 Jun 2;12(6):e0178555. doi: 10.1371/journal.pone.0178555 (PMC5456077; doi:10.1371/journal.pone.0178555)
Supplement: S4 Table — P-values ≤ 0.05 are in bold. Median (Min-Max). (DOCX) [file pone.0178555.s004.docx]

Table S4. Filtered relative taxa abundances and results from Kruskal-Wallis tests for differential taxa abundance. P-values < 0.05 are in bold. Median (Min-Max).

|  | Axilla | | Ear Canal | | Groin | | Interdigital | | Lumbar | | Nostril | |  |  |  |
| --- | --- | --- | --- | --- | --- | --- | --- | --- | --- | --- | --- | --- | --- | --- | --- |
| Taxa | p-value | | Healthy | Allergic | Healthy | Allergic | Healthy | Allergic | Healthy | Allergic | Healthy | Allergic | Healthy | Allergic | |
| Acidobacteria | 0.9035 | | 0 (0-1.3) | 0 (0-0.3) | 0 (0-0) | 0 (0-1.3) | 0 (0-0.8) | 0 (0-1.8) | 0.1 (0-4.1) | 0 (0-0.9) | 0 (0-1.3) | 0 (0-1.7) | 0 (0-13.6) | 0 (0-5.1) | |
| [Chloracidobacteria] | 1.0752 | | 0 (0-1.2) | 0 (0-0.2) | 0 (0-0) | 0 (0-0.4) | 0 (0-0.4) | 0 (0-0.6) | 0.1 (0-4.1) | 0 (0-0.5) | 0 (0-1.3) | 0 (0-0) | 0 (0-13.6) | 0 (0-1.4) | |
| RB41 | 0.8113 | | 0 (0-0.7) | 0 (0-0.2) | 0 (0-1.2) | 0 (0-0.4) | 0 (0-0.4) | 0 (0-0.6) | 0.1 (0-4.1) | 0 (0-0.5) | 0 (0-1.3) | 0 (0-0.2) | 0 (0-13.6) | 0 (0-0.7) | |
| *Ellin6075* | 0.6948 | | 0 (0-0) | 0 (0-0.2) | 0 (0-0) | 0 (0-0) | 0 (0-0.4) | 0 (0-0.6) | 0 (0-4.1) | 0 (0-0.1) | 0 (0-1.1) | 0 (0-0) | 0 (0-7.2) | 0 (0-0.2) | |
| *Unclassified* | 0.6482 | | 0 (0-0) | 0 (0-0.2) | 0 (0-0) | 0 (0-0) | 0 (0-0.4) | 0 (0-0.6) | 0 (0-4.1) | 0 (0-0.1) | 0 (0-1.1) | 0 (0-0) | 0 (0-7.2) | 0 (0-0.2) | |
| Acidobacteria-6 | 0.8090 | | 0 (0-0.4) | 0 (0-0.3) | 0 (0-0) | 0 (0-0.3) | 0 (0-0.2) | 0 (0-1.2) | 0 (0-1.1) | 0 (0-0.4) | 0 (0-0.5) | 0 (0-1.7) | 0 (0-0.1) | 0 (0-1.7) | |
| iii1-15 | 0.8348 | | 0 (0-1.7) | 0 (0-0.3) | 0 (0-0) | 0 (0-0.3) | 0 (0-0.2) | 0 (0-1.2) | 0 (0-1.1) | 0 (0-0.4) | 0 (0-0.2) | 0 (0-0) | 0 (0-0.1) | 0 (0-1.7) | |
| *Unclassified* | 0.7991 | | 0 (0-0) | 0 (0-0.3) | 0 (0-0) | 0 (0-0.3) | 0 (0-0.2) | 0 (0-1.2) | 0 (0-1) | 0 (0-0.3) | 0 (0-0.2) | 0 (0-1.7) | 0 (0-0.1) | 0 (0-1.7) | |
| *Unclassified* | 0.7865 | | 0 (0-0) | 0 (0-0.3) | 0 (0-0) | 0 (0-0.3) | 0 (0-0.2) | 0 (0-1.2) | 0 (0-1) | 0 (0-0.3) | 0 (0-0.2) | 0 (0-1.7) | 0 (0-0.1) | 0 (0-1.7) | |
| Actinobacteria | 0.6868 | | 8.3 (2-14.8) | 9.8 (1.1-37.5) | 5.9 (0.1-13.8) | 10.1 (0.1-85.6) | 9.3 (2.1-51.9) | 7.8 (3.9-51.5) | 14.6 (4-32.6) | 9 (0.8-40.4) | 9.3 (0-17.9) | 12.1 (4.7-26.7) | 1.4 (0-10.9) | 5.9 (0.1-76.9) | |
| Actinobacteria | 0.7603 | | 7.9 (0-.9-14) | 9.2 (1.1-373.) | 5.9 (0.1-13.8) | 8.4 (0.1-85.4) | 7.9 (2.1-50.3) | 7.4 (3.3-50.6) | 14.5 (3.7-29.7) | 8.1 (0.8-40.4) | 9.3 (0-17.7) | 11.9 (3.9-26.7) | 1.3 (0-10) | 5.9 (0.1-76.7) | |
| Actinomycetales | 0.7093 | | 5.9 (0.4-76.7) | 9.2 (1.1-37.3) | 7.4 (0.9-14) | 7.3 (0.1-85.4) | 7.9 (2.1-49.9) | 6.9 (2.8-50.6) | 14.4 (3.7-29.7) | 8.1 (0.8-38.6) | 9.3 (0-17) | 0 (0-1.7) | 1.3 (0-9.7) | 5.9 (0.4-76.7) | |
| *Actinomycetaceae* | 0.4207 | | 1.6 (0-2.5) | 1.2 (0-3) | 0.1 (0-3.3) | 0.3 (0-2.2) | 0.8 (0-30.9) | 1.1 (0-3.1) | 0.5 (0-2.1) | 1.1 (0-3.1) | 1.2 (0-2.2) | 2.3 (0-3.6) | 0.5 (0-1.9) | 0.3 (0-4.7) | |
| *Actinomyces* | 0.4011 | | 1.3 (0-2.5) | 1.2 (0-2.8) | 0.1 (0-3.3) | 0.3 (0-2.2) | 0.8 (0-30.8) | 1.1 (0-3.1) | 0.4 (0-2.1) | 1.1 (0-3.1) | 1.2 (0-2.2) | 2.3 (0-3.3) | 0.5 (0-1.9) | 0.3 (0-4.7) | |
| *Brevibacteriaceae* | 0.7812 | | 0 (0-0) | 0 (0-0.6) | 0 (0-0.1) | 0 (0-0.1) | 0 (0-2) | 0 (0-0) | 0 (0-1.4) | 0 (0-0) | 0 (0-0.9) | 0 (0-1.4) | 0 (0-0) | 0 (0-0) | |
| *Brevibacterium* | 0.8643 | | 0 (0-0) | 0 (0-0.6) | 0 (0-0.1) | 0 (0-0.1) | 0 (0-2) | 0 (0-0) | 0 (0-1.4) | 0 (0-0) | 0 (0-0.9) | 0 (0-1.4) | 0 (0-0) | 0 (0-0) | |
| *Corynebacteriaceae* | 0.6807 | | 2.2 (0-7) | 3.2 (0.6-33.4) | 1.5 (0-13.8) | 4.6 (0-83.3) | 2.9 (0-15.3) | 2.7 (0.4-49.2) | 4.1 (0.6-27.8) | 1.9 (0.1-33) | 2.7 (0-10.2) | 4.4 (0.3-22.2) | 0.2 (0-5.7) | 0.4 (0-76) | |
| *Corynebacterium* | 0.6482 | | 2.2 (0-7) | 3.2 (0.6-33.4) | 1.5 (0-13.8) | 4.6 (0-83.3) | 2.9 (0-15.3) | 2.7 (0-49.2) | 3.1 (0.6-27.8) | 1.9 (0.1-33) | 2.7 (0-10.2) | 4.4 (0.3-22.2) | 0.2 (0-5.7) | 0.3 (0-76) | |
| *Dermabacteraceae* | 0.2382 | | 0 (0-0) | 0 (0-0.1) | 0 (0-6.2) | 0 (0-0.2) | 0 (0-2.3) | 0 (0-0.5) | 0.1 (0-2.3) | (0-0.1) | 0 (0-0.7) | 0 (0-0.9) | 0 (0-0) | 0 (0-0.8) | |
| *Brachybacterium* | 0.4905 | | 0 (0-0) | 0 (0-0.1) | 0 (0-6.2) | 0 (0-0.2) | 0 (0-2.3) | 0 (0-0.5) | 0.1 (0-2.3) | 0 (0-0.1) | 0 (0-0.7) | 0 (0-0.9) | 0 (0-0) | 0 (0-0.8) | |
| *Geodermatophilaceae* | 0.8653 | | 0 (0-1.1) | 0 (0-5.6) | 0 (0-0.5) | 0 (0-1.1) | 0 (0-0.9) | 0 (0-1.2) | 0 (0-0.6) | 0 (0-0.3) | 0 (0-0.4) | 0 (0-0.2) | 0 (0-0) | 0 (0-1.6) | |
| Modestobacter | 0.9199 | | 0 (0-0.2) | 0 (0-5.5) | 0 (0-0) | 0 (0-1.1) | 0 (0-0.9) | 0 (0-1.2) | 0 (0-0.2) | 0 (0-0.3) | 0 (0-0) | 0 (0-0.2) | 0 (0-0) | 0 (0-0) | |
| *Intrasporangiaceae* | 0.4069 | | 0 (0-2.4) | 0 (0-0.4) | 0 (0-0) | 0 (0-0.4) | 0.1 (0-3.2) | 0 (0-0.4) | 0 (0-2.4) | 0 (0-0.3) | 0 (0-0.7) | 0 (0-0.3) | 0 (0-0.2) | 0 (0-0.3) | |
| *Janibacter* | 0.7206 | | 0 (0-1.4) | 0 (0-0.3) | 0 (0-0) | 0 (0-0.4) | 0 (0-2.1) | 0 (0-0.4) | 0 (0-2.4) | 0 (0-0.1) | 0 (0-0.6) | 0 (0-0) | 0 (0-0.1) | 0 (0-0.2) | |
| *Microbacteriaceae* | 0.6935 | | 0 (0-2.8) | 0.2 (0-1.4) | 0 (0-0.7) | 0 (0-1.4) | 0.1 (0-1.4) | 0.1 (0-0.8) | 0.8 (0-2.6) | 0.3 (0-0.8) | 0 (0-0.6) | 0.3 (0-2.6) | 0 (0-0.5) | 0 (0-21.6) | |
| *Leucobacter* | 0.1958 | | 0 (0-1.3) | 0 (0-0.1) | 0 (0-0.7) | 0 (0-0) | 0 (0-0.9) | 0 (0-0.6) | 0.1 (0-1.3) | 0 (0-0.2) | 0 (0-0) | 0 (0-0.2) | 0 (0-0.3) | 0 (0-0) | |
| *Unclassified* | 0.0616 | | 0 (0-2.8) | 0 (0-1.2) | 0 (0-0.1) | 0 (0-0.5) | 0 (0-0.4) | 0.1 (0-0.3) | 0 (0-1.3) | 0.2 (0-0.7) | 0 (0-0.6) | 0.3 (0-2.6) | 0 (0-0.3) | 0 (0-21) | |
| *Micrococcaceae* | 0.2322 | | 0.4 (0-2.3) | 0.5 (0-0.9) | 0 (0-3.5) | 1 (0-2.7) | 0.8 (0-5.3) | 1 (0.1-1.9) | 0.6 (0-4.2) | 0.5 (0-3.1) | 0 (0-1.8) | 1.1 (0-4.5) | 0 (0-0.5) | 0.1 (0-2.3) | |
| *Microbispora* | 0.8531 | | 0 (0-1.5) | 0 (0-0) | 0 (0-0) | 0 (0-0.9) | 0 (0-0.1) | 0 (0-0) | 0 (0-0.1) | 0 (0-0) | 0 (0-0.7) | 0 (0-1.3) | 0 (0-0) | 0 (0-2.2) | |
| *Micrococcus* | 0.0477 | | 0 (0-2.3) | 0 (0-0.9) | 0 (0-1.8) | 0.4 (0-2.4) | 0 (0-1.1) | 0.6 (0-1.6) | 0 (0-2.4) | 0.2 (0-3.1) | 0 (0-1.3) | 0.5 (0-2.7) | 0 (0-0.2) | 0 (0-1.7) | |
| *Rothia* | 0.7448 | | 0 (0-0.3) | 0 (0-0.8) | 0 (0-0) | 0 (0-0) | 0 (0-1.5) | 0 (0-0.3) | 0 (0-1.4) | 0 (0-0.4) | (0-0.3) | 0.4 (0-1.8) | 0 (0-0.5) | 0 (0-0.1) | |
| *Unclassified* | 0.9382 | | 0 (0-0.4) | 0 (0-0.1) | 0 (0-0.6) | 0 (0-0.1) | 0 (0-2.7) | 0.1 (0-0.8) | 0 (0-1.6) | 0 (0-0.9) | 0 (0-1.8) | 0 (0-0.2) | 0 (0-0) | 0 (0-0) | |
| *Micromonosporaceae* | 0.8326 | | 0 (0-0) | 0 (0-0.2) | 0 (0-0) | 0 (0-0) | 0 (0-0.7) | 0 (0-0) | 0 (0-0.8) | 0 (0-5.3) | 0 (0-1.1) | 0 (0-1.1) | 0 (0-0.1) | 0 (0-4) | |
| *Unclassified* | 0.8191 | | 0 (0-0) | 0 (0-0.2) | 0 (0-0) | 0 (0-0) | 0 (0-0.4) | 0 (0-0) | 0 (0-0.5) | 0 (0-1.6) | 0 (0-0.5) | 0 (0-01) | 0 (0-0.1) | 0 (0-2.3) | |
| *Mycobacteriaceae* | 0.6083 | | 0 (0-0.3) | 0 (0-0.1) | 0 (0-0.3) | 0 (0-0.3) | 0 (0-1.2) | 0 (0-0.4) | 0 (0-1.2) | 0 (0-0.4) | 0 (0-2.7) | 0 (0-0.2) | 0 (0-0.1) | 0 (0-0.4) | |
| *Mycobacterium* | 0.5533 | | 0 (0-0.3) | 0 (0-0.1) | 0 (0-0.3) | 0 (0-0.3) | 0 (0-1.2) | 0 (0-0.4) | 0 (0-12) | 0 (0-0.4) | 0 (0-2.7) | 0 (0-0.2) | 0 (0-0.1) | 0 (0-0.4) | |
| *Nocardiaceae* | 0.7276 | | 0 (0-0.8) | 0 (0-0.2) | 0 (0-0.7) | 0 (0-0.4) | 0 (0-0.1) | 0 (0-0) | 0 (0-0.8) | 0 (0-1.2) | 0 (0-1.4) | 0 (0-0.9) | 0 (0-0) | 0 (0-0) | |
| *Rhodococcus* | 0.7120 | | 0 (0-0.8) | 0 (0-0.2) | 0 (0-0.7) | 0 (0-0.4) | 0 (0-0) | 0 (0-0) | 0 (0-.8) | 0 (0-1.2) | 0 (0-1.4) | 0 (0-0.9) | 0 (0-0) | 0 (0-0) | |
| *Nocardioidaceae* | 0.1820 | | 0.3 (0-2) | 0 (0-0.6) | 0 (0-1.9) | 0 (0-1) | 0.4 (0-1.3) | 0.1 (0-0.5) | 0.1 (0-3.5) | 0.1 (0-2.2) | 0.1 (0-0.8) | 0 (0-0.2) | 0 (0-0.7) | 0 (0-0) | |
| *Unclassified* | 0.1893 | | 0.1 (0-0.9) | 0 (0-0.2 | 0 (0-1.8) | 0 (0-0) | 0.3 (0-1.1) | 0 (0-0) | 0.1 (0-3) | 0.1 (0-1.1) | 0.1 (0-0.8) | 0 (0-0.2) | 0 (0-0.7) | 0 (0-0) | |
| *Pseudonocardiaceae* | 0.6018 | | 0 (0-0.3) | 0 (0-0.8) | 0 (0-2.4) | 0 (0-0.1) | 0 (0-0.8) | 0 (0-1.9) | 0 (0-1) | 0.2 (0-3) | 0 (0-0.7) | 0 (0-1.1) | 0 (0-0.1) | 0 (0-1) | |
| *Yaniellaceae* | 0.1109 | | 0 (0-0.6) | 0 (0-1.8) | 0 (0-0.6) | 0 (0-0) | 0 (0-1.5) | 0 (0-0.7) | 0 (0-4.6) | 0 (0-1.1) | 0 (0-0.4) | 0 (0-0) | 0 (0-0) | 0 (0-0) | |
| *Yaniella* | 0.1083 | | 0 (0-0.6) | 0 (0-1.8) | 0 (0-0.6) | 0 (0-0) | 0 (0-1.5) | 0 (0-0.7) | 0 (0-4.5) | 0 (0-1.1) | 0 (0-0.4) | 0 (0-0) | 0 (0-0) | 0 (0-0) | |
| *Unclassified* | 0.8571 | | 0 (0-1.7) | 0 (0-1.1) | 0 (0-0) | 0 (0-0.2) | 0 (0-0.9) | 0.1 (0-0.5) | 0 (0-1.1) | 0 (0-1.4) | 0 (0-0.9) | 0 (0-0.1) | 0 (0-1.2) | 0 (0-0) | |
| *Unclassified* | 0.9318 | | 0 (0-1.7) | 0 (0-1.1) | 0 (0-0) | 0 (0-0.2) | 0 (0-0.9) | 0.1 (0-0.5) | 0 (0-1.1) | 0 (0-1.4) | 0 (0-0.9) | 0 (0-0.1) | 0 (0-1.2) | 0 (0-0.1) | |
| Bifidobacteriales | 0.8607 | | 0 (0-11.3) | 0 (0-4) | 0 (0-1.4) | 0 (0-2.8) | 0 (0-0.6) | 0.2 (0-1) | 0 (0-1.4) | 0 (0-2.6) | 0 (0-3.7) | 0 (0-5.3) | 0 (0-1.6) | 0 (0-11.3) | |
| *Bifidobacteriaceae* | 0.8580 | | 0 (0-1.4) | 0 (0-4) | 0 (0-4.8) | 0 (0-2.8) | 0 (0-0.6) | 0.2 (0-1) | 0 (0-1.4) | 0 (0-2.6) | 0 (0-3.7) | 0 (0-5.3) | 0 (0-1.6) | 0 (0-11.3) | |
| *Bifidobacterium* | 0.8783 | | 0 (0-1.4) | 0 (0-3.5) | 0 (0-4.8) | 0 (0-2.8) | 0 (0-0.6) | 0 (0-1) | 0 (0-1.4) | 0 (0-2.6) | 0 (0-3.7) | 0 (0-5.3) | 0 (0-1.6) | 0 (0-11.3) | |
| Thermoleophilia | 1.0488 | | 0 (0-1.2) | 0.1  (0-0.7) | 0 (0-2.1) | 0 (0-2.6) | 0.4 (0-2.9) | 0 (0-2.1) | 0 (0-9.8) | 0.2 (0-2.8) | 0 (0-0.6) | 0 (0-0.1) | 0 (0-0) | 0 (0-2.2) | |
| Solirubrobacterales | 0.9333 | | 0 (0-2.2) | 0.1 (0-0.7) | 0 (0-1.2) | 0 (0-2.1) | 0.4 (0-2.9) | 0 (0-2.1) | 0 (0-8.8) | 0 (0-2.2) | 0 (0-0.6) | 0 (0-0.1) | 0 (0-0) | 0 (0-2.2) | |
| *Unclassified* | 0.5559 | | 0 (0-1) | 0 (0-0.2) | 0 (0-0.8) | 0 (0-1.4) | 0.3 (0-2.4) | 0 (0-0.8) | 0 (0-3.8) | 0 (0-1) | 0 (0-0) | 0 (0-0.1) | 0 (0-0) | 0 (0-0) | |
| *Unclassified* | 0.9169 | | 0 (0-1) | 0 (0-0.2) | 0 (0-0.8) | 0 (0-1.4) | 0.4 (0-2.4) | 0 (0-0.8) | 0 (0-3.8) | 0 (0-1) | 0 (0-0) | 0 (0-0.1) | 0 (0-0) | 0 (0-0) | |
| *Solirubrobacteraceae* | 0.9969 | | 0 (0-0.4) | 0 (0-0.2) | 0 (0-0) | 0 (0-0.8) | 0 (0-0.5) | 0 (0-2.1) | 0 (0-4.5) | 0 (0-1.2) | 0 (0-0.6) | 0 (0-0) | 0 (0-0) | 0 (0-0.7) | |
| *Unclassified* | 0.5351 | | 0 (0-0.4) | 0 (0-0.2) | 0 (0-0) | 0 (0-0.8) | 0 (0-0.4) | 0 (0-2.1) | 0 (0-4.5) | 0 (0-1.2) | 0 (0-0.6) | 0 (0-0) | 0(0-0) | 0 (0-0.7) | |
| Bacteroidetes | **0.0154** | | 16.3 (2.3-35.6) | 8.6 (1.1-22.9) | 13.4 (5.9-24.3) | 7.4 (0.2-20.4) | 13.9 (7-47.8) | 14.2 (4-31.2) | 14.5 (4.1-41.4) | 10.2 (1-15.5) | 15.7 (0-37.1) | 10.3 (4-31.9) | 12.8 (0-52.4) | 5 (0.1-42.2) | |
| [Saprospirae] | **0.0112** | | 0 (0-0.8) | 0 (0-0.1) | 0 (0-2.8) | 0 (0-0.9) | 0 (0-2) | 0 (0-0) | 0 (0-2) | 0 (0-1) | 0.3 (0-2.2) | 0 (0-1.3) | 0 (0-2.1) | 0 (0-1.3) | |
| [Saprospirales] | **0.0093** | | 0 (0-1.3) | 0 (0-0.1) | 0 (0-0.8) | 0 (0-0.9) | 0 (0-2) | 0 (0-0) | 0 (0-2) | 0 (0-1) | 0.3 (0-2.2) | 0 (0-1.3) | 0 (0-2.1) | 0 (0-1.3) | |
| *Chitinophagaceae* | **0.0324** | | 0 (0-0.8) | 0 (0-0.1) | 0 (0-2.8) | 0 (0-0.9) | 0 (0-2) | 0 (0-0) | 0 (0-2) | 0 (0-1) | 0.1 (0-2.2) | 0 (0-1.3) | 0 (0-2.1) | 0 (0-1.3) | |
| *Flavisolibacter* | 0.2284 | | 0 (0-0.8) | 0 (0-0) | 0 (0-0) | 0 (0-0.9) | 0 (0-1.8) | 0 (0-0) | 0 (0-0.4) | 0 (0-0.7) | 0 (0-1.6) | 0 (0-1.3) | 0 (0-2.1) | 0 (0-0) | |
| *Unclassified* | 0.0647 | | 0 (0-0.4) | 0 (0-0) | 0 (0-1.4) | 0 (0-0) | 0 (0-0.6) | 0 (0-0) | 0 (0-1.2) | 0 (0-0.4) | 0 (0-2.2) | 0 (0-0.6) | 0 (0-0.4) | 0 (0-0.2) | |
| Bacteroidia | 0.0542 | | 16 (2-32.9) | 8.5 (1.1-20.8) | 9.9 (5.9-23.8) | 6.7 (0.2-19.1) | 10.8 (4.4-45.4) | 12.3 (2.5-28.6) | 10.7 (2.1-38.6) | 8 (1-13.6) | 9.2 (0-36.3) | 9 (3.2-31.6) | 12.1 (0-51.8) | 3.6 (0.1-42) | |
| Bacteroidales | **0.0337** | | 3.6 (0.1-42) | 8.5 (1.1-20.8) | 16 (2-32.9) | 6.7 (0.2-19.1) | 10.8 (4.4-45.4) | 12.3 (2.5-28.6) | 10.7 (2.1-38.6) | 8 (1-13.6) | 9.2 (0-36.3) | 9 (3.2-31.6) | 12.1 (0-51.8) | 3.6 (0.1-42) | |
| *Unclassified* | 0.5357 | | 0 (0-1.2) | 0 (0-1) | 0 (0-0.3) | 0 (0-0) | 0 (0-0.2) | 0 (0-0.7) | 0 (0-0.4) | 0 (0-0.4) | 0 (0-0.7) | 0 (0-0.6) | 0 (0-1.1) | 0 (0-0.2) | |
| *Unclassified* | 0.4789 | | 0 (0-1.2) | 0 (0-1) | 0 (0-0.3) | 0 (0-0) | 0 (0-0.2) | 0 (0-0.7) | 0 (0-0.4) | 0 (0-0.4) | 0 (0-0.7) | 0 (0-0.6) | 0 (0-1.1) | 0 (0-0.2) | |
| *[Paraprevotellaceae]* | 0.0747 | | 2.4 (0-3.6) | 0.5 (0-3.3) | 0.6 (0-3.7) | 0.4 (0-10.3) | 0.7 (0-4.2) | 0.6 (0-2.4) | 1.3 (0-10.9) | 0.4 (0-2.5) | 1.4 (0-6.7) | 0.7 (0-2.4) | 0.9 (0-4.1) | 0.1 (0-9) | |
| *[Prevotella]* | 0.1247 | | 1.9 (0-3.6) | 0.5 (0-3.3) | 0.1 (0-2.9) | 0.4 (0-10.3) | 0.7 (0-4.2) | 0.6 (0-2.4) | 1.3 (0-10.9) | 0.4 (0-2.5) | 1.4 (0-6.7) | 0.7 (0-2.4) | 0.9 (0-4.1) | 0.1 (0-9) | |
| *Bacteroidaceae* | 0.5482 | | 1.5 (0-5.8) | 0.1 (0-2.1) | 1.5 (0-2) | 0 (0-3.7) | 1.2 (0-9.7) | 0.9 (0-3.4) | 0.7 (0-8.1) | 0.7 (0-3.7) | 1.1 (0-2.8) | 0.2 (0-3.1) | 0.1 (0-2.2) | 0.2 (0-2.7) | |
| *Bacteroides* | 0.5387 | | 1.5 (0-5.8) | 0.1 (0-2.1) | 1.5 (0-2) | 0 (0-3.7) | 1.2 (0-9.7) | 0.9 (0-3.4) | 0.7 (0-8.1) | 0.7 (0-3.7) | 1.1 (0-2.8) | 0.2 (0-3.1) | 0.1 (0-2.2) | 0.2 (0-2.7) | |
| *Porphyromonadaceae* | 0.0578 | | 12.5 (1.4-28.6) | 5.1 (0.8-15.9) | 6.9 (5-20) | 4.2 (0.1-18.5) | 8.1 (2.3034) | 9 (1.7-23.2) | 9.1 (0-31.7) | 5.4 (0.9-9.7) | 7.3 (0-29.6) | 6.9 (0.5-28) | 8.1 (0-47.8) | 2.9 (0-31.6) | |
| *Porphyromonas* | 0.0637 | | 12.5 (1.4-28.6) | 5.1 (0.7-15.7) | 6.4 (4.9-20) | 3.7 (0.1-18.4) | 8.1 (2.3-33.5) | 8.7 (1.7-23) | 9.1 (0-30.2) | 5.4 (0.9-9.4) | 7.3 (0-29.6) | 6.6 (0.5-27.4) | 8.1 (0-45.8) | 2.4 (0-30.6) | |
| *Prevotellaceae* | **0.0205** | | 0 (0-0.4) | 0.1 (0-2.3) | 0 (0-0.1) | 0.3 (0-1.6) | 0 (0-3.1) | 0.5 (0-2.4) | 0 (0-0.3) | 0 (0-0.8) | 0 (0-3.1) | 0.6 (0-1.4) | 0 (0-1.2) | 0 (0-2.2) | |
| *Prevotella* | **0.0224** | | 0 (0-0.4) | 0.1 (0-2.3) | 0 (0-0.1) | 0.3 (0-1.6) | 0 (0-3.1) | 0.5 (0-2.4) | 0 (0-0.3) | 0 (0-0.8) | 0 (0-3.1) | 0.6 (0-1.3) | 0 (0-1.2) | 0.1 (0-2.2) | |
| Cytophagia | 0.3543 | | 0.2 (0-2.4) | 0 (0-2) | 0 (0-0.6) | 0 (0-0.2) | 0.6 (0-2.7) | 0.1 (0-2.3) | 0.3 (0-4.3) | 0.9 (0-1.7) | 0 (0-5) | 0 (0-0.2) | 0 (0-0.1) | 0 (0-0.2) | |
| Cytophagales | 0.3444 | | 0 (0-0.2) | 0 (0-2) | 0.2 (0-2.4) | 0 (0-0.2) | 0.6 (0-2.7) | 0.1 (0-2.3) | 0.3 (0-4.3) | 0.9 (0-1.7) | 0 (0-5) | 0 (0-0.2) | 0 (0-0.1) | 0 (0-0.2) | |
| *Cytophagaceae* | 0.2735 | | 0.2 (0-2.4) | 0 (0-2) | 0 (0-0.6) | 0 (0-0.2) | 0.6 (0-2.5) | 0.1 (0-2.3) | 0.3 (0-4.3) | 0.9 (0-1.7) | 0 (0-5) | 0 (0-0.2) | 0 (0-0.1) | 0 (0-0.2) | |
| *Hymenobacter* | 0.1139 | | 0 (0-2.4) | 0 (0-0.2) | 0 (0-0.5) | 0 (0-0.2) | 0.2 (0-1.4) | 0 (0-0.7) | 0.2 (0-2.5) | 0.3 (0-1.2) | 0 (0-2) | 0 (0-0.1) | 0 (0-0.1) | 0 (0-0.2) | |
| Flavobacteriia | 1.0322 | | 0.4 (0.1-2.6) | 0.2 (0-5.6) | 0 (0-1.2) | 0.1 (0-1.3) | 0.3 (0-2.2) | 0.4 (0-3.8) | 0.4 (0-3.3) | 0.5 (0-3.5) | 0.1 (0-1.6) | 0.6 (0-14.9) | 0.2 (0-1) | 0.2 (0-2.2) | |
| Flavobacteriales | 0.8879 | | 0.2 (0-2.2) | 0.2 (0-5.6) | 0.4 (0.1-2.6) | 0.1 (0-1.3) | 0.3 (0-2.2) | 0.4 (0-3.8) | 0.4 (0-3.3) | 0.5 (0-3.5) | 0.1 (0-1.6) | 0.6 (0-14.9) | 0.2 (0-1) | 0.2 (0-2.2) | |
| *Flavobacteriaceae* | 0.8631 | | 0.4 (0.1-2.6) | 0.2 (0-5.6) | 0 (0-1.2) | 0.1 (0-1.3) | 0.3 (0-2.2) | 0.4 (0-3.8) | 0.4 (0-3.3) | 0.5 (0-3.5) | 0.1 (0-1.6) | 0.6 (0-14.3) | 0.2 (0-1) | 0.2 (0-2.2) | |
| *Capnocytophaga* | 0.6543 | | 0.3 (0-2.6) | 0.2 (0-4.2) | 0 (0-1) | 0 (0-1.3) | 0.1 (0-1.3) | 0.4 (0-2.6) | 0.1 (0-2.3) | 0.2 (0-1.8) | 0 (0-0.8) | 0.4 (0-8) | 0.1 (0-1) | 0 (0-2.1) | |
| *Unclassified* | 0.4299 | | 0 (0-0.6) | 0 (0-1.4) | 0 (0-1.2) | 0 (0-0.3) | 0.1 (0-1.7) | 0.1 (0-1.1) | 0 (0-0.9) | 0 (0-1.7) | 0 (0-1.1) | 0 (0-6.3) | 0 (0-0.2) | 0 (0-0.5) | |
| Sphingobacteriia | **0.0022** | | 0 (0-1.6) | 0 (0-0.1) | 0 (0-2.7) | 0 (0-0.4) | 0.2 (0-2.3) | 0 (0-0) | 0.4 (0-4) | 0 (0-0.1) | 0 (0-2.7) | 0 (0-0.7) | 0 (0-0.5) | 0 (0-2.8) | |
| Sphingobacteriales | **0.0006** | | 0 (0-2.8) | 0 (0-0.1) | 0 (0-1.6) | 0 (0-0.4) | 0.2 (0-2.3) | 0 (0-0) | 0.4 (0-4) | 0 (0-0.1) | 0 (0-2.7) | 0 (0-0.7) | 0 (0-0.5) | 0 (0-2.8) | |
| *Sphingobacteriaceae* | **0.0023** | | 0 (0-1.6) | 0 (0-0.1) | 0 (0-2.7) | 0 (0-0.4) | 0.1 (0-2.3) | 0 (0-0) | 0.4 (0-4) | 0 (0-0.1) | 0 (0-2.7) | 0 (0-0.7) | 0 (0-0.5) | 0 (0-2.8) | |
| *Pedobacter* | **0.0010** | | 0 (0-1.6) | 0 (0-0) | 0 (0-2.6) | 0 (0-0) | 0 (0-1.9) | 0 (0-0) | 0.2 (0-4) | 0 (0-0) | 0 (0-2.7) | 0 (0-0.7) | 0 (0-0.1) | 0 (0-1.2) | |
| *Unclassified* | 0.4075 | | 0 (0-0.1) | 0 (0-0) | 0 (0-0.1) | 0(0-0) | 0 (0-0.5) | 0 (0-0) | 0 (0-1.7) | 0 (0-0.1) | 0 (0-1) | 0 (0-0) | 0 (0-0) | 0 (0-1.6) | |
| Chlorobi | 0.8915 | | 0.1 (0-0.8) | 0 (0-1.5) | 0 (0-0.2) | 0 (0-0.2) | 0 (0-0.5) | 0.1 (0-1) | 0 (0-1.3) | 0 (0-0.4) | 0 (0-1.2) | 0 (0-0.8) | 0 (0-0.1) | 0 (0-0.3) | |
| OPB56 | 0.9065 | | 0.1 (0-0.8) | 0 (0-1.5) | 0 (0-0.2) | 0 (0-0.2) | 0 (0-0.5) | 0.1 (0-1) | 0 (0-1.3) | 0 (0-0.4) | 0 (0-1.2) | 0 (0-0.8) | 0 (0-0.1) | 0 (0-0.3) | |
| Unclassified | 0.8187 | | 0 (0-0.3) | 0 (0-1.5) | 0.1 (0-0.8) | 0 (0-0.2) | 0 (0-0.5) | 0.1 (0-1) | 0 (0-1.3) | 0 (0-0.4) | 0 (0-1.2) | 0 (0-0.8) | 0 (0-0.1) | 0 (0-0.3) | |
| *Unclassified* | 0.7737 | | 0.1 (0-0.8) | 0 (0-1.5) | 0 (0-0.2) | 0 (0-0.2) | 0 (0-0.5) | 0.1 (0-1) | 0 (0-1.3) | 0 (0-0.4) | 0 (0-1.2) | 0 (0-0.8) | 0 (0-0.1) | 0 (0-0.3) | |
| *Unclassified* | 0.7261 | | 0.1 (0-0.8) | 0 (0-1.5) | 0 (0-0.2) | 0 (0-0.2) | 0 (0-0.5) | 0.1 (0-1) | 0 (0-1.3) | 0 (0-04) | 0 (0-1.2) | 0 (0-0.8) | 0 (0-0.1) | 0 (0-0.3) | |
| Chloroflexi | 0.8866 | | 0 (0-1.6) | 0 (0-2) | 0 (0-0) | 0 (0-0.1) | 0 (0-1) | 0 (0-0.7) | 0 (0-0.4) | 0 (0-1.3) | 0 (0-0.7) | 0 (0-0.6) | 0 (0-0.1) | 0 (0-0.1) | |
| Firmicutes | 0.9746 | | 20.7 (10.2-26.4) | 16.9 (11.7-43.5) | 17.8(0-71) | 20.7 (0.3-61.7) | 17.8 (5.3-32.8) | 16.8 (11-42.6) | 20.8 (7.4-34.8) | 18.7 (2-73.3) | 25.2 (4.9-58.3) | 18.3 (9.1-56.9) | 14.7 (1.6-43.3) | 10.2 (0.2-20.2) | |
| Bacilli | 1.0257 | | 12.5 (0-20.7) | 12.2 (0.8-34.9) | 13.8 (0-68.7) | 16.6 (0.2-57.5) | 9.2 (2.9-30.8) | 7.9 (3-39.2) | 13 (0.4-30.6) | 12.5 (0.9-71.8) | 20.7 (0.6-45.3) | 11.4 (6.6-52.9) | 13.2 (1.4-26.5) | 6.9 (0.4-19.8) | |
| Bacillales | 0.7109 | | 2.6 (0.1-6) | 3 (0.2-15.9) | 6 (0-14.6) | 11.6 (0.2-56.8) | 7.2 (0.3-15.1) | 2.8 (0.8-7.7) | 11.2 (0.4-25.2) | 7.1 (0.5-70.2) | 14.6 (0.6-37.4) | 5.4 (0.8-13.6) | 1.2 (0-10.8) | 2.6 (0.1-6) | |
| *Alicyclobacillaceae* | **<0.0001** | | 4 (0-5.7) | 0.4 (0-4.9) | 2.4 (0-10.3) | 0 (0-1.7) | 2 (0.1-9.9) | 0 (0-0.9) | 1.1 (0-17.5) | 0 (0-1.8) | 2.6 (0-19) | 0 (0-1.2) | 0.4 (0-4.2) | 0.1 (0-0.5) | |
| *Alicyclobacillus* | **<0.0001** | | 4 (0-5.7) | 0.4 (0-4.9) | 2.4 (0-10.3) | 0 (0-1.7) | 2 (0.1-9.9) | 0 (0-0.9) | 1.1 (0-17.5) | 0 (0-1.8) | 2.6 (0-19) | 0 (0-1.2) | 0.4 (0-4.2) | 0.1 (0-0.5) | |
| *Bacillaceae* | 0.8753 | | 0.8 (0-4.4) | 0.3 (0-2) | 0 (0-58.9) | 1 (0-2.9) | 0.2 (0-3.5) | 0 (0-1.3) | 0.7 (0-2.5) | 0.6 (0-3.9) | 0.1 (0-29.2) | 0 (0-2.3) | 0.1 (0-0.7) | 0 (0-0.8) | |
| *Bacillus* | 0.9498 | | 0.1 (0-4.3) | 0.2 (0-1.3) | 0 (0-58.8) | 0.8 (0-2.2) | 0 (0-3.4) | 0 (0-0.7) | 0.1 (0-2.5) | 0.5 (0-3.4) | 0.1 (0-29.1) | 0 (0-2.3) | 0.1 (0-0.7) | 0 (0-0.2) | |
| *Geobacillus* | 0.5430 | | 0 (0-1.7) | 0 (0-1.5) | 0 (0-0) | 0 (0-2.1) | 0 (0-0.2) | 0 (0-0.6) | 0 (0-1) | 0 (0-0.4) | 0 (0-0.4) | 0 (0-0.9) | 0 (0-0) | 0 (0-0) | |
| *Paenibacillaceae* | 0.1762 | | 0 (0-0.2) | 0 (0-0) | 0 (0-0.2) | 0 (0-1.5) | 0 (0-0.6) | 0 (0-0.8) | 0 (0-1.6) | 0 (0-0.1) | 0 (0-1.1) | 0 (0-0) | 0 (0-0) | 0 (0-0) | |
| *Planococcaceae* | 0.9223 | | 0 (0-1.1) | 0 (0-2.3) | 0 (0-1.1) | 0.1 (0-2.4) | 0.1 (0-1.1) | 0 (0-1.3) | 0.2 (0-2.9) | 0.1 (0-3.3) | 0 (0-1.7) | 0 (0-0.2) | 0 (0-0) | 0 (0-0.2) | |
| *Sporosarcina* | 0.8153 | | 0 (0-1) | 0 (0-2.3) | 0 (0-1.1) | 0 (0-2.3) | 0 (0-1) | 0 (0-1.3) | 0 (0-2.9) | 0 (0-3.3) | 0 (0-0.7) | 0 (0-0) | 0 (0-0) | 0 (0-0.2) | |
| *Staphylococcaceae* | 0.1432 | | 2.3 (0-5.4) | 2.7 (0.2-7.9) | 0.8 (0-7.2) | 8.3 (0.1-55.2) | 2.1 (0-6.4) | 1.9 (0.8-5.7) | 2.4 (0-18.2) | 3.3 (0.3-68.3) | 2.9 (0-21.3) | 3.1 (0.1-11.1) | 0.1 (0-9.3) | 1.3 (0-6) | |
| *Jeotgalicoccus* | 0.4870 | | 0 (0-0.3) | 0 (0-0.2) | 0 (0-1.4) | 0 (0-0.1) | 0 (0-1.9) | 0 (0-0.6) | 0 (0-3.1) | 0 (0-4.5) | 0 (0-0.2) | 0 (0-0) | 0 (0-0.1) | 0 (0-1.1) | |
| *Staphylococcus* | 0.0892 | | 1.9 (0-5) | 2.7 (0-7.4) | 0.8 (0-6.8) | 8.3 (0.1-55.2) | 1.4 (0-6.4) | 1.7 (0.2-5.7) | 2.4 (0-17.5) | 3.3 (0.3-68.3) | 2.7 (0-21.3) | 3.1 (0.1-11.1) | 0.1 (0-9.3) | 1.3 (0-4.8) | |
| *Unclassified* | 0.7827 | | 0 (0-1.3) | 0 (0-0.4) | 0 (0-1.3) | 0 (0-0.8) | 0 (0-0.2) | 0 (0-0.9) | 0 (0-2.3) | 0.1 (0-0.3) | 0 (0-1.7) | 0 (0-1.2) | 0 (0-0.3) | 0 (0-0.2) | |
| *Unclassified* | 0.7495 | | 0 (0-1.3) | 0 (0-0.4) | 0 (0-1.3) | 0 (0-0.8) | 0 (0-0.2) | 0(0-0.9) | 0 (0-2.3) | 0.1 (0-0.3) | 0 (0-1.7) | 0 (0-1.2) | 0 (0-0.3) | 0 (0-0.2) | |
| Gemellales | 0.7657 | | 0 (0-0.5) | 0 (0-2) | 0.5 (0-2.7) | 0.1 (0-1.7) | 0.3 (0-1.8) | 0.1 (0-4) | 0 (0-0.7) | 0.2 (0-0.7) | 0 (0-4.7) | 0.9 (0-5.2) | 0.5 (0-7.9) | 0 (0-0.5) | |
| *Gemellaceae* | 0.6947 | | 0.5 (0-2.7) | 0 (0-2) | 0 (0-0.8) | 0.1 (0-1.7) | 0.3 (0-1.8) | 0.1 (0-4) | 0 (0-0.7) | 0.2 (0-0.7) | 0 (0-4.7) | 0.9 (0-5.2) | 0.5 (0-7.9) | 0 (0-0.5) | |
| *Unclassified* | 0.6566 | | 0.5 (0-2.7) | 0 (0-2) | 0 (0-0.8) | 0.1 (0-1.7) | 0.3 (0-1.8) | 0.1 (0-4) | 0 (0-0.7) | 0.2 (0-0.7) | 0 (0-4.7) | 0.8 (0-5.2) | 0.5 (0-7.9) | 0 (0-0.5) | |
| Lactobacillales | 0.6781 | | 2.3 (0-19.6) | 6.6 (0.5-26.8) | 3.4 (0-7.8) | 3.1 (0.1-6.2) | 4.2 (0-15.6) | 3.2 (1.5-33.3) | 2.7 (0-6.5) | 3.1 (0.3-8) | 2.7 (0-23.9) | 5.7 (2.9-34.8) | 5.8 (0.7-15.3) | 2.3 (0-19.6) | |
| *Aerococcaceae* | 1.0016 | | 0 (0-4.1) | 0 (0-1.3) | 0 (0-0.4) | 0 (0-0.6) | 0 (0-2.7) | 0 (0-1.4) | 0.2 (0-3.1) | 0.2 (0-1.1) | 0 (0-0.8) | 0 (0-1.2) | 1.9 (0-15.3) | 0 (0-19.1) | |
| *Alloiococcus* | 0.8366 | | 0 (0-0) | 0 (0-1.2) | 0 (0-0) | 0 (0-0) | 0 (0-0.2) | 0 (0-1.4) | 0 (0-3.1) | 0 (0-1.1) | 0 (0-0) | 0 (0-1.2) | 1.8 (0-15.3) | 0 (0-19.1) | |
| *Unclassified* | 0.2119 | | 0 (0-3.7) | 0 (0-0) | 0 (0-0) | 0 (0-0) | 0 (0-1.2) | 0 (0-0) | 0 (0-1.5) | 0 (0-0.3) | 0 (0-0.8) | 0 (0-0) | 0 (0-0) | 0 (0-0) | |
| *Carnobacteriaceae* | 0.7662 | | 0 (0-0.6) | 0 (0-0.8) | 0 (0-0.9) | 0 (0-0) | 0 (0-1.7) | 0 (0-2.4) | 0 (0-1.2) | 0 (0-1) | 0 (0-6.5) | 0 (0-1.1) | 0 (0-0) | 0 (0-0) | |
| *Enterococcaceae* | 0.3505 | | 0 (0-0.7) | 0.1 (0-1.7) | 0 (0-0.4) | 0 (0-0.9) | 0.1 (0-1.1) | 0.1 (0-1.1) | 0.2 (0-1.1) | 0.1 (0-3.8) | 0 (0-0.3) | 0 (0-3.7) | 0 (0-0.8) | (0-4.3) | |
| *Enterococcus* | 0.3240 | | 0 (0-0.7) | 0.1 (0-1.7) | 0 (0-0.4) | 0 (0-0.9) | 0.1 (0-1.1) | 0.1 (0-1.1) | 0.1 (0-1.1) | 0.1 (0-3.8) | 0 (0-0.3) | 0 (0-3.7) | 0 (0-0.8) | 0 (0-4.3) | |
| *Lactobacillaceae* | 0.5681 | | 0 (0-0.7) | 0.3 (0-4.6) | 0 (0-1.5) | 0 (0-1.3) | 0 (0-0.9) | 0 (0-0.4) | 0 (0-1) | 0 (0-0.4) | 0 (0-2.6) | 0 (0-0.1) | 0 (0-2.1) | 0 (0-0.1) | |
| *Lactobacillus* | 0.5553 | | 0 (0-0.7) | 0.3 (0-4.6) | 0 (0-1.5) | 0 (0-1.3) | 0 (0-0.9) | 0 (0-0.4) | 0 (0-1) | 0 (0-0.4) | 0 (0-2.6) | 0 (0-0.1) | 0 (0-2.1) | 0 (0-0.1) | |
| *Lactococcus* | 0.5626 | | 0 (0-0.5) | 0 (0-0.7) | 0 (0-17.7) | 0 (0-1.7) | 0 (0-0.5) | 0 (0-0.8) | 0 (0-0.1) | 0 (0-0.4) | 0 (0-6.2) | 0 (0-1.7) | 0 (0-0) | 0 (0-0.5) | |
| *Streptococcaceae* | 0.1513 | | 2.8 (0-7.2) | 5.7 (0-23.4) | 2 (0-17.8) | 1.6 (0-3.9) | 1.1 (0-15.6) | 2.9 (0.2-29.3) | 1.4 (0-4.3) | 1.8 (0.1-7.1) | 1.8 (0-20.9) | 5.3 (1.2-33.4) | 0.6 (0-7.9) | 0.5 (0-5.5) | |
| *Streptococcus* | 0.1191 | | 2.6 (0-7.2) | 5.7 (0-22.7) | 1.8 (0-4.7) | 1.6 (0-3.8) | 1.1 (0-15.6) | 2.9 (0.2-29.3) | 1.4 (0-4.2) | 1.8 (0.1-7.1) | 1.1 (0-20.9) | 5.3 (1.2-33.2) | 0.6 (0-7.9) | 0.3 (0-5.5) | |
| Clostridia | 1.0054 | | 6.9 (1.8-10.8) | 4.9 (0.4-8.5) | 2.2 (0-9.6) | 4 (0.1-7.3) | 4.5 (0.7-17.2) | 7.2 (3.3-15.3) | 6.2 (2.5-12.2) | 5.8 (1.1-12) | 5.3 (2.8-13) | 5.7 (0.9-15.2) | 0.7 (0-16.3) | 0.7 (0-2.9) | |
| Clostridiales | 0.9240 | | 0.9 (0-15.5) | 4.9 (0.4-8.5) | 6.9 (1.8-10.8) | 4 (0.1-7.3) | 4.5 (0.7-17.2) | 7.2 (3.3-15.3) | 6.2 (2.5-12.2) | 5.8 (1.1-12) | 5.3 (2.8-13) | 5.7 (0.9-15.2) | 0.7 (0-16.3) | 0.9 (0-15.5) | |
| *[Acidaminobacteraceae]* | 0.2306 | | 0 (0-2) | 0 (0-0.5) | 0 (0-0.3) | 0 (0-0.3) | 0 (0-0.8) | 0 (0-0.2) | 0.2 (0-3.9) | 0 (0-0.2) | 0 (0-0) | 0 (0-0.9) | 0 (0-0.7) | 0 (0-0.2) | |
| *Fusibacter* | 0.2289 | | 0 (0-2) | 0 (0-0.5) | 0 (0-0.3) | 0 (0-0.3) | 0 (0-0.8) | 0 (0-0.2) | 0.2 (0-3.9) | 0 (0-0.2) | 0 (0-0) | 0 (0-0.9) | 0 (0-0.7) | 0 (0-0.2) | |
| *[Tissierellaceae]* | 0.8602 | | 1.6 (0-5.6) | 0.7 (0-4.6) | 0.3 (0-2.1) | 0.7 (0-2.3) | 0.8 (0-7.3) | 0.3 (0-3.6) | 1.2 (0-2.3) | 0.9 (0-9.2) | 2 (0-7.3) | 0.9 (0-8.8) | 0.1 (0-11.1) | 0 (0-1.3) | |
| *Anaerococcus* | 0.9460 | | 0 (0-1.2) | 0 (0-1.2) | 0 (0-1.1) | 0 (0-0.1) | 0 (0-1) | 0 (0-0.5) | 0 (0-2.3) | 0 (0-2.3) | 0.2 (0-1.8) | 0.2 (0-2.2) | 0 (0-5) | 0 (0-0.7) | |
| *Finegoldia* | 0.8655 | | 0 (0-4.6) | 0 (0-1.9) | 0 (0-1.9) | 0 (0-1.6) | 0 (0-6.2) | 0 (0-0.1) | 0 (0-0.4) | 0 (0-4.7) | 0 (0-2.9) | 0 (0-1.7) | 0 (0-4) | 0 (0-0.3) | |
| *Helcococcus* | 0.1643 | | 0.1 (0-4.4) | 0 (0-0.5) | 0 (0-0.3) | 0 (0-2.3) | 0 (0-0.3) | 0 (0-0) | 0 (0-0.8) | 0 (0-0.2) | 0 (0-2.6) | 0 (0-1.1) | 0 (0-0.1) | 0 (0-0.1) | |
| *Parvimonas* | 0.6869 | | 0.2 (0-2.4) | 0 (0-1.7) | 0 (0-1.1) | 0 (0-0.8) | 0.1 (0-0.9) | 0.1 (0-3.6) | 0 (0-0.3) | 0.3 (0-0.9) | 0 (0-0.9) | 0 (0-1.5) | 0 (0-0.9) | 0 (0-0.6) | |
| *Peptoniphilus* | 0.2912 | | 0 (0-0.6) | 0 (0-0.9) | 0 (0-0) | 0 (0-0.3) | 0 (0-0) | 0 (0-0) | 0 (0-1.1) | 0 (0-1.3) | 0 (0-2.6) | 0.2 (0-1.9) | 0 (0-0) | 0 (0-0) | |
| *Clostridiaceae* | 0.5679 | | 0 (0-1) | 0 (0-0.4) | 0 (0-0.5) | 0.3 (0-2) | 0.1 (0-0.9) | 0.3 (0-1.4) | 0.1 (0-1.6) | 0.2 (0-3.1) | 0 (0-1.9) | 0.1 (0-1.7) | 0 (0-0.9) | 0 (0-0.5) | |
| *Clostridium* | 0.8580 | | 0 (0-0.1) | 0 (0-0) | 0 (0-0.5) | 0 (0-1.4) | 0.1 (0-0.8) | 0 (0-0.5) | 0 (0-1.5) | 0.1 (0-1.9) | 0 (0-0.6) | 0 (0-0.5) | 0 (0-0.4) | 0 (0-0.5) | |
| *Unclassified* | 0.5442 | | 0 (0-0.6) | 0 (0-0.4) | 0 (0-0) | 0 (0-0.6) | 0 (0-0.3) | 0 (0-1.4) | 0 (0-0.8) | 0.1 (0-1.1) | 0 (0-0.9) | 0 (0-1.7) | 0 (0-0.1) | 0 (0-0) | |
| *Lachnospiraceae* | 0.7347 | | 0.7 (0-2.6) | 0.2 (0-2.4) | 0 (0-1.1) | 0.6 (0-4.7) | 0.5 (0-3.2) | 1.9 (0-12.2) | 1.8 (0-5.8) | 0.8 (0-8) | 1.1 (0-4.2) | 1.4 (0-4.6) | 0 (0-1.6) | 0.2 (0-1.4) | |
| *Blautia* | 0.2136 | | 0 (0-0.2) | 0 (0-0.1) | 0 (0-0) | 0 (0-0.3) | 0 (0-0.1) | 0 (0-0.8) | 0 (0-1.1) | 0 (0-2.7) | 0 (0-1.8) | 0 (0-1.2) | 0 (0-0.2) | 0 (0-0.1) | |
| *Coprococcus* | 0.3741 | | 0.2 (0-0.4) | 0 (0-0.5) | 0 (0-0) | 0 (0-0.1) | 0 (0-0.7) | 0 (0-2.5) | 0 (0-0.9) | 0 (0-0.2) | 0 (0-2.1) | 0 (0-0.3) | 0 (0-0.6) | 0 (0-0.7) | |
| *Dorea* | 0.7260 | | 0 (0-0.9) | 0 (0-0.1) | 0 (0-0.1) | 0 (0-0.9) | 0 (0-2) | 0 (0-2.5) | 0 (0-0.3) | 0 (0-0) | 0 (0-4.2) | 0 (0-0.1) | 0 (0-0) | 0 (0-0.2) | |
| *Unclassified* | 0.4363 | | 0.5 (0-1.7) | 0.2 (0-2.4) | 0 (0-0.4) | 0.2 (0-3.3) | 0 (0-2.6) | 1.1 (0-6.4) | 0.7 (0-5.7) | 0.8 (0-4.3) | 0 (0-1.7) | 0.8 (0-2.1) | 0 (0-1) | 0 (0-0.9) | |
| *Peptococcaceae* | 0.7617 | | 0.3 (0-1.7) | 0 (0-3.9) | 0 (0-0) | 0 (0-1) | 0.3 (0-6.3) | 0 (0-2.6) | 0.2 (0-2.3) | 0 (0-1.1) | 0 (0-0.8) | 0.5 (0-2.7) | 0 (0-0.3) | 0 (0-0) | |
| *Peptococcus* | 0.7119 | | 0.3 (0-1.7) | 0 (0-3.9) | 0 (0-0) | 0 (0-1) | 0.3 (0-6.3) | 0 (0-2.6) | 0.1 (0-2.3) | 0 (0-1.1) | 0 (0-0.8) | 0.5 (0-2.7) | 0 (0-0.3) | 0 (0-0) | |
| *Peptostreptococcaceae* | 0.7306 | | 1.3 (0-2.2) | 0.2 (0-2.7) | 0 (0-7.9) | 0 (0-1.1) | 0.7 (0-2.4) | 0.3 (0-2.8) | 0.7 (0-3) | 0.1 (0-1.9) | 0 (0-4.2) | 0.4 (0-2.1) | 0.3 (0-1.8) | 0 (0-10.2) | |
| *Unclassified* | 0.6584 | | 0.6 (0-1.9) | 0 (0-2.7) | 0 (0-7.3) | 0 (0-1.1) | 0.2 (0-2) | 0.3 (0-1.8) | 0.7 (0-2.3) | 0.1 (0-1.8) | 0 (0-4.2) | 0.2 (0-2.1) | 0.3 (0-1.4) | 0 (0-0.3) | |
| *Ruminococcaceae* | 0.8504 | | 0 (0-1.7) | 0 (0-1) | 0 (0-0) | 0 (0-0.7) | 0 (0-0.6) | 0 (0-3.2) | 0.1 (0-2.7) | 0 (0-0.7) | 0 (0-1.2) | 0 (0-0.2) | 0 (0-0) | 0 (0-0.3) | |
| *Unclassified* | 0.6610 | | 0 (0-1.3) | 0 (0-0.2) | 0 (0-0) | 0 (0-0.7) | 0 (0-0.4) | 0 (0-2.5) | 0 (0-2.7) | 0 (0-0.1) | 0 (0-1.2) | 0 (0-0) | 0 (0-0) | 0 (0-0.3) | |
| *Veillonellaceae* | 0.1163 | | 0 (0-0.7) | 0 (0-0.6) | 0 (0-0) | 0 (0-1) | 0 (0-2.7) | 0.5 (0-1.2) | 0 (0-0.5) | 0 (0-0.4) | 0 (0-3.9) | 0.2 (0-1.5) | 0 (0-0.9) | 0 (0-0.3) | |
| *Veillonella* | 0.1593 | | 0 (0-0) | 0 (0-0.4) | 0 (0-0) | 0 (0-0) | 0 (0-2.4) | 0 (0-1.2) | 0 (0-0.2) | 0 (0-0.1) | 0 (0-3.8) | 0 (0-1.4) | 0 (0-0.8) | 0 (0-0.1) | |
| *Unclassified* | 0.5404 | | 0.3 (0-2.6) | 0 (0-1.6) | 0 (0-0.9) | 0 (0-0.6) | 0.2 (0-1.4) | 0.2 (0-0.3) | 0.3 (0-1.3) | 0.4 (0-1.3) | 0 (0-2.3) | 0 (0-0.6) | 0 (0-0.9) | 0 (0-0.2) | |
| *Unclassified* | 0.5206 | | 0.3 (0-2.6) | 0 (0-1.6) | 0 (0-0.9) | 0 (0-0.6) | 0.2 (0-1.4) | 0.2 (0-0.3) | 0.3 (0-1.3) | 0.4 (0-1.3) | 0 (0-2.4) | 0 (0-0.6) | 0 (0-0.9) | 0 (0-0.2) | |
| Erysipelotrichi | 0.9790 | | 0 (0-1.9) | 0 (0-1.3) | 0 (0-4.8) | 0.5 (0-3.1) | 0.4 (0-2.5) | 0.8 (0-3.4) | 0.8 (0-5) | 0.3 (0-1.9) | 0.4 (0-1.8) | 0.2 (0-2.8) | 0.2 (0-2.2) | 0 (0-4) | |
| Erysipelotrichales | 0.9623 | | 0 (0-4) | 0 (0-1.3) | 0 (0-1.9) | 0.5 (0-3.1) | 0.4 (0-2.5) | 0.8 (0-3.4) | 0.8 (0-5) | 0.3 (0-1.9) | 0.4 (0-1.8) | 0.2 (0-2.8) | 0.2 (0-2.2) | 0 (0-4) | |
| *Erysipelotrichaceae* | 0.9589 | | 0 (0-1.9) | 0 (0-1.3) | 0 (0-4.8) | 0.5 (0-3.1) | 0.4 (0-2.5) | 0 (0-3.4) | 0.8 (0-5) | 0.3 (0-1.9) | 0.4 (0-1.8) | 0.2 (0-2.8) | 0.2 (0-2.2) | 0 (0-4) | |
| *p-75-a5* | 0.7950 | | 0 (0-0.8) | 0 (0-1.3) | 0 (0-0.2) | 0 (0-0.4) | 0.1 (0-0.4) | 0 (0-0.9) | 0 (0-0.9) | 0 (0-0.2) | 0 (0-1.4) | 0 (0-1.2) | 0 (0-0.8) | 0 (0-1.8) | |
| *Unclassified* | 0.3371 | | 0 (0-1.6) | 0 (0-0.9) | 0 (0-4.88) | 0 (0-2.2) | 0.1 (0-2.2) | 0.1 (0-3) | 0.4 (0-4.1) | 0 (0-1.7) | 0.3 (0-0.9) | 0.1 (0-1.7) | 0.2 (0-1.6) | 0 (0-4) | |
| Fusobacteria | 0.9093 | | 4.9 (0.8-13.9) | 3.3 (0-6.2) | 0.6 (0-4.3) | 2.8 (0-10.6) | 4.2 (0-11.3) | 3.9 (0-10.1) | 21. (0-9.5) | 2.7 (0-5.6) | 1.3 (0-6.4) | 3.2 (0-7.6) | 1.4 (0-4.9) | 0.6 (0-6.6) | |
| Fusobacteriia | 0.9926 | | 4.9 (0.8-13.9) | 3.3 (0-6.2) | 0.6 (0-4.3) | 2.8 (0-10.6) | 4.2 (0-11.3) | 3.9 (0-10.1) | 2.1 (0-9.5) | 2.7 (0-5.6) | 1.3 (0-6.4) | 3.2 (0-7.6) | 1.4 (0-4.9) | 0.6 (0-6.6) | |
| Fusobacteriales | 0.8349 | | 0.6 (0-6.6) | 3.3 (0-6.2) | 4.9 (0.8-13.9) | 2.8 (0-10.6) | 4.2 (0-11.3) | 3.9 (0-10.1) | 2.1 (0-9.5) | 2.7 (0-5.6) | 1.3 (0-6.4) | 3.2 (0-7.6) | 1.4 (0-4.9) | 0.6 (0-6.6) | |
| *Fusobacteriaceae* | 0.8841 | | 3.9 (0.8-9.2) | 2.8 (0-4.6) | 0.3 (0-4) | 2.8 (0-10) | 3.5 (0-11.3) | 3 (0-6.7) | 1.6 (0-8.6) | 2.4 (0-5.3) | 1.3 (0-5.2) | 2 (0-6.4) | 1.1 (0-4.9) | 0.3 (0-5.8) | |
| *Fusobacterium* | 0.8671 | | 3.9 (0-9.2) | 2.8 (0-4.6) | 0.3 (0-4) | 2.3 (0-9.7) | 3.5 (0-11.3) | 3 (0-6) | 1.6 (0-8.6) | 2.3 (0-5.3) | 1.3 (0-5.2) | 1.8 (0-6.4) | 1.1 (0-4.9) | 0.5 (0-5.8) | |
| *Leptotrichiaceae* | 0.9906 | | 0.4 (0-4.7) | 0 (0-1.6) | 0 (0-0.4) | 0 (0-1.8) | 0.5 (0-8.7) | 0 (0-4.4) | 0 (0-0.9) | 0 (0-2.5) | 0 (0-4.2) | 0.8 (0-2.1) | 0 (0-2) | 0 (0-2.2) | |
| *Leptotrichia* | 0.1814 | | 0 (0-0.4) | 0 (0-1.6) | 0 (0-0.4) | 0 (0-1.8) | 0 (0-0.5) | 0 (0-4.4) | 0 (0-0.1) | 0 (0-1) | 0 (0-0.2) | 0 (0-2.1) | 0 (0-2) | 0 (0-2.1) | |
| *Unclassified* | 0.1969 | | 0.2 (0-4.6) | 0 (0-0.2) | 0 (0-0.3) | 0 (0-1) | 0 (0-8.6) | 0 (0-3.4) | 0 (0-0.9) | 0 (0-1.5) | 0 (0-4.2) | 0 (0-2) | 0 (0-0.6) | 0 (0-2.2) | |
| Gemmatimonadetes | 0.6521 | | 0 (0-2.9) | 0 (0-0.6) | 0 (0-0.2) | 0 (0-0.8) | 0 (0-1.2) | 0 (0-0.4) | 0 (0-0.7) | 0 (0-1.7) | 0 (0-1) | 0 (0-0) | 0 (0-0) | 0 (0-0.6) | |
| Proteobacteria | 0.9935 | | 45.7 (33.7-61) | 45.8 (20.6-96.1) | 58.4 (18.5-81.6) | 49 (4.8-99.3) | 40.8 (20.6-78.5) | 44.7 (25.9-74) | 38.1 (24.5-55.9) | 45.8 (14.2-96.2) | 43.4 (21.3-95) | 45.5 (20.9-70.1) | 68.6 (25.8-94.3) | 59.8 (7.5-98.2) | |
| Alphaproteobacteria | 1.0458 | | 2.4 (0-17.4) | 3.2 (0.1-9.2) | 3.8 (0-11.8) | 3.3 (0-10.7) | 3.7 (0.7-11.3) | 4.3 (0.9-8) | 3.4 (0.6-20.8) | 4.3 (0.9-9) | 1.5 (0-20.1) | 2.3 (0.4-7.3) | 0.2 (0-32.3) | 0.4 (0.1-20.3) | |
| Caulobacterales | 0.7428 | | 0 (0-2.7) | 0 (0-0.6) | 0 (0-0.6) | 0 (0-0.7) | 0 (0-3.2) | 0.1 (0-2.3) | 0 (0-1.4) | 0 (0-0.6) | 0 (0-0.3) | 0 (0-1.3) | 0 (0-0.6) | 0 (0-2.7) | |
| *Caulobacteraceae* | 0.6812 | | 0 (0-0.1) | 0 (0-0.6) | 0 (0-0.8) | 0 (0-0.6) | 0 (0-3.2) | 0.1 (0-2.3) | 0 (0-1.2) | 0 (0-0.6) | 0 (0-0.3) | 0 (0-1.3) | 0 (0-0.6) | 0 (0-1.7) | |
| Rhizobiales | 0.0735 | | 0.2 (0-9.7) | 2 (0-2.5) | 0.9 (0-2.6) | 1.6 (0-8.9) | 0.6 (0.3-3.6) | 1.8 (0.1-4.3) | 0.8 (0-8) | 1.4 (0.1-2.3) | 0.5 (0-5.8) | 0.7 (0-5.1) | 0 (0-1.2) | 0.2 (0-9.7) | |
| *Bradyrhizobiaceae* | **0.0080** | | 0 (0-1.9) | 0.1 (0-2.4) | 0 (0-0.1) | 0.6 (0-7.3) | 0 (0-0.9) | 0.7 (0-4.3) | 0.1 (0-1.5) | 0 (0-1.6) | 0 (0-0.7) | 0 (0-5.1) | 0 (0-0.3) | 0.1 (0-5.8) | |
| *Balneimonas* | 0.8714 | | 0 (0-1.4) | 0 (0-0.3) | 0 (0-0) | 0 (0-0.2) | 0 (0-0.6) | 0 (0-0.6) | 0 (0-1.2) | 0 (0-0) | 0 (0-0.7) | 0 (0-1) | 0 (0-0) | 0 (0-2.1) | |
| *Unclassified* | **0.0012** | | 0 (0-0.6) | 0 (0-2.4) | 0 (0-0.1) | 0.6 (0-7.2) | 0 (0-0.2) | 0.5 (0-4.1) | 0 (0-0.5) | 0 (0-1.5) | 0 (0-0.2) | 0 (0-3.7) | 0 (0-0) | 0 (0-5.6) | |
| *Hyphomicrobiaceae* | 0.7233 | | 0 (0-1.8) | 0 (0-1.5) | 0 (0-1.1) | 0 (0-0.8) | 0.1 (0-0.9) | 0 (0-0.7) | 0 (0-1.8) | 0.1 (0-1.1) | 0 (0-2.3) | 0 (0-0.6) | 0 (0-0.4) | 0 (0-1.7) | |
| *Rhodoplanes* | 0.8192 | | 0 (0-0.8) | 0 (0-0.2) | 0 (0-0) | 0 (0-0.8) | 0 (0-0.1) | 0 (0-0) | 0 (0-0.2) | 0 (0-1.1) | 0 (0-1.5) | 0 (0-0.6) | 0 (0-0) | 0 (0-1.6) | |
| *Methylobacteriaceae* | 0.6026 | | 0 (0-0.7) | 0 (0-1.7) | 0 (0-0.2) | 0 (0-0) | 0.3 (0-1.7) | 0.1 (0-1) | 0.1 (0-2.4) | 0.3 (0-1) | 0 (0-2.3) | 0 (0-0.5) | 0 (0-0.5) | 0 (0-2.5) | |
| *Methylobacterium* | 0.7915 | | 0 (0-0.7) | 0 (0-1.7) | 0 (0-0.2) | 0 (0-0) | 0 (0-1.7) | 0 (0-1) | 0 (0-2) | 0 (0-0.3) | 0 (0-2.3) | 0 (0-0.5) | 0 (0-0.1) | 0 (0-2.5) | |
| *Rhizobiaceae* | 0.8751 | | 0 (0-1) | 0 (0-1.1) | 0 (0-4) | 0 (0-2) | 0 (0-0.6) | 0 (0-0.7) | 0.1 (0-1.8) | 0 (0-1.1) | 0 (0-2.1) | 0 (0-0.4) | 0 (0-0) | 0 (0-2.1) | |
| *Agrobacterium* | 0.9599 | | 0 (0-1) | 0 (0-0.8) | 0 (0-4) | 0 (0-0.9) | 0 (0-0.6) | 0 (0-0.7) | 0.1 (0-1.5) | 0 (0-1.1) | 0 (0-2.1) | 0 (0-0.4) | 0 (0-0) | 0 (0-1.9) | |
| Rhodobacterales | 0.7946 | | 0 (0-1.9) | 0.3 (0-1.5) | 0 (0-3.1) | 0 (0-1.1) | 0.5 (0-1.8) | 0 (0-2.1) | 0.2 (0-2.8) | 0.4 (0-1.1) | 0 (0-1.2) | 0.1 (0-1.2) | 0 (0-1.2) | 0 (0-1.9) | |
| *Rhodobacteraceae* | 0.7323 | | 0 (0-3.1) | 0.3 (0-1.5) | 0 (0-0.8) | 0 (0-1.1) | 0.5 (0-1.8) | 0 (0-2.1) | 0.2 (0-2.8) | 0.4 (0-1.1) | 0 (0-1.2) | 0.1 (0-1.2) | 0 (0-1.2) | 0 (0-1.9) | |
| *Paracoccus* | 0.9686 | | 0 (0-1.3) | 0 (0-0.2) | 0 (0-0) | 0 (0-0.8) | 0 (0-0) | 0 (0-0.4) | 0 (0-1.4) | 0 (0-0.7) | 0 (0-0.3) | 0 (0-0.2) | 0 (0-1.2) | 0 (0-0.1) | |
| *Unclassified* | 0.2776 | | 0 (0-0.3) | 0.3 (0-1.1) | 0 (0-0.8) | 0 (0-0.5) | 0.1 (0-1.7) | 0 (0-2.1) | 0 (0-1.2) | 0.1 (0-1) | 0 (0-0.4) | 0 (0-0.9) | 0 (0-0.1) | 0 (0-1.6) | |
| Rhodospirillales | 0.9404 | | 0 (0-0.4) | 0 (0-0.3) | 0 (0-1.5) | 0 (0-0.7) | 0.1 (0-1.1) | 0 (0-3) | 0.2 (0-2.2) | 0 (0-1.3) | 0 (0-0) | 0 (0-0.3) | 0 (0-25) | 0 (0-0.4) | |
| *Acetobacteraceae* | 1.0028 | | 0 (0-0.9) | 0 (0-0.3) | 0 (0-1.4) | 0 (0-0.7) | 0.1 (0-0.5) | 0 (0-2.1) | 0.2 (0-1.6) | 0 (0-0.8) | 0 (0-0) | 0 (0-0.1) | 0 (0-25) | 0 (0-0.2) | |
| *Unclassified* | 0.7583 | | 0 (0-0.9) | 0 (0-0.3) | 0 (0-1.4) | 0 (0-0.2) | 0.1 (0-0.4) | 0 (0-2.1) | 0 (0-0.3) | 0 (0-0.8) | 0 (0-0) | 0 (0-0.1) | 0 (0-20.7) | 0(0-0.2) | |
| Rickettsiales | 0.3369 | | 0 (0-0.1) | 0 (0-0.3) | 0 (0-2.7) | 0 (0-1.2) | 0 (0-1.5) | 0 (0-0.7) | 0.2 (0-1.5) | 0 (0-1.6) | 0 (0-2.2) | 0 (0-0.4) | 0 (0-0.5) | 0 (0-0.1) | |
| Sphingomonadales | 0.7171 | | 0.2 (0-7.5) | 0.9 (0-4.9) | 2.1 (0-12.5) | 1.1 (0-2.4) | 1.4 (0-5.7) | 1.2 (0-3) | 1.2 (0.2-8.4) | 1.5 (0-2.7) | 1 (0-10.8) | 0.5 (0-4) | 0.1 (0-6.8) | 0.2 (0-7.5) | |
| *Sphingomonadaceae* | 0.5618 | | 2.1 (0-12.2) | 0.9 (0-4.8) | 1.6 (0-11.7) | 1.1 (0-2.4) | 1.4 (0-5.6) | 1.2 (0-3) | 1.2 (0.2-8.3) | 0.8 (0-2.7) | 1 (0-10.8) | 0.4 (0-4) | 0.1 (0-2.9) | 0.2 (0-7.5) | |
| *Kaistobacter* | 0.1021 | | 0 (0-0.6) | 0 (0-0.7) | 0 (0-0.7) | 0 (0-1) | 0.3 (0-1.2) | 0 (0-2.2) | 0.1 (0-3.7) | 0 (0-0.8) | 0 (0-4.5) | 0 (0-0.1) | 0 (0-1.5) | 0 (0-0.2) | |
| *Sphingobium* | 0.4136 | | 0 (0-7.3) | 0.4 (0-2.9) | 0.4 0-6.4) | 0 (0-1.1) | 0 (0-1.5) | 0 (0-1.7) | 0 (0-2.3) | 0.1 (0-0.3) | 0 (0-1.5) | 0.1 (0-1.6) | 0 (0-0.7) | 0.1 (0-5.6) | |
| *Sphingomonas* | 0.7357 | | 0.1 (0-1.7) | 0.2 (0-0.8) | 0 (0-1.3) | 0.1 (0-1.4) | 0.3 (0-2.2) | 0.1 (0-0.9) | 0.4 (0-4.4) | 0.2 (0-1.9) | 0 (0-2.9) | 0.4 (0-3.5) | 0 (0-0.2) | 0.2 (0-15.2) | |
| *Unclassified* | 0.1404 | | 0.7 (0-4.7) | 0 (0-1.1) | 0 (0-10.9) | 0 (0-0.3) | 0.2 (0-2.5) | 0 (0-1.3) | 0.3 (0-2.7) | 0.1 (0-1.1) | 0 (0-5.1) | 0 (0-1.7) | 0 (0-1.9) | 0 (0-0.2) | |
| Betaproteobacteria | **0.0264** | | 9 (4-17.7) | 3.9 (1.2-94.6) | 8.3 (2.2-49.7) | 5.3 (0.5-98.4) | 6.5 (3.7-12.7) | 4.1 (1.2-15) | 4 (2.5-13.3) | 2.7 (1.2-91.1) | 8.2 (3.5-56.6) | 4.1 (2.1-26.8) | 2.7 (0-5.5) | 2.9 (0.1-35.4) | |
| Burkholderiales | **<0.0001** | | 0.4 (0-6) | 0.8 (0-4) | 3.2 (0.6-17) | 1.8 (0-5.9) | 4.3 (1.3-10.3) | 1.3 (0.1-4.7) | 2.5 (0.6-13.3) | 1.6 (0-2.8) | 5.1 (1.3-56.5) | 0.9 (0.1-3.7) | 0.7 (0-2.8) | 0.4 (0-6) | |
| *Alcaligenaceae* | 0.1227 | | 0 (0-0) | 0 (0-0) | 0 (0-0.4) | 0 (0-2.3) | 0 (0-1.1) | 0 (0-1.1) | 0 (0-0.3) | 0 (0-2.3) | 0 (0-0.5) | 0 (0-0.3) | 0 (0-0) | 0 (0-0.7) | |
| *Sutterella* | 0.0605 | | 0 (0-0) | 0 (0-0) | 0 (0-0) | 0 (0-2.3) | 0 (0-0.4) | 0 (0-1.1) | 0 (0-0.1) | 0 (0-2.3) | 0 (0-0) | 0 (0-0.3) | 0 (0-0) | 0 (0-0.7) | |
| *Oxalobacteraceae* | **<0.0001** | | 3.1 (0.5-16.9) | 0.8 (0-4) | 5.2 (1.6-49.1) | 1 (0-5.9) | 3.7 (1-10.2) | 0.5 (0-4.6) | 2.1 (0.4-13.2) | 1.2 (0-2) | 3.7 (1.3-56.4) | 0.4 (0-3.5) | 0.6 (0-2.8) | 0.3 (0-6) | |
| *Janthinobacterium* | 0.7379 | | 0 (0-0) | 0 (0-0) | 0 (0-1.3) | 0 (0-1.1) | 0 (0-0.7) | 0 (0-1.6) | 0 (0-0.1) | 0 (0-0) | 0 (0-1) | 0 (0-0.6) | 0 (0-0.1) | 0 (0-0.3) | |
| *Unclassified* | **<0.0001** | | 3.1 (0.5-16.8) | 0.8 (0-4) | 4.7 (1.6-48.6) | 1 (0-4.7) | 3.6 (1-10) | 0.5 (0-4.2) | 2 (0.4-13.2) | 1.2 (0-1.9) | 3.7 (1.3-55.9) | 0.4 (0-3.5) | 0.6 (0-2.8) | 0.2 (0-15.2) | |
| Neisseriales | 0.9183 | | 1 (0-35) | 2.7 (0-94.6) | 4 (0.7-10.4) | 1.6 (0-98.4) | 2.4 (0-6.3) | 1.8 (0-14.4) | 1.4 (0-3.6) | 0.9 (0.1-91.1) | 3 (0-7.8) | 2.2 (0.1-26.3) | 1.7 (0-4.7) | 1 (0-35) | |
| *Neisseriaceae* | 0.1000 | | 4 (0.7-10.4) | 2.7 (0-94.6) | 0.3 (0-25.9) | 1.6 (0-98.4) | 2.4 (0-6.3) | 1.8 (0-14.4) | 1.4 (0-3.6) | 0.9 (0.1-01.1) | 3 (0-7.8) | 2.2 (0.1-26.3) | 1.7 (0-4.7) | 1.2 (0-35) | |
| *Kingella* | 0.7619 | | 0.3 (0-4.4) | 0 (0-1.8) | 0 (0-1.1) | 0 (0-2.9) | 0.2 (0-2) | 0.1 (0-1.6) | 0.1 (0-2.3) | 0.1 (0-3.2) | 0.1 (0-1.9) | 0.1 (0-2.4) | 0 (0-1) | 0 (0-22.6) | |
| *Unclassified* | 0.7393 | | 3.8 (0.5-6.1) | 2.1 (0-94.6) | 0.1 (0-25.1) | 0.5 (0-98.4) | 1.9 (0-6.2) | 1.2 (0-13.1) | 1.2 (0-3.1) | 0.8 (0.1-90.9) | 1.9 (0-7.5) | 1.2 (0.1-26.2) | 1.1 (0-4.4) | 0 (0-12.1) | |
| Deltapropetobacteria | 0.8219 | | 0(0-0.6) | 0 (0-0.7) | 0 (0-1) | 0 (0-2.2) | 0 (0-1.3) | 0 (0-0.7) | 0.3 (0-5.2) | 0.8 (0-1.6) | 0 (0-0.1) | 0 (0-0.2) | 0 (0-1) | 0 (0-23.9) | |
| Myxococcales | 0.7268 | | 1 (0-23.1) | 0 (0-0.3) | 0 (0-0.6) | 0 (0-2.2) | 0 (0-0.2) | 0 (0-0.7) | 0 (0-4.7) | 0 (0-0.8) | 0 (0-0) | 0 (0-0) | 0 (0-0.1) | 0 (0-23.1) | |
| Epsilonproteobacteria | 0.4571 | | 1.1 (0-7) | 0.5 (0-4.3) | 0.2 (0-10.9) | 0 (0-21.8) | 0.5 (0-5.4) | 0.3 (0-1.7) | 0.5 (0-1.7) | 0 (0-2.2) | 0 (0-0.4) | 0 (0-1.6) | 0 (0-1.2) | 0 (0-0.4) | |
| Campylobacterales | 0.5879 | | 0 (0-0.4) | 0.5 (0-4.3) | 1.1 (0-7) | 0 (0-21.8) | 0.5 (0-5.4) | 0.3 (0-1.7) | 0.5 (0-1.7) | 0 (0-2.2) | 0 (0-0.4) | 0 (0-1.6) | 0 (0-1.2) | 0 (0-0.4) | |
| *Campylobacteraceae* | 0.1207 | | 0.5 (0-4.4) | 0 (0-1.1) | 0.2 (0-10.9) | 0 (0-0.5) | 0.3 (0-4.2) | 0 (0-0.7) | 0 (0-1.4) | 0 (0-1.1) | 0 (0-0) | 0 (0-0.7) | 0 (0-1.2) | 0 (0-0.3) | |
| *Arcobacter* | 0.7018 | | 0 (0-2.2) | 0 (0-0.3) | 0 (0-10.9) | 0 (0-0) | 0 (0-1.4) | 0 (0-0.6) | 0 (0-0.9) | 0 (0-0.5) | 0 (0-0) | 0 (0-0.5) | 0 (0-0.6) | 0 (0-0.1) | |
| *Campylobacter* | 0.2162 | | 0.5 (0-3.5) | 0 (0-1.1) | 0 (0-2.4) | 0 (0-0.5) | 0.1 (0-3.3) | 0 (0-0.7) | 0 (0-1.1) | 0 (0-0.9) | 0 (0-0) | 0 (0-0.2) | 0 (0-0.6) | 0 (0-0) | |
| *Helicobacteraceae* | 0.7875 | | 0.2 (0-3.6) | 0 (0-4) | 0 (0-0.2) | 0 (0-21.4) | 0 (0-1.2) | 0 (0-1) | 0.1 (0-1.2) | 0 (0-1) | 0 (0-0.4) | 0 (0-1.6) | 0 (0-0) | 0 (0-0.1) | |
| *Flexispira* | 0.7201 | | 0 (0-3.3) | 0 (0-3.8) | 0 (0-0.2) | 0 (0-19.7) | 0 (0-1.2) | 0 (0-0.9) | 0 (0-1.2) | 0 (0-0.9) | 0 (0-0) | 0 (0-0.4) | 0 (0-0) | 0 (0-0.3) | |
| *Wolinella* | 0.3984 | | 0 (0-0.5) | 0 (0-1) | 0 (0-0) | 0 (0-0.7) | 0 (0-0) | 0 (0-1) | 0 (0-0.4) | 0 (0-0.1) | 0 (0-0.4) | 0 (0-1.3) | 0 (0-0) | 0 (0-0.1) | |
| Gammaproteobacteria | 1.0773 | | 31 (16.5-40.2) | 35.9 (1.3-61.9) | 32.1 (11.9-70.6) | 21.9 (0.9-47) | 29.8 (6.6-70.8) | 32.8 (10.1-63) | 23.2 (10.8-50.2) | 32.3 (3.9-43.7) | 32.6 (8.4-44.8) | 28.7 (11.4-60.4) | 64 (17.1-92.2) | 31.5 (0.1-89) | |
| Aeromonadales | 0.7204 | | 0 (0-0.3) | 0 (0-0.4) | 0 (0-0.3) | 0 (0-0.9) | 0 (0-1) | 0 (0-1.1) | 0 (0-0.5) | 0 (0-0.3) | 0 (0-1.6) | 0 (0-1.1) | 0 (0-0.1) | 0 (0-0.3) | |
| *Aeromonadaceae* | 0.3633 | | 0 (0-0.3) | 0 (0-0.4) | 0 (0-0) | 0 (0-0.9) | 0 (0-1) | 0 (0-1.1) | 0 (0-0.3) | 0 (0-0.3) | 0 (0-1.6) | 0 (0-1.1) | 0 (0-0.1) | 0 (0-0.3) | |
| *Unclassified* | 0.2605 | | 0 (0-0.3) | 0 (0-0.4) | 0 (0-0) | 0 (0-0.9) | 0 (0-1) | 0 (0-1.1) | 0 (0-0.3) | 0 (0-0.3) | 0 (0-1.6) | 0 (0-1.1) | 0 (0-0.1) | 0 (0-0.3) | |
| Alteromonadales | 0.7516 | | 0 (0-3.4) | 0 (0-0.4) | 0 (0-0.5) | 0 (0-0) | 0 (0-2.1) | 0 (0-4.8) | 0 (0-1.4) | 0 (0-0.2) | 0 (0-0.5) | 0 (0-0.7) | 0 (0-0.5) | 0 (0-3.4) | |
| Cardiobacteriales | 0.82155 | | 0 (0-1.1) | 0.1 (0-2.8) | 0.4 (0-1.1) | 0 (0-1.5) | 0 (0-0.6) | 0.3 (0-2.2) | 0.2 (0-6.7) | 0.1 (0-3.4) | 0 (0-0.9) | 0 (0-4) | 0 (0-1.3) | 0 (0-1.1) | |
| *Cardiobacteriaceae* | 0.8648 | | 0.4 (0-1.1) | 0.1 (0-2.8) | 0.2 (0-2.1) | 0 (0-1.5) | 0 (0-0.6) | 0.3 (0-2.2) | 0.2 (0-6.7) | 0.1 (0-3.4) | 0 (0-0.9) | 0 (0-4) | 0 (0-1.3) | 0 (0-1.1) | |
| *Unclassified* | 0.8509 | | 0.4 (0-1.1) | 0 (0-2.8) | 0.1 (0-2.1) | 0 (0-1.5) | 0 (0-0.6) | 0 (0-1.7) | 0.2 (0-6.7) | 0 (0-3.4) | 0 (0-0.9) | 0 (0-4) | 0 (0-1.3) | 0 (0-10.2) | |
| Enterobacteriales | 0.2017 | | 0.1 (0-3.4) | 1.8 (0-3.6) | 0.6 (0-7.6) | 0.7 (0-1.5) | 0.7 (0-4.7) | 0.4 (0-2) | 0.8 (0-3.1) | 0.1 (0-2.8) | 1.4 (0-10.5) | 0 (0-2.5) | 0.1 (0-1) | 0.1 (0-3.4) | |
| *Enterobacteriaceae* | 0.1751 | | 0.6 (0-7.6) | 1.8 (0-3.6) | 0.6 (0-6.6) | 0.7 (0-1.5) | 0.7 (0-4.7) | 0.4 (0-2) | 0.8 (0-3.1) | 0.1 (0-2.8) | 1.4 (0-10.5) | 0 (0-2.5) | 0.1 (0-1) | 0 (0-3.4) | |
| *Enterobacter* | 0.7926 | | 0 (0-0.3) | 0 (0-2) | 0 (0-0.1) | 0 (0-0.8) | 0 (0-0.4) | 0 (0-0.3) | 0 (0-0.2) | 0 (0-0.5) | 0 (0-0.8) | 0 (0-1.3) | 0 (0-0.7) | 0 (0-0.1) | |
| *Erwinia* | 0.2640 | | 0.1 (0-6.8) | 0.3 (0-1.5) | 0.5 (0-6.3) | 0.1 (0-0.9) | 0.2 (0-0.8) | 0 (0-1.9) | 0.1 (0-2.7) | 0 (0-1.6) | 1.3 (0-10) | 0 (0-1.3) | 0 (0-1) | 0 (0-3.1) | |
| *Unclassified* | 0.7950 | | 0 (0-0.6) | 0.1 (0-3.3) | 0 (0-1.2) | 0 (0-0.5) | 0.1 (0-4.7) | 0.1 (0-0.3) | 0 (0-1.6) | 0 (0-0.5) | 0.1 (0-0.4) | 0 (0-0.1) | 0 (0-0) | 0 (0-0.4) | |
| Oceanospirillales | **<0.0001** | | 0 (0-0.4) | 0.1 (0-0.7) | 0 (0-0.1) | 0.1 (0-1.2) | 0 (0-0.2) | 0.1 (0-0.6) | 0 (0-0.4) | 0 (0-1.3) | 0 (0-1.3) | 0.1 (0-4) | 0 (0-0) | 0 (0-0.4) | |
| *Halomonadaceae* | **0.0023** | | 0 (0-0) | 0 (0-0.3) | 0 (0-0) | 0 (0-1.1) | 0 (0-0.1) | 0 (0-0.6) | 0 (0-0.4) | 0 (0-1.2) | 0 (0-0) | 0 (0-4) | 0 (0-0) | 0 (0-0.4) | |
| *Halomonas* | **0.0249** | | 0 (0-0) | 0 (0-0.2) | 0 (0-0) | 0 (0-0) | 0 (0-0.1) | 0 (0-0.4) | 0 (0-0.4) | 0 (0-1.2) | 0 (0-0) | 0 (0-4) | 0 (0-0) | 0 (0-0.4) | |
| Pasteurellales | 0.7686 | | 5.1 (0.1-17.9) | 10 (0.5-29.5) | 13 (5.2-27.4) | 3 (0-14.5) | 9.4 (1.5-22) | 9.7 (5.1-31.2) | 4.6 (0-18.7) | 8.3 (2.6-13.4) | 5.5 (0-28.9) | 6.3 (2-20.4) | 8.3 (0-25.5) | 5.1 (0.1-17.9 | |
| *Pasteurellaceae* | 0.7641 | | 13 (5.2-27.4) | 10 (0.5-29.5) | 6.6 (0-15.2) | 3 (0-14.5) | 9.4 (1.5-22) | 9.7 (5.1-31.2) | 4.6 (0-18.7) | 8.3 (2.6-13.4) | 5.5 (0-28.9) | 6.3 (2-20.4) | 8.3 (0-25.5) | 5.1 (0.1-17.9) | |
| *Actinobacillus* | 0.8752 | | 0 (0-0.2) | 0 (0-.8) | 0 (0-0) | 0 (0-0.3) | 0 (0-0.7) | 0 (0-0.1) | 0 (0-0.6) | 0 (0-0.6) | 0 (0-0.1) | 0 (0-0.1) | 0 (0-1) | 0 (0-1.1) | |
| *Aggregatibacter* | 0.9534 | | 0.7 (0-2.7) | 0 (0-9.8) | 0.7 (0-4.2) | 0 (0-2.3) | 0.2 (0-3.4) | 0.1 (0-4.9) | 0.1 (0-3) | 0.3 (0-3.2) | 0 (0-3.2) | 0.2 (0-18.8) | 0.5 (0-1.2) | 0 (0-4.3) | |
| *Haemophilus* | 0.0514 | | 0 (0-0.1) | 0.1 (0-0.7) | 0 (0-0.3) | 0 (0-1) | 0 (0-1.9) | 0.1 (0-1.3) | 0 (0-0.3) | 0 (0-1.3) | 0 (0-3.9) | 0.7 (0-1.3) | 0 (0-0) | 0 (0-1.2) | |
| *Pasteurella* | 0.1459 | | 0.6 (0-1.9) | 0 (0-1.5) | 0 (0-2.2) | 0 (0-1.1) | 0 (0-2.1) | 0.1 (0-1.1) | 0.2 (0-1.2) | 0 (0-0.8) | 0.1 (0-1.4) | 0 (0-3.2) | 0.2 (0-0.7) | 0 (0-1.7) | |
| *Unclassified* | 0.4925 | | 9.4 (5.2-25.4) | 5.1 (0.1-28.9) | 5.7 (0-15.2) | 2.2 (0-12.2) | 7.3 (1.2-20) | 8.2 (0-30.1) | 3 (0-18.7) | 5.8 (0.1-11.9) | 4.4 (0-25.9) | 3.5 (0.1-18.3) | 7.5 (0-25.5) | 2.7 (0.1-17.0) | |
| Pseudomonadales | 0.8952 | | 13.7 (0.2-88.5) | 23 (0-.5-31.1) | 12.3 (3.9-29.4) | 20.3 (0.8-40.1) | 13.1 (3-67) | 20.5 (0.9-34) | 14.2 (4.1-35.1) | 23.8 (0.5-29.4) | 15.2 (3.7-38.2) | 14.2 (1.3-55.2) | 46 (3.2-90.8) | 13.7 (0.2-88.5) | |
| *Moraxellaceae* | 0.4538 | | 6.7 (1.1-24.7) | 4.4 (0-17.4) | 8.2 (0-25.5) | 3.6 (0.6-15.8) | 6.4 (2.1-11.5) | 6 (0.5-12.5) | 10.4 (1.8-16.4) | 3.7 (0.2-13.7) | 6.8 (1.1-18) | 9.1 (0.5-21) | 44.9 (1.8-90.6) | 8.4 (0.2-88.4) | |
| *Acinetobacter* | 0.7274 | | 2.1 (0.1-12.5) | 3.7 (0-9.1) | 4.6 (0-11.6) | 2.8 (0.5-5.9) | 3.2 (0.5-10.9) | 3.2 (0.3-10.6) | 3.8 (013.2) | 2.3 (0-12.8) | 3.2 (0-11.4) | 3.8 (0.3-17) | 0.2 (0-1.8) | 0.2 (0-8.3) | |
| *Enhydrobacter* | 0.1381 | | 1.5 (0-9.8) | 0 (0-5.4) | 0.4 (0-6.6) | 0 (0-1.6) | 0.2 (0-2.6) | 0.1 (0-2.7) | 1 (0-11.5) | 0.4 (0-3.1) | 0 (0-4.7) | 0.3 (0-4.1) | 0.2 (0-1.4) | 0 (0-2.1) | |
| *Moraxella* | 0.5311 | | 1 (0-10) | 0.2 (0-7.3) | 0.8 (0-13.2) | 0.5 (0-9.8) | 0.6 (0-4.9) | 0.3 (0-4.1) | 1.3 (0-3) | 0.1 (0-4) | 0.1 (0-9.7) | 0.9 (0-11.8) | 44.1 (0-89.4) | 0.8 (0-8.3) | |
| *Pseudomonadaceae* | 0.2049 | | 4.7 (0.3-17.2) | 6.1 (0.4-24) | 11.3 (1.2-58.9) | 8.9 (0.1-28.2) | 4.8 (0.9-61.9) | 11.9 (0.4-26.9) | 5.7 (0.4-23) | 10.2 (0.3-24.5) | 4.9 (1.9-26) | 6.7 (0.8-39.3) | 1.4 (0.1-4.7) | 1 (0-.1-24.9) | |
| *Pseudomonas* | 0.6937 | | 2.3 (0.3-13) | 2.5 (0.4-8.8) | 4.8 (0.8-15.4) | 3.3 (0-11.1) | 1.4 (0.7-11) | 3.3 (0.4-12.7) | 2.7 (0.4-9.2) | 4.3 (0-15.6) | 3.3 (0.1-18) | 2.3 (0.7-21.3) | 0.9 (0.1-4.2) | 0.6 (0-7) | |
| *Unclassified* | **0.0257** | | 1.2 (0-5.6) | 4.2 (0-19.4) | 3.7 (0-55.9) | 4.9 (0-22.4) | 0.9 (0-61.3) | 9.1 (0-14.5) | 1.7 (0-21.4) | 4.2 (0-14.3) | 1.6 (0-16.2) | 4.4 (0-17.9) | 0.2 (0-3.4) | 2.8 (0-58.1) | |
| Vibrionales | 0.0748 | | 0 (0-0) | 0 (0-3.2) | 0 (0-0) | 0 (0-0.9) | 0 (0-1.6) | 0 (0-1.4) | 0 (0-0.3) | 0 (0-1.9) | 0 (0-0) | 0 (0-6) | 0 (0-0) | 0 (0-0) | |
| *Vibrionaceae* | **0.0310** | | 0 (0-0) | 0 (0-3.2) | 0 (0-0) | 0 (0-0.9) | 0 (0-1.6) | 0 (0-1.4) | 0 (0-0) | 0 (0-1.9) | 0 (0-0) | 0 (0-6) | 0 (0-0) | 0 (0-0) | |
| *Photobacterium* | 0.2077 | | 0 (0-0) | 0 (0-3.1) | 0 (0-0) | 0 (0-0.2) | 0 (0-1.6) | 0 (0-0) | 0 (0-0) | 0 (0-1.2) | 0 (0-0) | 0 (0-0.4) | 0 (0-0) | 0 (0-0) | |
| *Vibrio* | 0.1372 | | 0 (0-0) | 0 (0-0.2) | 0 (0-0) | 0 (0-0.9) | 0 (0-0) | 0 (0-1.4) | 0 (0-0) | 0 (0-1.9) | 0 (0-0) | 0 (0-6) | 0 (0-0) | 0 (0-0) | |
| Spirochaetes | 0.9915 | | 0.1 (0-1.8) | 0.2 (0-2.3) | 0 (0-3.3) | 0 (0-0) | 0 (0-0.4) | 0 (0-0.9) | 0.2 (0-2.1) | 0 (0-0.6) | 0 (0-1.1) | 0.3 (0-4.7) | 0 (0-0.6) | 0 (0-1.7) | |
| Spirochaetes | 0.9535 | | 0.1 (0-1.8) | 0.2 (0-2.3) | 0 (0-3.3) | 0 (0-0) | 0 (0-0.4) | 0 (0-0.9) | 0.2 (0-2.1) | 0 (0-0.6) | 0 (0-1.1) | 0.3 (0-4.7) | 0 (0-0.6) | 0 (0-1.7) | |
| Spirochaetales | 0.9878 | | 0 (0-1.7) | 0.2 (0-2.3) | 0.1 (0-1.8) | 0 (0-0) | 0 (0-0.4) | 0 (0-0.9) | 0.2 (0-0.9) | 0 (0-0.4) | 0 (0-1.1) | 0.3 (0-4.7) | 0 (0-0.6) | 0 (0-1.7) | |
| *Spirochaetaceae* | 0.9878 | | 0.1 (0-1.8) | 0.2 (0-2.3) | 0 (0-3.3) | 0 (0-0) | 0 (0-0.4) | 0 (0-0.9) | 0.2 (0-0.9) | 0 (0-0.4) | 0 (0-1.1) | 0.3 (0-4.7) | 0 (0-0.6) | 0 (0-1.7) | |
| *Treponema* | 0.9878 | | 0.1 (0-1.8) | 0.2 (0-2.3) | 0 (0-3.3) | 0 (0-0) | 0 (0-0.4) | 0 (0-0.9) | 0.2 (0-0.9) | 0 (0-0.4) | 0 (0-1.1) | 0.3 (0-4.7) | 0 (0-0.6) | 0 (0-1.7) | |
| SR1 | 0.1374 | | 0.3 (0-3.4) | 0 (0-2.6) | 0 (0-3.3) | 0 (0-2.1) | 0 (0-0.5) | 0 (0-0.3) | 0 (0-2) | 0 (0-0.3) | 0 (0-1.4) | 0 (0-1.8) | 0 (0-0.5) | 0 (0-0.4) | |
| Unclassified | 0.0942 | | 0.3 (0-3.4) | 0 (0-2.6) | 0 (0-3.3) | 0 (0-2.1) | 0 (0-0.5) | 0 (0-0.3) | 0 (0-2) | 0 (0-0.3) | 0 (0-1.4) | 0 (0-1.8) | 0 (0-0.5) | 0 (0-0.4) | |
| Unclassified | 0.0982 | | 0 (0-0.4) | 0 (0-2.6) | 0.4 (0-3.4) | 0 (0-2.1) | 0 (0-0.4) | 0 (0-0.3) | 0 (0-2) | 0 (0-0.3) | 0 (0-1.4) | 0 (0-1.8) | 0 (0-0.5) | 0 (0-0.4) | |
| *Unclassified* | 0.1352 | | 0.3 (0-3.4) | 0 (0-2.6) | 0 (0-3.3) | 0 (0-2.1) | 0 (0-0.5) | 0 (0-0.3) | 0 (0-2) | 0 (0-0.3) | 0 (0-1.3) | 0 (0-1.8) | 0 (0-0.5) | 0 (0-0.4) | |
| *Unclassified* | 0.1188 | | 0.3 (0-3.4) | 0 (0-2.6) | 0 (0-3.3) | 0 (0-2.1) | 0 (0-0.5) | 0 (0-0.3) | 0 (0-2) | 0 (0-0.3) | 0 (0-1.4) | 0 (0-1.8) | 0 (0-0.5) | 0 (0-0.4) | |
| Tenericutes | 0.8791 | | 0 (0-1.6) | 0 (0-0.6) | 0 (0-0.1) | 0 (0-0.6) | 0 (0-0.8) | 0 (0-3) | 0 (0-0.8) | 0 (0-0.4) | 0 (0-0.7) | 0 (0-0.2) | 0 (0-0.6) | 0 (0-0.3) | |
| Mollicutes | 0.7474 | | 0 (0-1.6) | 0 (0-0.6) | 0 (0-0.1) | 0 (0-0.6) | 0 (0-0.8) | 0 (0-3) | 0 (0-0.8) | 0 (0-0.4) | 0 (0-0.7) | 0 (0-0.2) | 0 (0-0.6) | 0 (0-0.3) | |
| Mycoplasmatales | 0.7154 | | 0 (0-0.3) | 0 (0-0) | 0 (0-1.6) | 0 (0-0.6) | 0 (0-0.5) | 0 (0-3) | 0 (0-2) | 0 (0-0.2) | 0 (0-0) | 0 (0-0) | 0 (0-0) | 0 (0-0.3) | |
| *Mycoplasmataceae* | 0.9811 | | 0 (0-1.6) | 0 (0-0) | 0 (0-0.1) | 0 (0-0.6) | 0 (0-0) | 0 (0-0.3) | 0 (0-0.8) | 0 (0-0.2) | 0 (0-0) | 0 (0-0) | 0 (0-0) | 0 (0-0.3) | |
| [Thermi] | 0.9005 | | 0 (0-2) | 0 (0-0.9) | 0 (0-0.1) | 0 (0-1.1) | 0 (0-0.4) | 0 (0-0.6) | 0 (0-0.9) | 0 (0-0.4) | 0 (0-0.9) | 0 (0-0.2) | 0 (0-0) | 0 (0-2) | |
| Deinococci | 0.8420 | | 0 (0-2) | 0 (0-0.9) | 0 (0-0.1) | 0 (0-1.1) | 0 (0-0.4) | 0 (0-0.6) | 0 (0-0.9) | 0 (0-0.4) | 0 (0-0.9) | 0 (0-0.2) | 0 (0-0) | 0 (0-2) | |
| Deinococcales | 0.6896 | | 0 (0-2) | 0 (0-0.9) | 0 (0-2) | 0 (0-1.1) | 0 (0-0) | 0 (0-0.6) | 0 (0-0.9) | 0 (0-0.4) | 0 (0-0.9) | 0 (0-0.2) | 0 (0-0) | 0 (0-2) | |
| *Trueperaceae* | 0.3513 | | 0 (0-2) | 0 (0-0.9) | 0 (0-0.1) | 0 (0-1.1) | 0 (0-0) | 0 (0-0) | 0 (0-0.2) | 0 (0-0.4) | 0 (0-0) | 0 (0-0.2) | 0 (0-0) | 0 (0-2) | |
| *Truepera* | 0.2196 | | 0 (0-2) | 0 (0-0.9) | 0 (0-0.1) | 0 (0-1.1) | 0 (0-0) | 0 (0-0) | 0 (0-0.2) | 0 (0-0.4) | 0 (0-0) | 0 (0-0.2) | 0 (0-0) | 0 (0-2) | |
| Verrucomicrobia | 0.5043 | | 0 (0-2.9) | 0 (0-0.2) | 0 (0-0) | 0 (0-.07) | 0 (0-1.3) | 0 (0-0) | 0 (0-3.9) | 0 (0-2.3) | 0 (0-1.3) | 0 (0-0) | 0 (0-0.1) | 0 (0-0.6) | |
| Verrucomicrobiae | 0.1128 | | 0 (0-0.1) | 0 (0-0.2) | 0 (0-0) | 0 (0-0.1) | 0 (0-1) | 0 (0-0) | 0 (0-3.9) | 0 (0-0.3) | 0 (0-1.3) | 0 (0-0) | 0 (0-0.1) | 0 (0-0) | |
| Verrucomicrobiales | 0.1253 | | 0 (0-0) | 0 (0-0.2) | 0 (0-0) | 0 (0-0.1) | 0 (0-1) | 0 (0-0) | 0 (0-3.9) | 0 (0-0.3) | 0 (0-1.3) | 0 (0-0) | 0 (0-0.1) | 0 (0-0) | |
| *Verrucomicrobiaceae* | 0.1428 | | 0 (0-0.1) | 0 (0-0.2) | 0 (0-0) | 0 (0-0.1) | 0 (0-1) | 0 (0-0) | 0 (0-3.9) | 0 (0-0.3) | 0 (0-1.3) | 0 (0-0) | 0 (0-0.1) | 0 (0-0) | |
