# Supplementary material for: The feline skin microbiota: The bacteria inhabiting the skin of healthy and allergic cats
Source: PLoS One. 2017 Jun 2;12(6):e0178555. doi: 10.1371/journal.pone.0178555 (PMC5456077; doi:10.1371/journal.pone.0178555)
Supplement: S5 Table — Allergy treatments were concurrent. All cats with a Y in the steroids column had previously received steroids, except for F14 and F15 that were receiving steroids at the time of sampling. F18 was diagnosed with a ringworm infection, treated with limed dips, and lesions resolved three months prior to sample collection. DSH-Domestic short hair, Per-Persian, Sia-Siamese, FBH-Flea bite hypersensitivity, FIHD-Food induced hypersensitivity, NFNFIHD- Non-flea non-food induced hypersensitivity, NFBH- non-flea bite hypersensitivity, G-gradual, Y-Yes, N-No. Modified from Meason-Smith C, Diesel A, Patterson AP, Older CE, Johnson TJ, Mansell JM, et al. Characterization of the cutaneous mycobiota in healthy and allergic cats using next generation sequencing. Vet Dermatol. 2016:1–11. (DOCX) [file pone.0178555.s005.docx]

Table S5. Hypersensitivity information (type, age of onset, seasonality, clinical signs, lesion distribution, ear problems, and treatments for ten allergic cats.

| Cat | Breed | Age | Type | Age of onset | Seasonality | Clinical signs | Lesions distribution | Ear problems | Allergy treatments | Steroids |
| --- | --- | --- | --- | --- | --- | --- | --- | --- | --- | --- |
| F12 | DSH | 9 | FBH | 6 | N | Pruritis, self induced alopecia | Limbs | N | N | N |
| F13 | Sia | 8 | FBH | 6 | N | Pruritis, self induced alopecia | Dorsum | N | N | N |
| F14 | DSH | 11 | FBH | G | Summer | Pruritis, self induced alopecia, crusting | Rump, tail, ears, ventral abdomen | Y | N | Y |
| F15 | Sia | 9 | FBH, FIHD, NFNFIHD | 3 | Spring, Summer | Pruritis, self induced alopecia, cervicofacial | Face, neck, ears | Y | Cyclosporine | Y |
| F16 | DSH | 5 | FBH, NFNFIHD | 4 | N | Pruritis, self induced alopecia, excoriations | Chest, ventral abdomen, dorsum, tail, limbs | Y | Cyclosporine, antihistamines | Y |
| F17 | DSH | 9 | FBH, NFNFIHD | 6 | N | Pruritis, self induced alopecia, cervicofacial, eosinophilic | Face, ventral abdomen, limbs | N | Sublingual immunotherapy | Y |
| F18 | Per | 4 | FBH, NFBH | 3 | N | Pruritis, self induced alopecia | Ears, ventral abdomen, rump, tail, limbs | Y | N | N |
| F19 | DSH | 11 | FBH | 9 | N | Self induced alopecia, excoriation | Ventral abdomen, dorsum | Y | N | Y |
| F20 | DSH | 7 | NFNFIHD | 6 | N | Pruritis, cervicofacial | Face, ears | N | N | Y |
| F21 | DSH | 8 | FBH | G | N | Pruritis, self induced alopecia | Vental abdomen, limbs | N | N | N |

Allergy treatments were concurrent. All cats with a Y in the steroids column had previously received steroids, except for F14 and F15 that were receiving steroids at the time of sampling. F18 was diagnosed with a ringworm infection, treated with limed dips, and lesions resolved three months prior to sample collection.

DSH-Domestic short hair, Per-Persian, Sia-Siamese, FBH-Flea bite hypersensitivity, FIHD-Food induced hypersensitivity, NFNFIHD- Non-flea non-food induced hypersensitivity, NFBH- non-flea bite hypersensitivity, G-gradual, Y-Yes, N-No.

Modified from Meason-Smith C, Diesel A, Patterson AP, Older CE, Johnson TJ, Mansell JM, et al. Characterization of the cutaneous mycobiota in healthy and allergic cats using next generation sequencing. Vet Dermatol. 2016:1-11.
