# Supplementary figures and images for: The feline skin microbiota: The bacteria inhabiting the skin of healthy and allergic cats
Source: PLoS One. 2017 Jun 2;12(6):e0178555. doi: 10.1371/journal.pone.0178555 (PMC5456077; doi:10.1371/journal.pone.0178555)

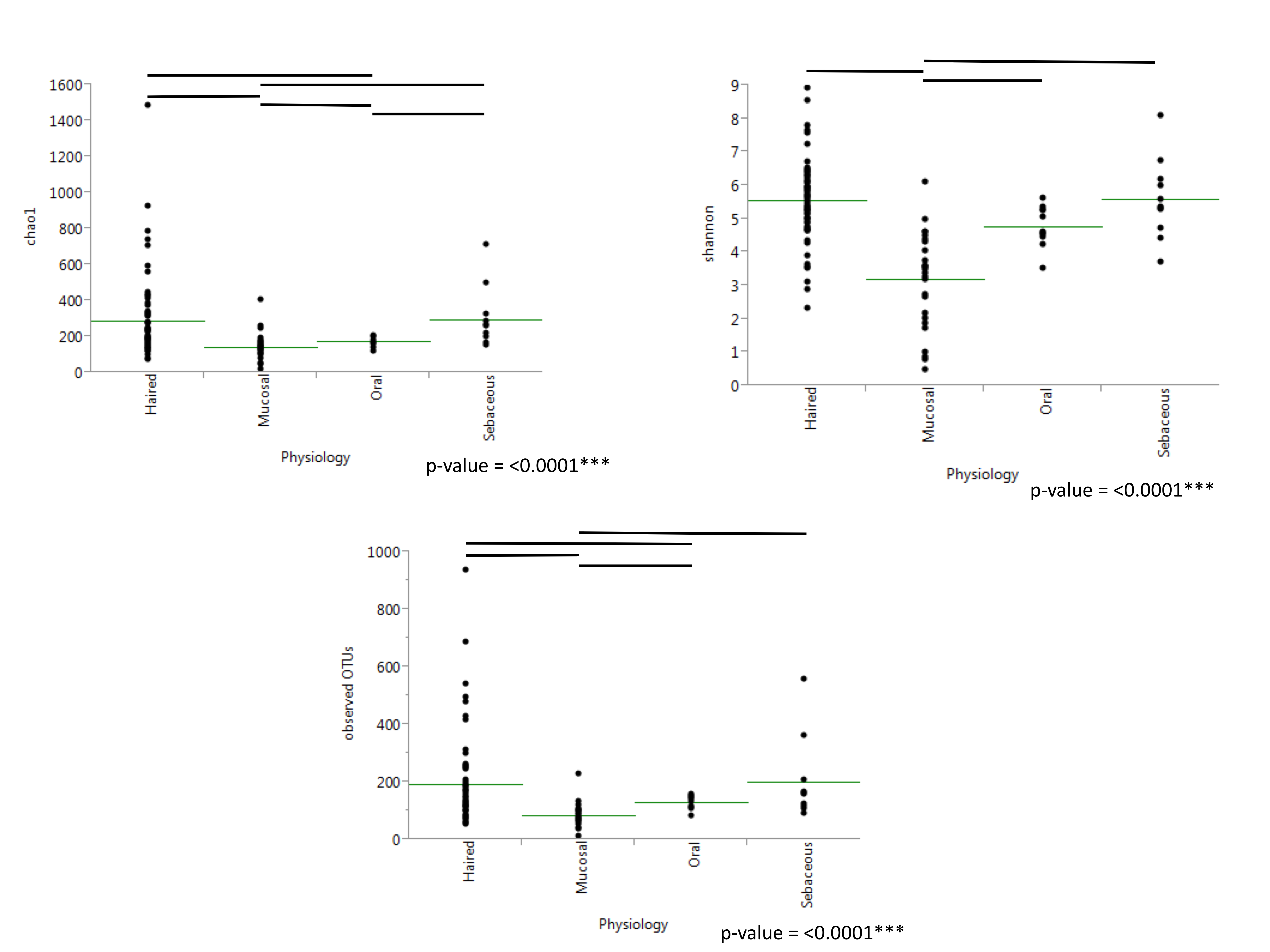

Supplement: S1 Fig — P-values are from Kruskal-Wallis tests. Lines indicate pairwise comparisons that resulted in significant p-values. (TIF) [file pone.0178555.s006.tif]

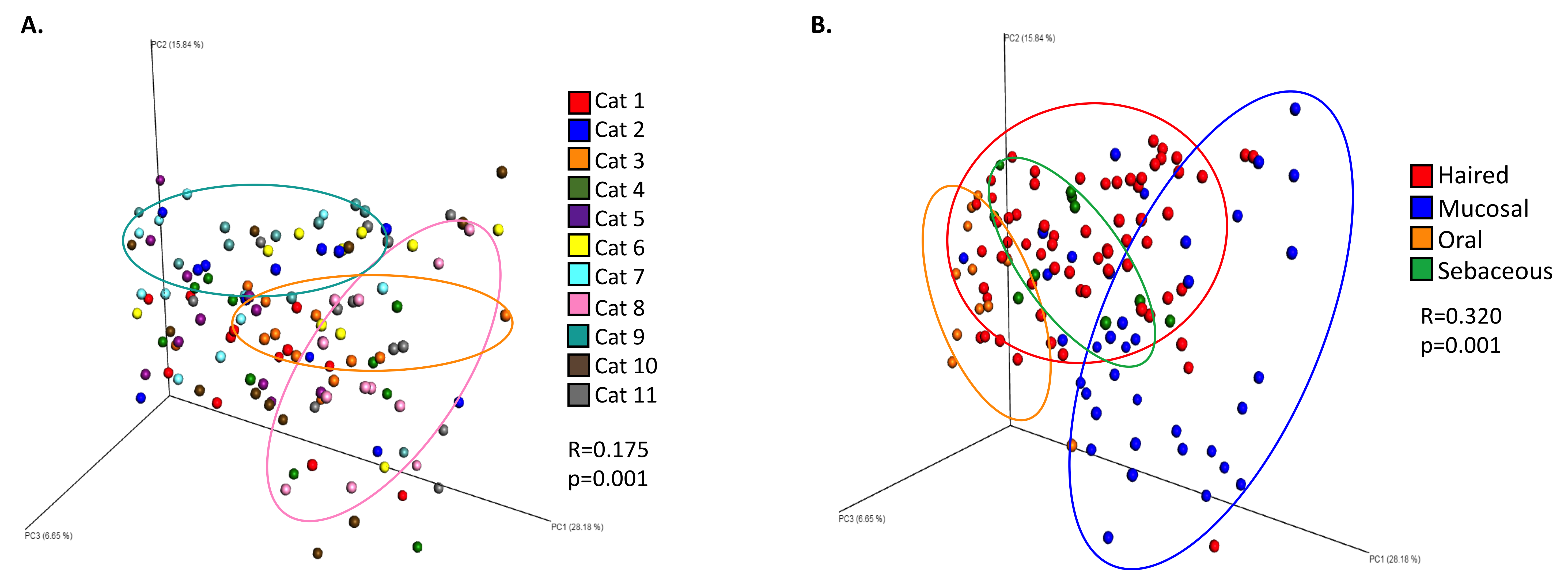

Supplement: S2 Fig — Principal coordinate analysis plots of Weighted UniFrac distance matrices of healthy cat samples by A. individual cat and B. physiology. (TIF) [file pone.0178555.s007.tif]

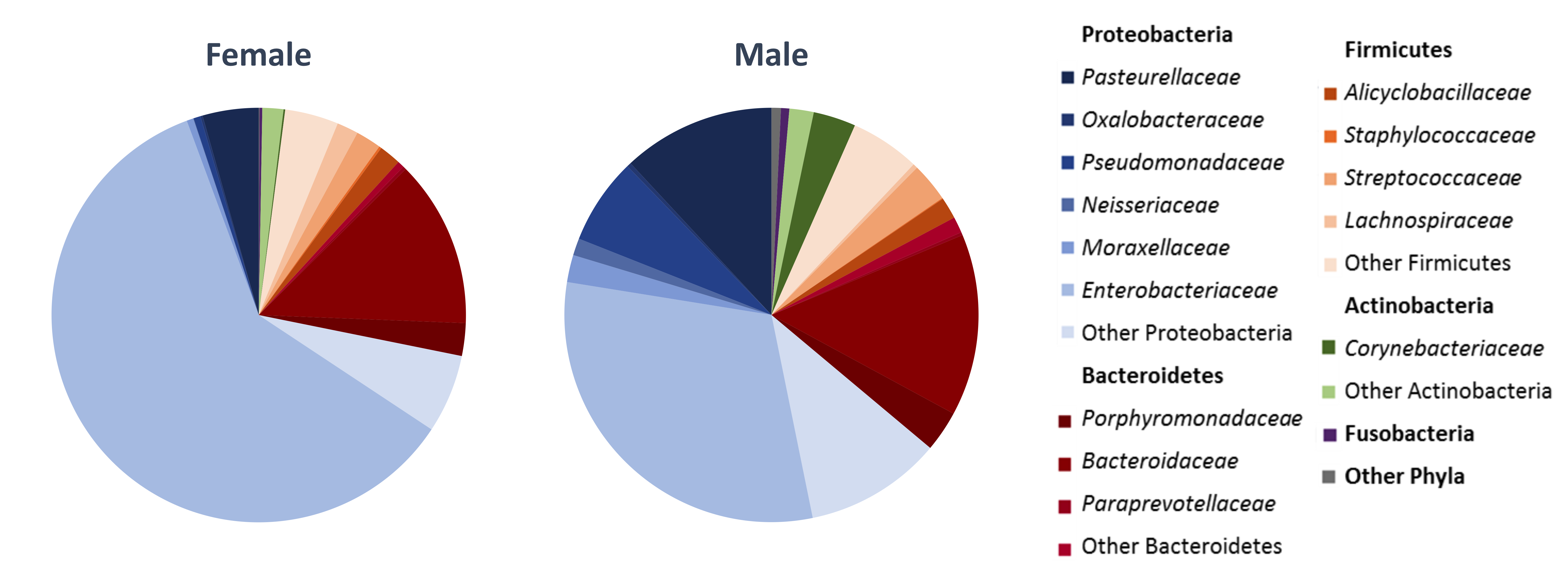

Supplement: S3 Fig — (TIF) [file pone.0178555.s008.tif]

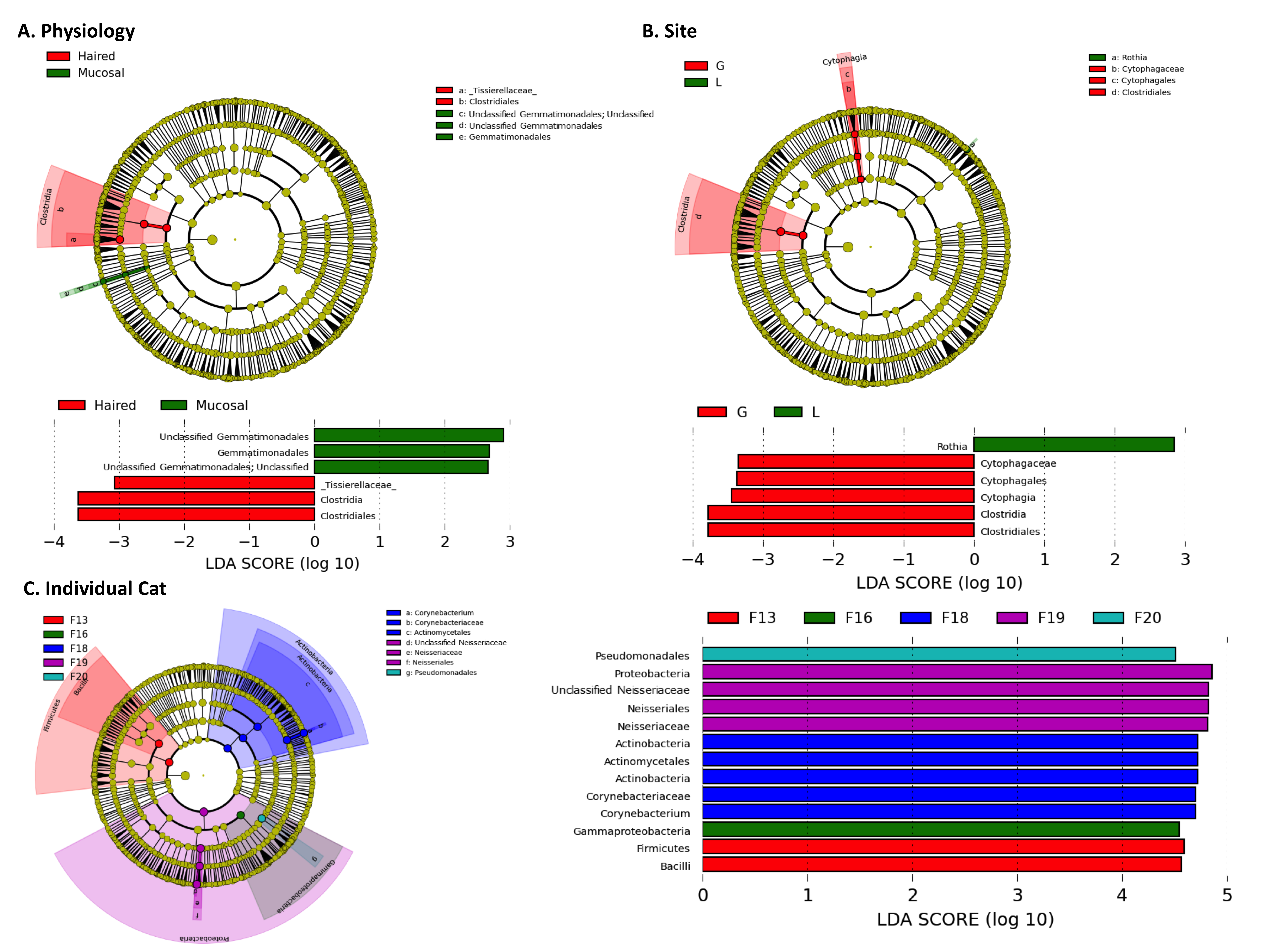

Supplement: S4 Fig — (TIF) [file pone.0178555.s009.tif]

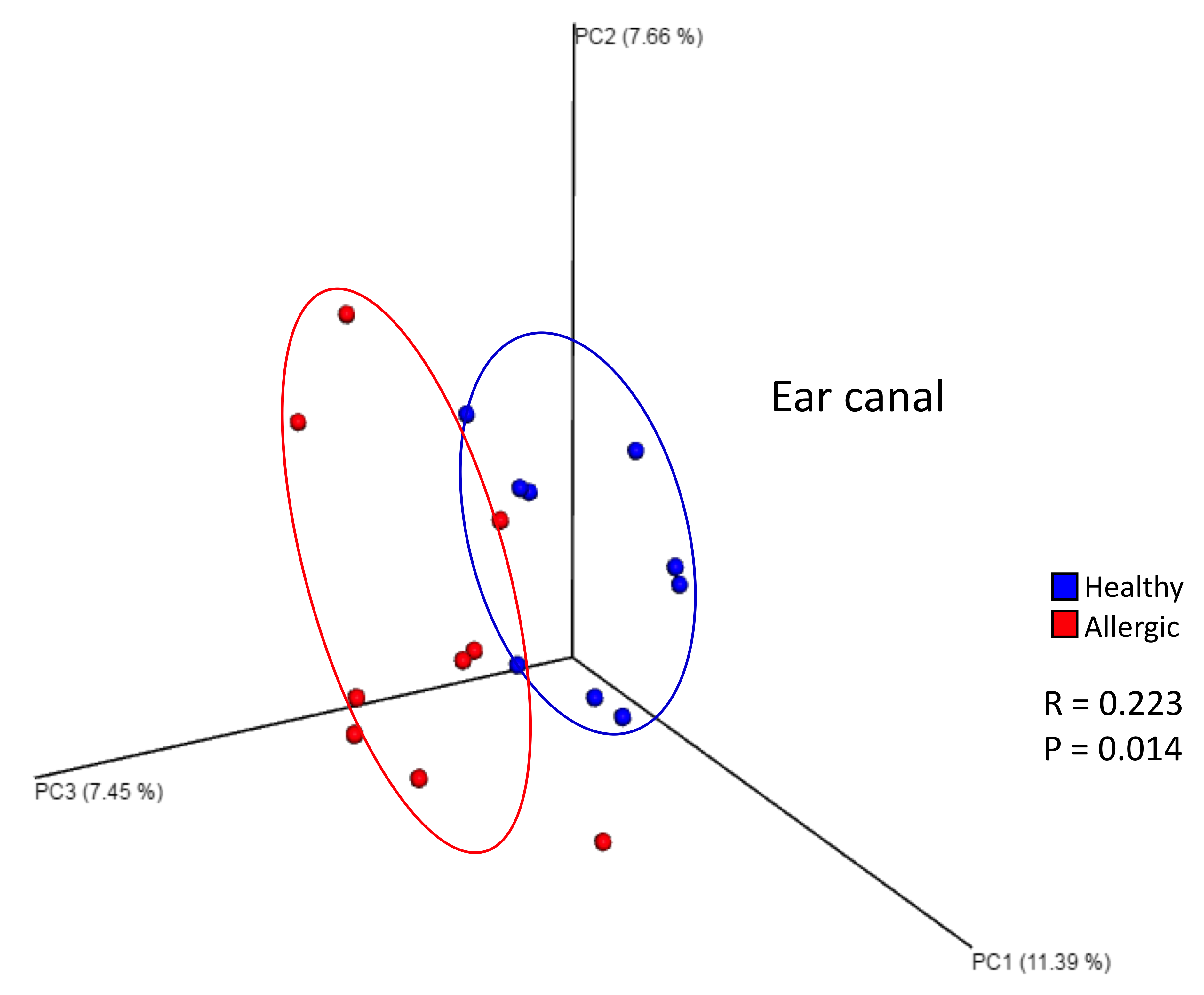

Supplement: S5 Fig — Principal coordinate analysis plot based on Bray-Curtis distance matrix comparing healthy and allergic ear canal samples. Healthy and allergic ear canal samples showed distinct clustering. (TIF) [file pone.0178555.s010.tif]

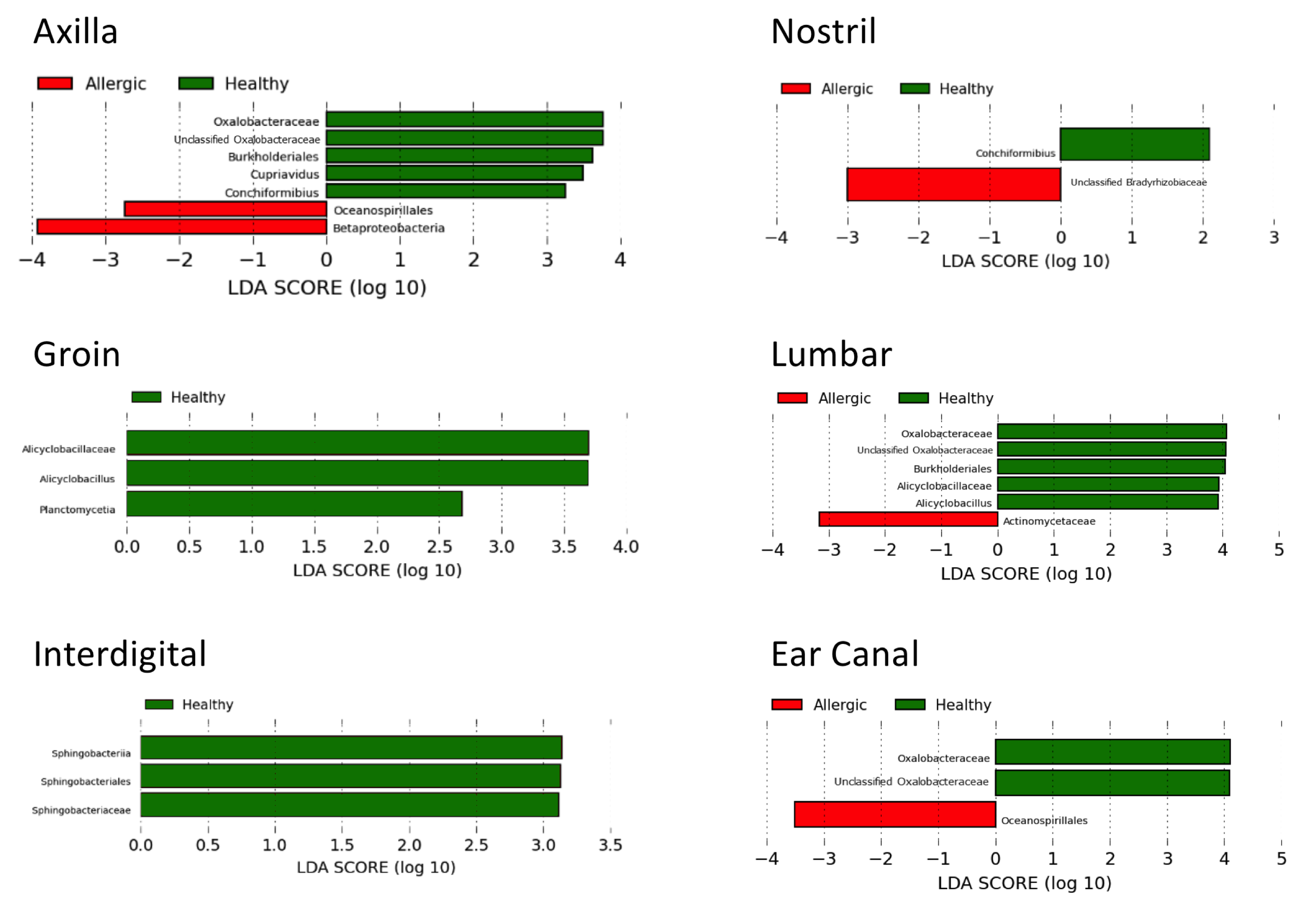

Supplement: S6 Fig — (TIF) [file pone.0178555.s011.tif]
